# Joint single-cell profiling of Cas9 edits and transcriptomes reveals widespread off-target events and effects on gene expression

**DOI:** 10.1101/2025.02.07.636966

**Published:** 2025-08-28

**Authors:** Michael H. Lorenzini, Brad Balderson, Karthyayani Sajeev, Aaron J. Ho, Graham McVicker

**Affiliations:** 1 Integrative Biology Laboratory, Salk Institute for Biological Studies, La Jolla, CA, USA; 2 Biomedical Sciences Graduate Program, University of California San Diego, La Jolla, CA, USA

## Abstract

A longstanding barrier in genome engineering with CRISPR-Cas9 has been the inability to measure Cas9 edit outcomes and their functional effects at single-cell resolution. Here we present Superb-seq, a new technology that leverages T7 *in situ* transcription and single-cell RNA sequencing to jointly measure on- and off-target Cas9 edits and their effects on gene expression. We performed Superb-seq on 10,000 K562 cells, targeting four chromatin remodeler genes with seven guide RNAs. Superb-seq identified 11,891 edit events in 6,230 edited cells at all seven on-target sites and at an additional 36 off-target sites. Although the seven guides were selected for high specificity, six of them caused off-target edits, ranging in frequency from 0.03% to 18.6% of cells. A notable off-target edit within the first intron of *USP9X* disrupted the expression of this gene and over 150 downstream genes. In summary, Cas9 off-targeting is pervasive due to a combination of rare and common edit events, occurs primarily within introns of off-target genes, and can exert widespread effects on gene expression. Superb-seq uses off-the-shelf kits, standard equipment, and requires no virus, which will enable genome-wide CRISPR screens in diverse cell types as well as functional characterization of clinically-relevant guides.

## Introduction

Cas9, the RNA-guided CRISPR nuclease, is a fundamental tool for targeted manipulation of genome sequences, with broad applications ranging from functional genomics to gene therapy. Recently, single-cell CRISPR experiments have emerged as an important method to study gene regulation^1^. In these experiments, known as Perturb-seq, hundreds to thousands of different guide RNAs are delivered into cells, and then guide identities are linked to transcriptome profiles by single-cell RNA sequencing (scRNA-seq)^2–5^. However, a longstanding technical barrier in CRISPR-Cas9 functional genomics has been a limited ability to characterize Cas9 editing outcomes at single-cell resolution. In pooled Perturb-seq screens, perturbation outcomes are determined by sequencing each cell’s guide RNA and endogenous transcriptome^2–6^. Unfortunately, guides vary widely in their perturbation efficiency^7,8^, and as many as 50% of Perturb-seq guides exhibit no detectable activity at their on-target site^9,10^. Additionally, most guides exhibit “off-target” activity^7,11–13^ that limits CRISPR screen efficacy^14^. Since Perturb-seq reads out guides instead of Cas9 edits, the downstream effects of off-target perturbations are ambiguous, which can lead to incorrect interpretation of causal edits^6,10^.

In therapeutics, a Cas9 gene therapy recently received regulatory approval to treat severe blood disorders^15^, and clinical trials are broadly evaluating Cas9 editing of additional tissues^16^. However, potential off-target genotoxicities and cellular side effects remain largely unclear. Currently, off-target edit sites are identified either by computational predictions^17–20^ or by experimental sequencing methods that tag double-strand breaks in live cells (e.g. GUIDE-seq)^21–23^ or chromatin immuno-precipitate break repair proteins^24–26^. However, no method currently exists to assess the downstream consequences of off-target edits on gene expression and cell function. In addition to missing downstream effects, bulk DNA sequencing methods may miss edits that occur in smaller fractions of cells, and they are difficult to scale for parallel off-target profiling of many guides. Finally, computational analysis indicates that off-target events are likely to vary substantially across genetic backgrounds^27^, but current empirical methods lack the requisite scalability to compare Cas9 editing outcomes in cohorts of many donor genomes. Off-target sequence alterations by retroviral gene therapies have unfortunately triggered genotoxic events including oncogene activation and cancer^28^. Therefore, there is a critical need for experimental methods that can perform transcriptomic characterization of Cas9 off-target events.

To address these limitations, we have developed **“Superb-seq”**, a high-throughput single-cell method for joint Cas9 edit capture and transcriptome profiling. In contrast to Perturb-seq, which detects guides in individual cells, Superb-seq detects Cas9 edits directly by leveraging T7 transcription, the two-component system of phage T7 promoter and RNA polymerase. Superb-seq performs *in situ* transcription (IST) of T7 RNA within intact fixed cells^29–32^ to mark both on-target and off-target Cas9 edit sites. The exogenous T7 transcripts, together with endogenous cellular transcripts, are read out with scRNA-seq and analyzed to identify Cas9 edits and their effects on the transcriptome.

**Figure 1.**
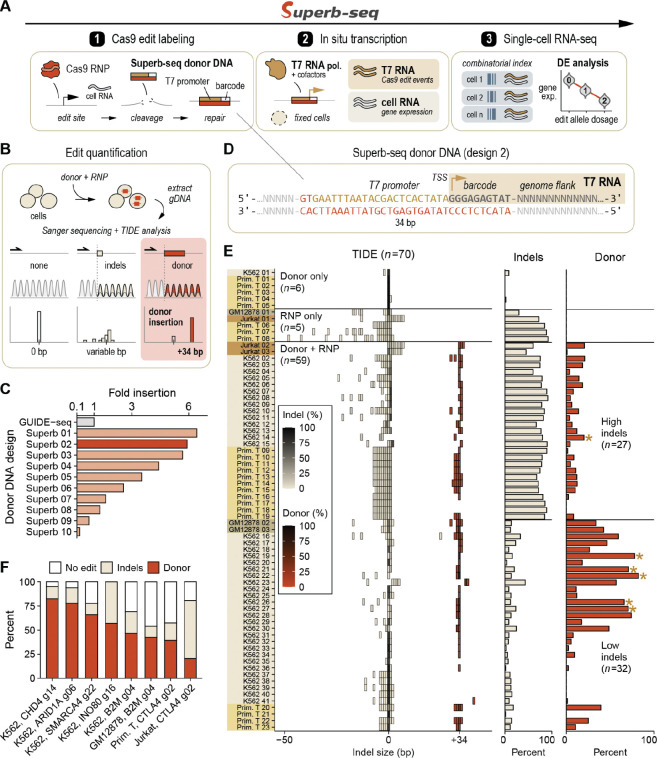
Homology-free labeling of Cas9 genome edits with phage T7 promoter. (**A**) Superb-seq has three steps: Cas9 edit labeling with T7 promoters, *in situ* transcription of edit-marking T7 RNA in fixed cells, and joint combinatorial scRNA-seq of T7 and cellular RNA. (**B**) Quantification of promoter-labeled Cas9 edits by Sanger sequencing and TIDE analysis^42^. (**C**) Insertion rate of the top 10 candidate Superb-seq donor DNA designs relative to the GUIDE-seq donor, measured by TIDE. Value for design 02 is the mean of four samples. (**D**) Sequence of the 34 bp Superb-seq donor DNA (design 02) encoding an optimized T7 promoter and T7-transcribed barcode sequence. (**E**) TIDE analysis of Cas9 edit outcomes in 70 donor-edited or control samples with R^2^ > 0.5 (n=6 donor only, n=5 RNP only, n=59 donor+RNP). Donor insertion is defined as +30 bp insertion or greater (Donor), and indel without donor is defined as any other edit event (Indels). Asterisks (*) indicate K562 cell lines selected for Superb-seq. (**F**) Frequencies of unedited (none), variable untemplated indels, and donor insertion alleles in four cell types treated with donor+RNP targeting six genome sites.

Superb-seq is performed in three steps with standard laboratory equipment, off-the-shelf kits, and no virus (Fig. 1A). First, Cas9 edit sites are labeled by the insertion of T7 promoter sequences in live cells through electroporation of CRISPR-Cas9 complexes and donor DNA. Second, cells are fixed and T7 IST is performed to generate edit-reporting T7 RNAs. Third, T7 and endogenous RNAs are sequenced in single cells by combinatorial scRNA-seq. Superb-seq enables high-throughput single-cell Cas9 cleavage profiling, guide-specific off-target sequence analysis, single-cell counting of edit alleles, and association of on- and off-target edit events with differential transcriptome expression. Here we present the development of Superb-seq, results from two Superb-seq libraries generated in K562 cells, and a companion software package for data analysis named **“Sheriff”**.

## Results

### Homology-free labeling of genome edits with T7 promoter sequences

To develop the first step of Superb-seq, we established the ability to label Cas9 edits with T7 promoter sequences. We chose a homology-free knock-in strategy, in which live cells are electroporated with *S. pyogenes* Cas9-guide RNA ribonucleoproteins (RNPs) and a double-stranded donor DNA composed of synthetic annealed oligonucleotides (also known as dsODNs). The RNPs generate a double-strand break in the genome^33,34^, and donor DNA is inserted at the break by non-homologous end joining^35,36^ (Fig. 1A). Homology-free insertion has advantages over homology-directed repair because it enables the labeling of both on-target and off-target Cas9 edits with the same donor sequence^21^, and because it has broader compatibility with non-dividing cell types^37^.

We made a panel of 27 homology-free donor DNA constructs of length 19–55 bp encoding T7 and SP6 phage promoter sequence variants^38–41^ (Supp. Fig. 1A, Supp. Table 1). We quantified the insertion efficiency of each donor by TIDE (tracking indels by decomposition)^42^ (Fig. 1B) and compared labeling efficiency to a published GUIDE-seq donor sequence that is commonly applied to primary cell gene therapies^43^. The insertion efficiency varied widely across donor designs, with over 60% (17/27) of designs failing to generate detectable knock-in (Supp. Fig. 1B), suggesting that donor insertion is highly sensitive to donor length and composition. However, three designs (donors 01, 02, 03) achieved strong knock-in, with 5.7–6.4 fold higher efficiency than the GUIDE-seq donor (Fig. 1C). These top-performing donor sequences combined elements of the GUIDE-seq donor and of variant T7 promoter sequences used for Cel-seq+^41^(Supp. Fig. 1C, Supp. Table 1). We selected design 02 for further Superb-seq development since its promoter was previously shown to improve scRNA-seq library amplification^41^(Fig. 1D). Importantly, this donor disrupts target gene expression by triggering nonsense-mediated decay (NMD) of coding sequences (Supp. Fig. 2, Supplementary Note 1).

To establish that Superb-seq donor DNA efficiently labels genome edits in a variety of cellular contexts, we performed a total of 112 Cas9 edits with four human cell types (GM12878, Jurkat, K562, and primary T cells) and 22 guide RNAs targeting eight different genome loci: *ARID1A*, *B2M*, *CHD3*, *CHD4*, *CTLA4*, *INO80*, *SMARCA4*, and *TRAC* (Supp. Tables 1, 2). We then characterized the editing outcomes in 70 samples (11 controls, 59 donor knock-ins mostly with design 02) that yielded reliable TIDE results (R^2^ > 0.5) (Fig. 1E, Supp. Fig. 1D). As expected, treatment with donor DNA alone resulted in unedited sequences. Treatment with Cas9 RNPs without donor DNA resulted in generally short indels with occasional longer deletions approaching −50 bp. In contrast, treatment with both donor DNA and RNPs resulted in consistent +34 bp insertion events, indicating successful labeling of Cas9 edits with the Superb-seq donor sequence in a variety of cell and genome contexts (Fig. 1E). High donor insertion efficiency of 40–80% was achieved at six genome regions and in all cell types except Jurkat cells (Fig. 1F), possibly due to Jurkat overexpression of end-joining machinery that favors rapid, imprecise indel formation^44^. Excluding Jurkats, donor insertion decreased the formation of variable indels and larger deletions (Supp. Fig. 1E), consistent with the recent observation that homology-free labeling counteracts cytotoxic chromosome rearrangement^45^. Donor insertion was accompanied by a distinct low or high rate of small indels, based on the particular guide RNA used (Supp. Fig. 3, Supplementary Note 2). The size of donor insertion events was consistently within a few base pairs of +34 bp (Supp. Fig. 2C), demonstrating successful full-length T7 promoter labeling in a variety of Cas9 editing contexts.

### *In situ* transcription (IST) enables direct edit capture by RNA sequencing

**Figure 2.**
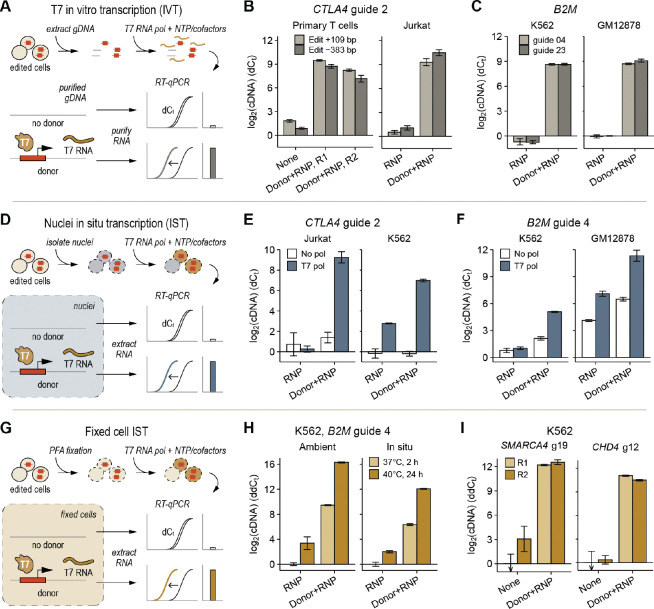
Development of *in situ* transcription of Cas9 genome edits. (**A**) *In vitro* transcription (IVT) on T7 promoter-labeled genomic DNA and quantification by RT-qPCR. (**B,C**) Quantity of IVT transcripts from genomic DNA treated with Cas9 ribonucleoproteins without donor DNA (RNP), RNPs with donor DNA (donor+RNP), or neither (none), measured by RT-qPCR and the comparative cycle threshold (C_t_) method^48^. Cas9 edits were generated at the (**B**) *CTLA4* or (**C**) *B2M* locus in the indicated cell types, IVT replicates (R1, R2), PCR assay position relative to edit site (e.g. Edit +109 bp), and/or the identity of the guide used (e.g. guide 4). (**D**) *In situ* transcription (IST) on unfixed nuclei containing T7 promoter-labeled chromatin. (**E,F**) Quantity of T7 RNA from nuclei IST with T7 RNA polymerase (T7 pol) or mock IST without polymerase (No pol) in the indicated cell types. (**G**) IST on fixed cells containing T7 promoter-labeled chromosomes. (**H**) Quantity of T7 RNA in IST supernatant (ambient) or cell pellet (*in situ*) fraction, before and after optimizing IST reaction conditions. (**I**) Quantity of T7 RNA from replicate fixed-cell IST reactions targeting *SMARCA4* and *CHD4* in K562 cells. For IVT and nuclei IST (**B,C,E,F**), results are represented as a difference of C_t_ values (dC_t_) normalized to background signal at two non-transcribed genomic safe harbor (GSH) loci^49^. For fixed-cell IST (**H,I**), GSH background was below detection, so results are represented as a difference of differences (ddC_t_) normalized to reference genes *RPL24/RPS10* and treatment without donor (RNP or none). Bars represent the mean of technical replicates (n=3), and whiskers represent 2 × SEM.

For the next step of Superb-seq, we established that inserted donor DNA generates Cas9 edit-marking T7 transcripts in fixed cells. We developed this step systematically by starting with *in vitro* transcription on purified genomic DNA, testing IST in unfixed nuclei, and then optimizing IST for paraformaldehyde (PFA)-fixed cells (Fig. 2, Supplementary Note 3). Across multiple cell types and genome sites, we observed strong induction of T7 RNA at Cas9 edit sites of up to 1,000 fold by reverse transcription quantitative PCR (RT-qPCR, Fig. 2A–F). T7 signals were promoter-specific and polymerase-specific (with some background signal in GM12878 cells due to high *B2M* gene expression). Importantly, the majority of T7 transcripts were short (< 1 kilobase), bi-directional, and tightly localized to expected Cas9 edit sites (Supp. Figs. 4, 5), consistent with bi-directional promoter orientation from homology-free donor insertion. These results establish that donor-inserted T7 promoters are effective at generating edit-marking T7 transcripts.

We adapted T7 IST for PFA fixation. Previous work developed T7 IST for methanol-fixed cells^29–31^. However, we reasoned that PFA fixation would enable downstream single-cell analysis by high-throughput SPLiT-seq, an off-the-shelf scRNA-seq chemistry for highly scalable combinatorial barcoding of PFA-fixed cells^46^. SPLiT-seq uses blended random and oligo-dT primers, enabling joint capture of non-polyadenylated T7 RNA and polyadenylated mRNA into one sequencing library. Under optimal IST incubation conditions for PFA-fixed cells (40°C, 24 hours, 10 mM NTPs), we achieved levels of T7 RNA up to 16 fold higher than *RPL24* mRNA, an extremely expressed ribosome subunit, with minimal increase in background signal (Fig. 2G–I, Supp. Fig. 6). Importantly, high levels of T7 RNA were retained in the cell pellet fractions of T7 IST reactions and were resistant to washout (Fig. 2H, Supp. Fig. 6D), indicating that T7 transcripts are suitable to be carried through downstream single-cell barcoding. To determine whether IST is efficient at different genome sites, we performed T7 IST with seven guide RNAs targeting four loci encoding chromatin remodeler genes (*ARID1A*, *SMARCA4*, *CHD3*, *CHD4*). We observed high T7 transcription by RT-qPCR at all loci except *ARID1A* (Fig. 2I, Supp. Fig. 7), where both edit sites exhibited low T7 expression despite high donor insertion efficiency (Fig. 1F), suggesting that IST efficiency can vary between genome sites.

Next we established that Cas9 edit sites can be identified by bulk RNA sequencing of T7 transcripts (Supp. Fig. 8A). We took two cell types (Jurkat and K562) edited with T7 promoter labeling at two genome sites (*CTLA4* and *B2M*) and performed nuclei isolation and IST. We then generated a 3’ random hexamer-primed RNA-seq library to match the capture chemistry of SPLiT-seq libraries, and performed low-depth, paired-end sequencing. In the absence of donor DNA (RNP) or T7 RNA polymerase (No pol), no reads were observed at either Cas9 edit site (Supp. Fig. 8B,C). However, cells that received both donor DNA (donor+RNP) and T7 polymerase (T7 pol) exhibited read pileups centered at expected Cas9 cut sites. Reads began with T7 barcode sequences encoded by inserted donor DNA (5’-GGGAGAGTAT-3’), followed by genome-aligned sequence that began at the exact position of expected Cas9 cleavage, 3 bp upstream of the protospacer activation motif (PAM)^47^ (Supp. Fig. 8B,C). Each edit site showed divergent, opposite-stranded reads that mapped within 200 bp of expected edit sites, consistent with RT-qPCR observations of short, bi-directional T7 RNAs (Supp. Fig. 4B–D). Some reads began less than 100 bp from the edit site but lacked T7 barcodes, likely from truncated T7 cDNA caused by library fragmentation. Together, these results establish that Cas9 genome edits can be identified by IST and T7 transcript sequencing.

### Superb-seq generates high-quality single-cell RNA-seq reads

**Figure 3.**
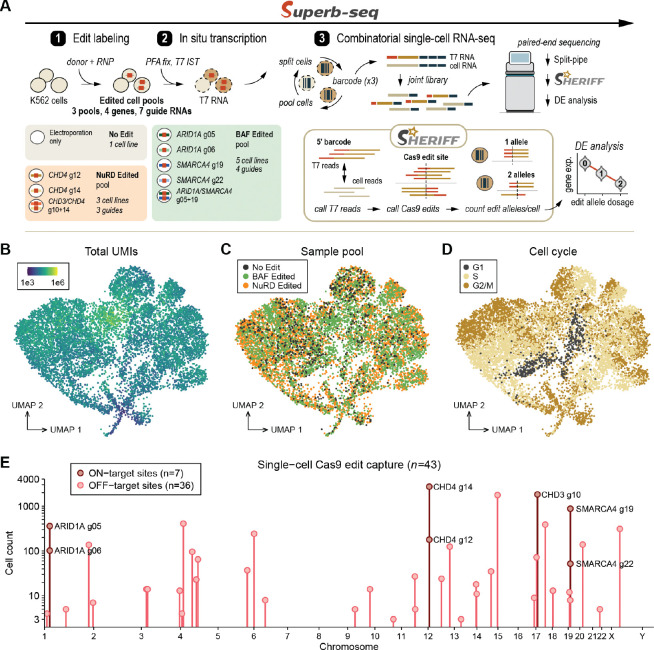
Joint single-cell profiling of genome edits and transcriptomes by Superb-seq. (**A**) Diagram of a Superb-seq experiment on 10,000 K562 cells targeting four chromatin remodeler genes with seven guide RNAs. *In situ* transcription and SPLiT-seq combinatorial barcoding was performed on three pools of unedited, *ARID1A*/*SMARCA4*-edited, and *CHD3/CHD4*-edited cell lines. Paired-end sequencing reads were analyzed by Split-pipe and custom Sheriff software. (**B–D**) UMAP clustering of 9,500 K562 cells analyzed by Sheriff. Cells are colored by (**B**) total count of unique molecular identifiers (UMIs), (**C**) sample pool, or (**D**) cell cycle score from Scanpy^52^. (**E**) Direct single-cell capture of Cas9 edits at all seven on-target sites and 36 off-target sites in 6,230 edited cells, quantified by Sheriff.

We next established the complete end-to-end protocol of T7 promoter labeling, IST, and scRNA-seq that we call **“Superb-seq”** (Fig. 3A). We prepared K562 cell lines with T7 promoter-labeled Cas9 edits by Cas9 RNP electroporation, using seven guide RNAs that target the coding regions of four chromatin remodeler genes (Fig. 3A, Supp. Table 1). We targeted BAF complex members *ARID1A* and *SMARCA4*, and NuRD complex members *CHD3* and *CHD4*^50^. We generated three sample pools consisting of: (1) unedited electroporated K562 cells, (2) five cell lines that were either single-targeted (one guide) or double-targeted (two guides) at *ARID1A* and/or *SMARCA4*, and (3) three cell lines either single-targeted at *CHD4* or double-targeted at *CHD3*/*CHD4* (Fig. 3A). For each sample pool, we chose cell lines treated with “low indel” guides that gave 75% labeling of T7 promoters at the target site, estimated by TIDE (Fig. 1E, Supp. Fig. 9A). To test single-cell edit capture in the event of lower labeling or T7 transcription efficiency, we also included one cell line (*CHD3*/*CHD4* double-targeted) with 25% promoter labeling at *CHD3*, and we included the *ARID1A*-edited cell lines described above (Supp. Fig. 6A).

To perform Superb-seq, we performed IST reactions on thawed aliquots of fixed sample pools, followed by combinatorial barcoding of single-cell RNA with an off-the-shelf SPLiT-seq kit (Fig. 3A, Supp. Figs. 9B, 10). To control for background T7 transcription (Supp. Fig. 4B,C) in downstream gene quantification, we applied the same IST treatment to both unedited and edited sample pools. We generated two Superb-seq libraries, a 500 cell library and a 10,000 (10k) cell library (Supp. Fig. 9C,D). T7 cDNA was detected in both cDNA libraries (Supp. Fig. 9E), indicating successful capture of T7 fragments. To assess library quality, we performed deep paired-end sequencing (200k reads/cell) of the 500 cell library, and used Split-pipe^51^ to align reads to the human reference genome (hg38), correct cell barcodes for sequencing errors, call high-quality cells, and perform initial read-gene assignments. In total, there were 126 million high-quality reads with a mapping rate of 66.7%, and 583 high-quality cells with 217k reads/cell (Supp. Fig. 9F–I). Based on this high sequencing performance, we then performed paired-end sequencing and Split-pipe analysis of the 10k cell library at half the sequencing depth (100k reads/cell). We observed 937 million high-quality reads with a mapping rate of 78.8%, and 9,500 high-quality cells with 98.6k reads/cell (Fig. 3B, Supp. Fig. 9F–I). These results demonstrate that the Superb-seq workflow generates high-quality combinatorial scRNA-seq reads.

### Sheriff software distinguishes T7 and transcriptome reads from joint Superb-seq data

To quantify single-cell Cas9 edits and transcriptomes from Superb-seq reads, we developed a custom software suite called **“Sheriff”**. Sheriff processes Split-pipe read alignments to quantify both edit sites and unique molecular identifier (UMI) counts per gene (Fig. 3A, Supp. Fig. 11). Sheriff detects high-quality T7 reads by the presence of a T7 barcode sequence in the 5’ soft-clipped portion of mapped reads, which is encoded in the inserted donor DNA (Fig. 1D, Supp. Fig. 11C). This soft-clipped barcode feature distinguishes edit-marking T7 reads from background T7 reads and endogenous RNA reads. To reduce false-positive T7 read calls, Sheriff accounts for other sources of clipped reads and barcode-like sequences (e.g. sequencing errors, template switch oligo sequence). With this process, Sheriff identified deep T7 read pileups precisely at all seven expected on-target edit sites (Supp. Fig. 12A).

We estimated the performance of T7 read calling by Sheriff using unedited sample reads as a ground truth negative set, and approximated a positive set (i.e. pure T7 reads) by filtering for reads within ± 100 bp of the seven on-target edit sites. The sensitivity, or classification of T7 reads at on-target sites was 95% (Supp. Fig. 12B), although this is likely an underestimate due to confounding mRNA reads. The specificity, or classification of non-T7 reads, was > 99% with a false discovery rate of < 0.2% (i.e. non-T7 reads classified as T7 reads) (Supp. Fig. 12C,D). These results indicate that Sheriff accurately distinguishes T7 reads from non-T7 reads.

Next, Sheriff generates high-quality single-cell transcriptome counts, removing confounding non-barcoded T7 reads ± 1 kilobase from barcoded T7 reads (Supp. Fig. 8B,C, Supp. Fig. 11E). Across samples, Sheriff identified > 45k mean UMIs/cell and > 7.8k mean genes/cell in the 500 cell transcriptomes, and > 27k mean UMIs/cell and > 6.8 mean genes/cell in the 10k cell transcriptomes (Fig. 3B, Supp. Fig. 13). Standard single-cell analysis and clustering of the 10k cell transcriptomes with Scanpy^52^ and Cytocipher^53^ identified 11 clusters of cells with distinct transcriptional profiles, with no distinct clustering observed between unedited and edit sample pools (Fig. 3C,D, Supp. Fig. 14). This suggests that Cas9 edits did not result in overt cell state changes, consistent with previous single-cell CRISPR screens^9,54^. These results established that our Superb-seq workflow generates high-quality single-cell transcriptome data.

### Superb-seq quantifies genome edit alleles at single-cell and base pair resolution

Next, Sheriff software identifies high-confidence Cas9 edit sites by aggregating T7 reads across cells and locating edit-specific features such as divergent opposite-strand reads from bi-directional T7 promoter insertions (Supp. Fig. 8B,C, Supp. Fig. 11B). Sheriff analysis of the 10k cell library identified the on-target edit sites of all seven guide RNAs, and 36 off-target edit sites in 6,230 edited cells (Fig. 3E, Supp. Material 1, Supp. Table 3). We observed high T7 UMI counts at *CHD3* (6.2k UMIs), CHD4 (11.6k UMIs) and *SMARCA4* (1.5k UMIs) that were consistent with our RT-PCR results (Supp. Fig. 7, Supp. Table 3). Superb-seq also detected Cas9 edits where RT-qPCR was insensitive, with 548 T7 UMIs detected at *ARID1A.* Furthermore, Sheriff resolved Cas9 edit sites at base-pair resolution, positioning each on-target edit 0–3 bp from the expected Cas9 cut position (Supp. Fig. 12E, Supp. Tables 1, 3).

To determine the effectiveness of single-cell Cas9 edit capture by Sheriff, we compared edit site detection and edited cell frequencies between the smaller 500 cell library with higher sequencing depth (126 million total reads, 200k reads/cell) and the larger 10k cell library with lower sequencing depth (937 million reads, 100k reads/cell (Supp. Fig. 15A–G). The two libraries detected similar proportions of edited cells from a given sample pool (~70% of cells within edited sample pools, Supp. Fig. 15B,E), with negligible false positive T7 UMIs called in the unedited sample (Supp. Fig. 15B,E), indicating that the 10k cell library accurately captured the majority of each sample’s edited cells despite half the sequencing depth. More edit sites were detected in the 10k cell library than the 500 cell library (43 vs. 30, Supp. Fig. 15C) because the larger library could capture rare edit sites affecting few cells (Supp. Fig. 15C). The 13 edit sites that were only detected in the 10k library occurred in as few as three cells, and were therefore not expected to be present in the 500 cell library (mean expectation of 0.5 edited cells, range 0.2–1.4, Supp. Fig. 15G). Among the 30 edit sites detected in both libraries, 24/30 were mapped to the exact same edit site, with all but one mapping within 2 bp between libraries (Supp. Fig. 15F). These results establish that Sheriff performs reproducible single-cell edit site characterization, and that scaling to larger libraries can increase the sensitivity of Superb-seq for rare edit sites.

When we examined the barcoded T7 read alignments within individual cells, we observed variation in alignment sequence and position that indicated more than one copy of the genome site had been edited by Cas9 in the same cell (Supp. Fig. 16). Therefore, we automated Sheriff to estimate the number of unique Cas9 edit copies per site per cell, which we term the “edit allele dosage” (Methods, Supp. Fig. 11F). To assess the rigor of edit allele quantification, we used edit allele counts from the lower-depth 10k cell library to estimate expected outcomes for the higher-depth 500 cell library, and compared expectations to observed outcomes. We found a very high correlation between expected and observed edited cell counts (Pearson’s R = 0.985, two-sided p = 6.9×10^−33^) and edit allele counts (Pearson’s R = 0.976, two-sided p = 6.8×10^−29^) in the 500 cell library (Supp. Fig. 15G,H), indicating that the 10k cell library effectively captured edited cells and edit alleles across different edit sites, despite half the sequencing depth. At the *CHD4* on-target edit site, however, more edited cells and alleles were observed in the 500 cell library than expected. This suggests undercounting of *CHD4* edit alleles in the 10k cell library, possibly resulting from a high rate of homozygous editing (Fig. 1F). These results establish that Sheriff effectively quantifies single-cell Cas9 edit allele dosage in most cells.

### Superb-seq discovers Cas9 off-target events at single-cell resolution

**Figure 4.**
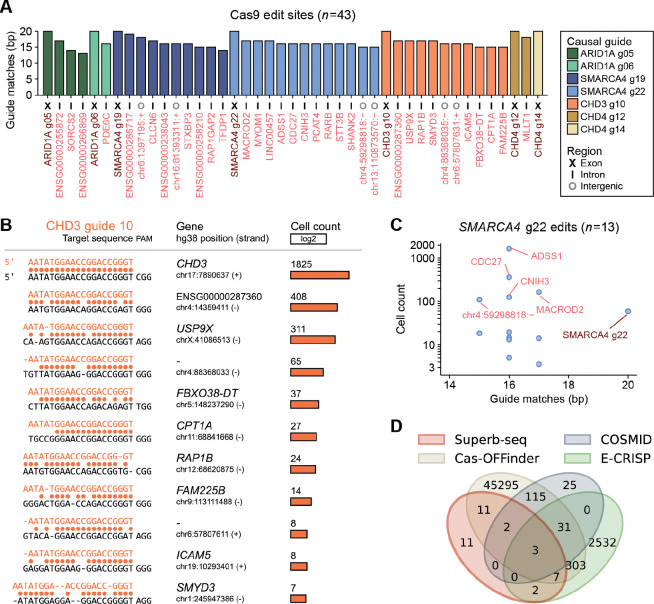
Superb-seq characterizes *de novo* off-target edit sequences. (**A**) Genome regions and guide sequence similarity of on-target and off-target edit sites identified by Sheriff. Name labels are colored by on-target (dark red) or off-target (light red). Bars are colored by causal guide RNA. Intersection of exon (“X”), intron (“I”), or intergenic sequence (“O”) is indicated. (**B**) Global alignments of guide and genome sequences for 11 edit sites associated with *CHD3* guide 10, and corresponding total cell counts. (**C**) Frequency 12 off-target sites of *SMARCA4* guide 22 (light red), compared to the on-target site (dark red). (**D**) Intersection of Superb-seq detected off-target sites with predicted sites from *in silico* tools Cas-OFFinder, COSMID, and E-CRISP, across the seven guides.

We discovered 36 off-target edit sites that were generated by the seven guide RNAs in the 10k cell library (Fig. 3E). These off-targets occurred across 18 chromosomes and varied widely in frequency, from three cells to 1768 cells (0.03%–18.6% of cells within edited sample pools). All off-target sites were within non-coding sequences, and > 80% (30/36) were within introns (Fig. 4A). Since non-coding sequences can effect gene regulation, it is challenging to anticipate the downstream effects of these Cas9 off-targets based on edit location alone.

Since Superb-seq captures edit events instead of delivered guides, we analyzed two features of the detected off-target sites to verify the causal guide of each: (1) sequence similarity to a particular guide, and (2) co-occurrence of off-target edit alleles with those of a particular on-target site (Methods, Supp. Figs. 17, 18). In our dataset, each off-target site harbored an adjacent PAM sequence with high sequence similarity to one guide sequence (Fig. 4A,B, Supp. Figs. 12F, 18, Supp. Table 3), indicating identification of causal guides with high confidence. We observed an average of six off-target edit sites per guide, with a maximum of 13 edit sites for *SMARCA4* guide 22. Only *CHD4* guide 14 had no detectable off-target edits (Fig. 4A). Despite unique mapping to a single genome site and high predicted specificity (Supp. Table 1), > 85% (6/7) of guide RNAs generated off-target edits.

Most off-target edit sites were detected at a lower frequency than the corresponding on-target site (Fig. 4B, Supp. Figs. 18, 19). However, *SMARCA4* guide 22 generated five off-target edit sites that were detected at a 1.8–34 fold higher frequency than at *SMARCA4* (Fig. 4C). The most frequent off-target occurred in an intron of *ADSS1* in 1768 cells, compared to 52 cells for the *SMARCA4* on-target site. The *ADSS1* off-target sequence had 16/20 base matches to *SMARCA4* guide 22, all of which occurred in the non-seed region (Fig. 4C, Supp. Fig. 18D, Supp. Table 3).

Notably, many off-target sequences, even high-frequency sites, had alignments suggestive of bulge formation or non-canonical base pairing to the imputed guide (Fig. 4B, Supp. Figs. 18, 20). Many mismatches occurred within the “seed” region that strongly influences Cas9 activity^55^ (Supp. Fig. 20), indicating that Cas9 can tolerate a variety of both seed and non-seed guide mismatches.

We tested whether the off-target sites detected by Superb-seq could be predicted *in silico* using Cas-OFFinder^56^, E-CRISP^57^, and COSMID^58^. Each tool generated a large number of predicted off-target sites, with poor overlap with the observed off-targets (Fig. 4D). Cas-OFFinder identified the most Superb-seq off-targets (64%, 23/36), but at the cost of very low specificity (0.05%, 23/45,767, Fig. 4D). These results indicate the difficulty of predicting these Cas9 off-target sites *in silico*, consistent with previous work^18^, and the value of *de novo* edit site detection by experimental methods like Superb-seq.

### Superb-seq associates on- and off-target genome edits to differential gene expression

**Figure 5.**
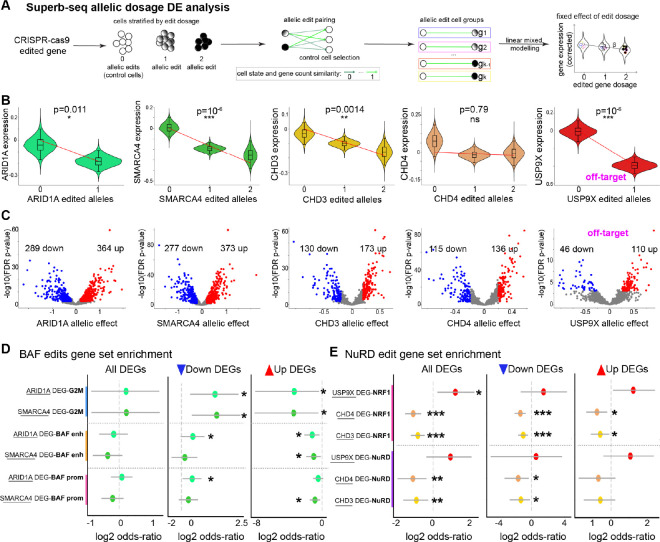
Superb-seq detects differentially expressed genes associated with both on- and off-target edit events. (**A**) Overview of the linear mixed model used to call differentially expressed genes (DEGs) associated with edit allelic dosage while accounting for cell state and gene detection rate per cell. (**B**) Violin plots of corrected gene expression estimates from n=10,000 bootstrap iterations stratified by edit allele dosage for each edit site. Overlaid box-plots show the interquartile range with the whiskers indicating the full data range (minimum and maximum). Lines indicate the effect on expression of edit allele dosage estimated by the linear mixed model. P-values are from performing a two-sided Wald test using the linear mixed model followed by Benjamini-Hochberg p-value correction. (**C**) Volcano plots of DEGs associated with edits. Edit allele effect sizes are on the x-axes and −log10 Benjamini-Hochberg corrected p-values on the y-axes. (**D**) Functional enrichment analysis of BAF perturbations (*ARID1A* and *SMARCA4* edits), depicted as log2 odds ratios with two-sided 95% confidence intervals. Y-axis indicates the edit-associated DEG set and query gene set being compared. Vertical dotted lines indicate the null expectations. Query gene sets were hallmark G2M checkpoint genes (G2M), genes with BAF-engaged enhancers (BAF enh), or genes with BAF-engaged promoters (BAF prom). (**E**) Functional enrichment analysis of NuRD perturbations (*CHD3* and *CHD4* edits). Query gene sets were genes with NRF1-engaged promoters (NRF1), or genes with NuRD engagement (NuRD). For panels **D,E** *p < 0.05, **p < 0.01, ***p < 0.001 by two-sided Fisher’s exact test with Benjamini-Hochberg p-value correction.

Since prior Perturb-seq analysis methods handle single-cell guide information^3,54^ but not single-cell edit allele information, we developed a novel linear mixed modeling approach to test for associations between edit allele dosage and differential gene expression in Superb-seq data (Methods, Fig. 5A). We first applied this approach to test whether on-target Cas9 edits affected the expression of the four targeted chromatin remodeler genes. On-target edits to *ARID1A*, *SMARCA4*, and *CHD3* resulted in significantly decreased gene expression as edit dosage increased (FDR p < 0.05, two-sided Wald test, Fig. 5B), consistent with edit-induced NMD of these genes observed by RT-qPCR (Supp. Fig. 2D). The change in expression of *CHD4* was not significant (FDR p = 0.79, two-sided Wald test), consistent with modest *CHD4* decrease by RT-qPCR and less efficient NMD (Supp. Fig. 2D). However, another plausible factor is underestimation of *CHD4* edit allele dosage (Supp. Fig. 15G,H). These results demonstrate that Superb-seq detects the expected effects of on-target Cas9 editing to target gene expression.

We next tested for gene expression effects associated with four frequent off-target edits that we detected within K562-expressed genes *CDC27*, *CNIH3*, *FBXO38-DT*, and *USP9X* (Supp. Figs. 14F, 19). Off-target *USP9X* editing significantly decreased *USP9X* expression (FDR p = 10^−6^, two-sided Wald test), a deubiquitinase with diverse cellular roles^59^ (Fig. 5B, Supp. Fig. 21A–C, Supp. Table 4). The *USP9X* off-target was generated by the *CHD3* guide RNA (Fig. 4B) and occurs 400 bp downstream of the transcription start site within the first intron. The edit site is 2 bp from a candidate enhancer intersecting a REMAP^60^ cis-regulatory module and intersects a binding motif and K562 ChIP-seq peak of the transcription factor SP1^61^ (Supp. Fig. 22), knockdown of which has been shown to reduce *USP9X* expression^62^. Therefore, *USP9X* expression is likely reduced because the off-target edit disrupts SP1 activity at a cis-regulatory element for this gene. These results show that in addition to the *de novo* detection of off-target Cas9 edit sites, Superb-seq enables functional effects associated with off-target edits to be characterized, including those that affect non-coding sequences.

We then tested for associations between Cas9 edits and additional downstream gene expression effects. Hundreds of differentially expressed genes (DEGs) were associated with Cas9 editing at all four on-target genes and at off-target *USP9X* (Fig. 5C, Supp. Table 4). To determine if the identified DEGs were consistent with known downstream functions of the corresponding edited genes, we performed functional enrichment analyses. We tested whether the sets of DEGs associated with BAF editing (*ARID1A/SMARCA4*) and NuRD editing (*CHD3*/*CHD4*) were enriched in genes regulated by BAF and NuRD, respectively, determined by ChIP-seq (Methods, Supp. Table 5). Since BAF regulates cell proliferation^63,64^, we also tested for enrichment with a set of G2/M cell cycle checkpoint genes. We observed significant intersections of G2/M genes and BAF-targeted genes among BAF edit DEGs, and likewise for NuRD-targeted genes among NuRD edit DEGs, indicating consistency between observed and expected downstream transcriptome effects (Fig. 5D–E, Supp. Tables 6, 7). The BAF complex promotes gene expression^65^, therefore BAF disruption by Cas9 edits to *ARID1A* and *SMARCA4* are expected to decrease the expression of known BAF-targeted genes. Indeed, there was a significant number of genes with BAF binding at an enhancer or promoter that were downregulated in response to BAF editing (Fig. 5D). Likewise G2/M checkpoint genes were also significantly downregulated in response to BAF editing (Fig. 5D). In contrast to BAF, the NuRD complex performs transcriptional repression^66^, therefore *CHD3* and *CHD4* edits are expected to increase the expression of NuRD-targeted genes. In-line with this expectation, genes with NuRD promoter binding were depleted in down-regulated DEGs associated with NuRD editing (Fig. 5E). These results indicate that Superb-seq captures the downstream transcriptomic effects of individual Cas9 edits.

Lastly we examined the downstream effects of the off-target edit at *USP9X*. Since *USP9X* regulates protein abundance in myriad cellular processes^59^, the downstream gene expression effects are difficult to anticipate. However, one of the DEGs specific to *USP9X* editing was *NRF1* (Supp. Table 4), a transcription factor that regulates proteasome machinery including *USP9X^67^*. We observed that the set of *USP9X*-specific DEGs (i.e. DEGs absent in *CHD3* edit DEGs) contained a significant number of NRF1-targeted genes determined by ChIP-seq (FDR p = 0.014, OR = 2.4, two-sided Fisher’s exact test, Fig. 5E, Supp. Table 7), with 63% (24/38) of DEGs regulated by NRF1 (Supp. Table 6). In contrast, NRF1 target genes were under-represented among *CHD3* and *CHD4* edit DEGs, suggesting that the NRF1 effect was specific to *USP9X* editing (Fig. 5E). In summary, our enrichment analysis revealed that Superb-seq captures downstream gene expression effects of off-target Cas9 edit events.

## Discussion

Superb-seq achieves high-throughput single-cell quantification of Cas9 genome edit events, a longstanding technical gap in current CRISPR methods that has impeded Cas9 applications in functional genomics and gene therapy. Although methods like GUIDE-seq, DISCOVER-seq and Tracking-seq capture off-target edit sites^21,24,26^, these are low throughput bulk assays that do not measure associated gene expression effects and may miss rare edit events occurring in small numbers of cells. Superb-seq achieves capture of Cas9 edits in thousands of single cells through T7 *in situ* transcription (IST), which has recently been applied to image-based genome perturbation^31,32^, multiplexed screening of prime edit determinants^30^, and structural variant effects^68^. To analyze single-cell T7 transcripts, these methods used lower-throughput *in situ* sequencing, or integrated datasets from tandem bulk and single-cell RNA-seq experiments. Superb-seq achieves combinatorial scRNA-seq of T7 and endogenous transcripts in one experiment through careful selection of a suitable T7 promoter, optimization of IST conditions, and the use of blended-primer SPLiT-seq barcoding. These strategies of Superb-seq will be generally useful for single-cell sequencing of *in situ* transcripts in future applications.

Application of Superb-seq to 10,000 cells identified 36 off-target edit sites, one of which occurred in 34 times more cells than the corresponding on-target edit, even though only 16/20 bp of the guide spacer sequence matched the off-target sequence. The frequent edits at the off-target site could potentially be due to more accessible chromatin or gRNA:DNA interactions that promote more efficient editing^69^. Alternatively, mutations within the K562 genome may have reduced the number of mismatches between the spacer and the off-target sequence^27^. Regardless of the mechanism, when guide presence is used as a proxy for on-target editing in the absence of direct edit capture, off-target events such as these may confound downstream analyses.

While all of the off-target sites had clear sequence similarity to the guide spacer sequence, many were not predicted by *in silico* tools, including one off-target edit that altered *USP9X* expression, illustrating the risk that unpredictable off-target edits, even to non-coding sequences, may cause undesirable cellular consequences. Off-targets are of particular concern for gene therapies, where possible side effects have included oncogene activation and cancer^70^. Superb-seq has the potential to identify off-target events and their effects on gene expression before candidate CRISPR therapies enter patients.

In the 10,000 cell library, we detected rare edit sites that occurred in as few as three cells. By identifying rare edits and quantifying their frequency, Superb-seq has the potential to mitigate rare but dangerous off-target edits in CRISPR therapies. On the other hand, many of the off-targets identified did not affect gene expression. The ability to distinguish between off-targets that do or do not affect gene expression will be useful for discriminating candidate therapeutic guides with benign or risky off-target perturbations. Combinatorial barcoding could be effectively scaled to perform larger Superb-seq screens with power to assess the gene expression effects of less frequent off-target events.

A limitation of Superb-seq is that Cas9 edits can only be detected when the donor sequence is inserted, so edit capture may be biased toward sites with efficient donor insertions. Sites with lower rates of T7 transcription, or cells with lower sequencing depth, could also cause undetected edits. Nonetheless, Superb-seq detects many edited alleles with high confidence, which enables differential expression analysis by comparing cells with specific edit allele dosages to control cells.

Overall, Superb-seq achieves a major step forward in the field of genome editing by enabling genome-wide measurement of Cas9 edits and gene expression in thousands of single cells. Superb-seq can be performed with standard laboratory equipment, off-the-shelf supplies, and does not require viral vectors. We envision the method will be broadly accessible to other researchers and will increase access to CRISPR screens in primary cells and cell types that are difficult to transduce. Most importantly, Superb-seq could be used to screen pre-clinical guide RNA candidates and avert deleterious off-target effects of therapeutic Cas9 editing.

## Methods

### Oligonucleotides

Sequences of all donor DNA, guide RNA, and RT-qPCR primer oligonucleotides are listed in Supp. Table 1. All oligonucleotides were synthesized by Integrated DNA Technologies (IDT).

### Human subjects

Primary human T cells were isolated from de-identified healthy donor blood from the San Diego Blood Bank (San Diego, CA). An institutional review board (IRB) at the Salk Institute for Biological Studies determined that this work did not meet the 45 CFR 46 definition of human subjects research and was therefore exempt from IRB review and informed consent requirements.

### Cell culture

All cells were incubated at 37°C, 5% CO_2_ and maintained at cell densities of 0.1–0.5 million (M) cells/mL (K562 cells) or 0.2–1.5 M cells/mL (all other cell types). Human cell lines K562, Jurkat (clone E6–1), and GM12878 were cultured in RPMI-1640 (Gibco #11875–093) supplemented with 10% heat-inactivated fetal bovine serum (FBS, GeminiBio #100–500), 100 U/mL penicillin, and 100 μg/mL streptomycin (Gibco #15140–122).

For primary human T-cell culture, peripheral blood mononuclear cells were isolated from healthy donor buffy coats by centrifugation on Ficoll-Paque Plus (Cytiva #17144002) or Lymphoprep (StemCell Technologies #07851) density gradient media, and mononuclear fractions were cryopreserved in heat-inactivated FBS with 10% DMSO (Sigma #D8418). Frozen mononuclear cells were thawed and cultured in IMDM with 25 mM HEPES (Gibco #12440–053), supplemented with 10% heat-inactivated FBS, 50 U/mL recombinant human IL-2 (Miltenyi Biotec #130–097-746), 100 U/mL penicillin, and 100 μg/mL streptomycin.

To activate T cells, 6-well plates were coated with anti-human CD3 antibody (clone OKT3, BioLegend # 317326) by adding 1 mL per well of 1 μg/mL anti-CD3 in Dulbecco’s phosphate-buffered saline without calcium and magnesium (DPBS, Sigma #D8537–500ML), incubating at room temperature for 1 hour, and aspirating. Frozen mononuclear cells were thawed, rested for least 1 hour at 37°C, and seeded in coated plates at 1 M cells/mL in the above T-cell medium supplemented with 1 μg/mL anti-human CD28 antibody (clone CD28.2, BioLegend #302934), then expanded in T-cell medium without anti-CD28.

### Donor DNA design

The 34 base pair (bp) Superb-seq donor DNA (donor 02) was generated as follows. An optimized phage T7 promoter sequence was obtained from the Cel-seq+ method^41^. This sequence is composed of a 20 bp core T7 promoter sequence^71^ (5’-TAATACGACTCACTATAGGG-3’) and flanking 5’ and 3’ sequences that maximize promoter activity^41,72^. To this sequence, we added 2 bp terminal sequences, a 5’ phosphate and two 3’ phosphorothioate modified bases used in the GUIDE-seq donor^73^ (Fig. 1B, Supp. Fig. 1C, Supp. Table 1).

We designed 26 additional donor constructs by using alternative T7 and SP6 promoter variants and flanking sequences^38–41^, removing flanking sequence, or incorporating dual promoters and alternative polyA or tracrRNA capture sequences^6^(Supp. Table 1). To maximize gene-disrupting frameshifts upon donor insertion in coding sequence, all donors were made to length *3n+1* to yield +1 shifts upon precise insertion and +2 shifts upon common 1-bp templated insertion due to staggered Cas9 cleavage^74–79^. All donor sequences were ordered with the above GUIDE-seq base modifications as pre-duplexed lyophilized oligonucleotides from IDT, reconstituted to 100 μM in electroporation buffer (MaxCyte #EPB-1), and stored at −20°C.

### Cas9 edit labeling with donor DNA encoding the T7 promoter

To label Cas9 edits with T7 promoter sequences, we performed homology-free knock-in. Live cells were treated with donor 02 DNA and CRISPR-Cas9 ribonucleoprotein (RNP) by electroporation^33^. Single guide RNAs (sgRNAs) were assembled with 20 bp guide (protospacer) sequences, a modified “tracrV2” scaffold sequence^80^, and the common 2’ O-methyl 3’ phosphorothioate modifications on terminal bases^81^. Guides with specificity scores > 0.45 and efficiency scores > 0.3 were selected using GuideScan^82^ or GuideScan2^83^. Guide sequences targeting *TRAC* and *B2M* were previously reported^8,84^. All sgRNAs were synthesized by IDT, reconstituted to 125 μM (4 μg/μL) in electroporation buffer (MaxCyte #EPB-1), and stored at −20°C. All sgRNA sequences are listed in Supp. Table 1.

RNP was prepared by mixing 1 μL of 10 μg/μL purified *S. pyogenes* Cas9 protein (IDT #1081059) and 1 μL of 4 μg/μL of sgRNA (2:1 sgRNA:Cas9 molar ratio), incubating at room temperature for 15 minutes, and storing on ice until electroporation. Cells were harvested, washed once with 5–10 mL electroporation buffer, and resuspended in electroporation buffer to 100–125 M cells/mL. Per 25 μL electroporation, 5 μL of transfection mix was prepared with 2 μL RNP, 1 μL of 100 μM donor DNA, 1 μL of 100 μM Cas9 enhancer (IDT #1075916), and 1 μL electroporation buffer. Next, 20 μL of cells (2–2.5 M) was mixed with 5 μL of transfection mix, transferred to an OC-25×3 process assembly (MaxCyte #SOC-25×3), and immediately transfected with an ExPERT ATx electroporation system (MaxCyte) using cell type-specific instrument protocols provided by MaxCyte (Supp. Table 2). Final electroporation concentrations were 80–100 M cells/mL, 2.5 μM Cas9, 5 μM sgRNA, 4 μM donor DNA, and 4 μM Cas9 enhancer. After electroporation, cells were immediately seeded at 0.5 M cells/mL (K562) or 1 M cells/mL (all other cell types) in a 6-well plate with warm cell culture medium, expanded, and cryopreserved.

### TIDE quantification of T7 promoter-labeled Cas9 edits

On day 3 post-electroporation, genomic DNA (gDNA) was extracted from edited and control cells using a Quick-DNA Microprep kit (Zymo Research #D3020) and eluted in nuclease-free water. PCR amplicons were generated with primer pairs designed by Primer-BLAST^85^ with the following custom parameters: size 900–1200 bp, melting temperature 53–55–57°C (min-opt-max), database genomes for selected eukaryotes, organism homo sapiens, max size 30, 40–60% GC, GC clamp of 1, max poly-X of 4, max GC in 3’ end of 4, human repeat filter, and low complexity filter on. Primer pairs were designed to amplify ~1 kilobase genome regions from ~150 bp upstream of edit sites to ~850 bp downstream. Per 20 μL PCR reaction, ~50 ng gDNA was combined with 0.5 μM of each primer, nuclease-free water, and either Phusion Plus (Thermo Scientific #F631S) or Phusion High-Fidelity (Thermo Scientific #F531S) master mix. PCR reactions were run on a SimpliAmp thermal cycler (Applied Biosystems) with the following protocol: 98°C for 2 minutes, 35 cycles of [98°C for 10 seconds, 60°C for 10 seconds, 72°C for 30 seconds], 72°C for 5 minutes, and then 10°C hold (ramp rate 2°C/second). PCR reactions were purified by ExoSAP-IT Express reagent (Applied Biosystems #75001.200.UL) or by NucleoSpin PCR cleanup kit (Takara #740609.25). Sanger sequencing of PCR amplicons was performed by Eton Bioscience (San Diego, CA) or Genewiz (San Diego, CA), and sequence traces (AB1 files) were analyzed by TIDE (tracking indels by decomposition)^42^ using the batch analysis web tool (https://apps.datacurators.nl/tide-batch/) with the following parameters: left boundary 50, indel size range 50 (−50 to +50), and default decomposition window and p-value threshold (0.001). All primer sequences are listed in Supp. Table 1.

### Flow cytometry of target protein knockdown

On day 7 post-electroporation, unedited and donor-labeled GM12878 cells were harvested into a 96-well round-bottom plate, washed twice with 200 μL cell staining buffer (BioLegend #420201), spun down at 200 x *g* for 2 minutes, flick-decanted, gently vortexed, and stained with 50 μL of a 1:200 dilution of PE anti-human B2M antibody (clone 2M2, BioLegend #316305) in cell staining buffer. An isotype control was prepared by staining unedited cells with an equivalent concentration of diluted PE mouse IgG1 κ antibody (clone MOPC-21, BioLegend #400114). Cells were stained in the dark for 15 minutes at room temperature, washed twice with 150–200 μL cell stain buffer, and resuspended in 150 μL cell stain buffer. Forward/side scatter and PE fluorescence intensity were acquired using a BD FACSymphony A3 cell analyzer and HTS attachment. Live (FSC-A/SSC-A) and singlet (FSC-A/FSC-H) events were gated and plotted using R packages flowCore^86^ and ggcyto^87^.

### Nuclei isolation

Nuclei buffers were prepared in an RNase-free environment by the Omni-ATAC protocol^88^. A basal buffer of 10 mM Trizma HCl (Sigma #T2194–100ML), 10 mM NaCl (Sigma #S5150–1L), 3 mM MgCl_2_ (Sigma #M1028–100ML), 0.1% Tween-20 (Sigma #11332465001), and 48.75 mL nuclease-free water (Corning #46000CI) was prepared and stored at 4°C. For each nuclei isolation, fresh lysis and wash buffers were prepared. Lysis buffer was prepared with basal buffer supplemented with 0.1% IGEPAL CA-630 (Sigma #I8896–50ML), 0.01% digitonin (Invitrogen #BN2006) (stock solution dissolved at 65°C), and 1 U/μL RiboLock RNase inhibitor (Thermo Scientific #EO0382). Wash buffer was prepared with basal buffer supplemented with 1 U/μL RiboLock. Fresh buffers were kept on ice until use.

To isolate human nuclei, cells were harvested, washed once with DPBS, filtered through 40 μm cell strainer (Fisherbrand #22363547), and counted. All steps and spins were performed on ice or 4°C with ice-cold buffers and low-bind tubes. To lyse cells, 2 M cells were lysed by gently resuspending in 45 μL lysis buffer (pipetting up-down exactly 3 times) and incubating on ice for 3 minutes. Nuclei were immediately washed twice by gently resuspending in 250 μL wash buffer, spinning down at 500 x *g* for 5 minutes, manually aspirating, resuspending in 100 μL wash buffer, filtering through a 40 μm Flowmi cell strainer (SP Bel-Art #H13680–0040), spinning down, aspirating, and resuspending in 35 μL wash buffer. Nuclei were counted and imaged by TC 20 automated cell counter (Bio-Rad). To generate larger quantities of nuclei for formaldehyde fixation, cell and reagent amounts were scaled proportionally. Aliquots of 0.2–0.5 M nuclei were stored at −80°C.

### Cell and nuclei fixation

Cells or freshly isolated nuclei were fixed in an RNase-free environment using Evercode Fixation kits (Parse Biosciences #ECF2001, #ECF2003, #ECF2101, #ECF2103) according to kit protocol. Per fixation, 3 M input cells or nuclei were used. Fixation yield was ~50% (1.5 M fixed singlets). Aliquots of 0.5 M fixed cells or nuclei were stored at −80°C.

### T7 *in vitro* transcription

*In vitro* transcription (IVT) and *in situ* transcription (IST) was performed in an RNase-free environment using the HiScribe T7 Quick RNA synthesis kit (New England BioLabs #E2050S). For IVT on purified gDNA, gDNA was extracted from donor-edited and control cells using a Quick-DNA Microprep Plus kit (Zymo Research #D4074) and eluted in nuclease-free water. Each 20-μL IVT reaction was prepared with 0.5–1 μg gDNA, 10 μL of 2X HiScribe NTP/cofactor buffer mix (10 mM NTP final), 2 μL of 10X HiScribe T7 RNA polymerase (or water for “no polymerase” controls), and nuclease-free water, and incubated at 37°C for 2 hour. For IST on unfixed nuclei, 40 μL reactions were prepared by gently mixing 16 μL of freshly isolated nuclei (~100,000 nuclei) with a master mix of 20 μL of 2X HiScribe NTP buffer mix and 4 μL of 10X HiScribe T7 polymerase (or water), and incubated at 37°C for 2 hours.

### T7 *in situ* transcription

Prior *in situ* transcription methods have primarily used methanol-fixed cells^29,30^. In contrast, SPLiT-seq uses paraformaldehyde (PFA) fixation^46^. Therefore, we evaluated whether T7 IST reactions are functional in PFA-fixed cells by fixing cells containing promoter-labeled edits and performing immediate IST. We observed both T7 RNA at both edit sites and endogenous promoter-like sites (Supp. Fig. 5E, Supplementary Note 3), indicating that the T7 RNA polymerase was active in PFA-fixed cells, consistent with a recent report^31^. IST on fixed and unfixed nuclei from the same nuclei isolation suggested that typical IVT/IST reaction conditions (37°C, 2 hours) were sub-optimal (Supp. Fig. 5G,H).

We systematically optimized IST conditions for PFA-fixed cells and identified a specific temperature, time, and NTP concentration (40°C, 24 hours, 10 mM) that greatly improved reaction efficiency (Supp. Fig. 6A–C). Under these optimized conditions, levels of T7 RNA surpassed *RPL24* mRNA with minimal increase in background signal (Fig. 2H, Supp. Fig. 6C). Importantly, these levels of T7 RNA were observed in the cell pellet fractions of T7 IST reactions, suggesting that abundant T7 RNA is retained within fixed cells. To determine whether unfixed T7 RNA is resistant to wash out, we performed IST on fixed cells and washed cell pellets, and then measured T7 RNA levels in wash supernatant (ambient) and post-wash pellet (*in situ*) fractions. T7 RNA was detected in the ambient fraction, indicating some washout. However, the *in situ* fraction maintained a level of T7 RNA equivalent to *RPL24* (Supp. Fig. 6D). These results established that unfixed T7 transcripts are retained by fixed cells and suggested suitability for single-cell combinatorial indexing.

For IST on fixed cells (or nuclei), the optimal protocol was to gently add 15 μL of fixed cells (~100,000 cells) in kit-provided storage buffer to a 25 μL master mix of 20 μL of 2X HiScribe NTP buffer mix (10 mM NTP final), 4 μL of 10X HiScribe T7 polymerase, and 1 μL of 40 U/μL RiboLock (1 U/μL final), and incubate at 40°C overnight for up to 24 hours. This method was used for all Superb-seq samples. During IST optimization, we tested additional methods including the exchange of fixed cell storage buffer with Omni-ATAC buffer (caused cell clumping), increasing NTP concentration to 20 mM (decreased IST efficiency), and incubating at 37°C or 42°C for 0.5–4 hours (less efficient and/or less specific) (Supp. Fig. 6).

### Total RNA purification

Total RNA was extracted from T7 IVT and IST reactions by TRIzol and column purification. TRIzol LS reagent (Ambion #10296010) was added at a 3:1 ratio (120 μL TRIzol LS per 40 μL T7 reaction). To extract total RNA from IST pellet and supernatant fractions separately, IST reactions were spun down in a mini centrifuge (~2,000 × *g* for 1 minute) and split into pellet and supernatant fractions. Pellet fractions were treated with 160 μL TRIzol reagent (Ambion #15596026), and 40 μL supernatant fractions were treated with 120 μL TRIzol LS. Total RNA was purified from TRIzol lysates using a Direct-zol RNA microprep kit (Zymo Research #R2060), with on-column DNase, and eluted in nuclease-free water. For sequencing, an additional 20 μL off-column DNase treatment was performed by mixing 10 μL eluted RNA, 1 μL of 2 U/μL Turbo DNase (Invitrogen #AM2238), 2 μL of 10X Turbo DNase buffer, 7 μL nuclease-free water and incubating at 37°C for 30 minutes before purifying by RNA Clean and Concentrator kit (Zymo Research #R1013) and eluting in nuclease-free water. Purified RNA was quantified by UV spectroscopy (A260/A280) using a DeNovix DS-11+ spectrophotometer, or by calibrated fluorometry using a Qubit 3 fluorometer (Invitrogen) and RNA HS kit (Invitrogen #Q32852) or RNA BR kit (Invitrogen # Q10210). For qPCR, purified total RNA was stored −20°C for up to one week. For sequencing, purified total RNA was stored at −80°C.

### Reverse transcription quantitative PCR (RT-qPCR)

For RT-qPCR of T7 RNA, custom primer pairs were designed using Primer-BLAST^85^ with the following custom parameters: size 80–120 bp, melting temperature 58–60–62°C (min-opt-max), max T_m_ difference of 2, database genomes for selected eukaryotes, organism homo sapiens, max size 30, 40–60% GC, GC clamp of 1, max poly-X of 4, max GC in 3’ end of 4, human repeat filter, and low complexity filter on. To measure on-target T7 RNA levels, primer pairs were designed to target flanking regions of donor-labeled edit sites. Control primer pairs were designed for several intergenic sequences on different chromosomes, including two “genomic safe harbor” gene deserts (GSH1, GHS2)^49^. Additional primer pairs were designed to flank three sites in the human genome with high sequence similarity to the core 18 bp T7 promoter, discovered by BLAST^89^ (Supp. Table 1). To measure gene expression (cellular mRNA) levels, intron-spanning primer pairs were selected from publications^90–94^ or pre-designed IDT PrimeTime assays. All primer sequences are listed in Supp. Table 1.

Two-step RT-qPCR was performed as follows. First, 10 μL first-strand cDNA synthesis reactions were prepared with up to 250 ng total RNA, 2 μL of 5X Maxima H Minus master mix or no RT control mix (Thermo Scientific #M1661), and nuclease-free water, and then incubated on a SimpliAmp thermal cycler using the kit-recommended protocol: 25°C for 10 minutes, 50°C for 15 minutes, and 85°C for 5 minutes, and then 10°C hold. Notably, Maxima cDNA synthesis is primed by both random hexamer and oligo-dT, enabling reverse transcription of both non-polyadenylated T7 RNA and endogenous mRNA. For each 20 μL qPCR reaction, 2–25 ng of RNA-equivalent cDNA reaction was mixed with 0.5 μM of each primer and PowerUp SYBR Green master mix (Applied Biosystems #A25742) by combining 10 μL of diluted cDNA reaction with 10 μL of a 2X mix of 1 μM primers and 2X PowerUp mix. Quantitative PCR was run on a QuantStudio 6 Flex Real-Time PCR system (Applied Biosystems, software version 1.3) with the following protocol: 50°C for 2 minutes, 95°C for 2 minutes, 40 cycles of [95°C for 15 seconds, 60°C for 1 minute], 95°C for 15 seconds, 60°C for 1 minute, and then 0.1°C/second ramp to 95°C for 15 seconds.

Relative expression levels were determined from instrument-calculated cycle threshold (C_t_) values by the comparative C_t_ method^48,95^. Reference assays targeting GSH1 and GSH2 (background signal) were used for IVT and unfixed IST samples. For fixed IST samples, GSH1 and GSH2 signals were below the limit of detection, so assays targeting *RPL24* and *RPS10* mRNA were used as reference. Results are represented as a difference of C_t_ (dC_t_, normalized to reference only) or a difference of differences (ddC_t_, normalized to reference and no knock-in control).

### Bulk RNA sequencing analysis

Four IVT reactions were performed on purified gDNA samples from K562 or Jurkat cells edited at *B2M* and *CTLA4*, respectively, by treating with either RNP alone or with the Superb-seq donor. An additional two “mock” IVT reactions without T7 RNA polymerase were performed, one per cell type (Supp. Table 2), and the six total RNA samples were purified and quantified. A pooled, ribosomal RNA-depleted RNA-seq library was made using the QIAseq UPXome RNA library kit (QIAGEN #334782, #334782) and SimpliAmp thermal cyclers. The pooled library contained 12 barcoded samples, with two technical library replicates per total RNA sample. All library preparation steps were performed according to kit instructions, with three modifications. First, cDNA synthesis was performed with 50:50 random hexamer and oligo-dT primers. Second, all bead cleanups were performed with 0.8X QIAseq beads (QIAGEN #333923). Third, library amplification was performed with the following thermal cycler protocol: 98°C for 3 minutes, 18 cycles of [98°C for 30 seconds, 55°C for 10 seconds, 72°C for 40 seconds], 72°C for 5 minutes, and then 4°C hold (ramp rate 2°C/second). Library cDNA was analyzed on a TapeStation 4150 (Agilent) and High Sensitivity D1000 ScreenTape (Agilent #5067–5584) and quantified on a Qubit 3 fluorometer using a Qubit dsDNA HS kit (Invitrogen #Q32851).

Paired-end sequencing was performed on a MiniSeq sequencer with Mid Output 300-cycle kit (Illumina) at the Salk Institute Next Generation Sequencing (NGS) Core, with this run configuration: 150/10/10/150 cycles (Read 1/i7 index/i5 index/Read 2), 0% PhiX. Sample demultiplexing and adapter/poly sequence trimming was performed using Cutadapt^96^. Read quality was assessed by FastQC (https://github.com/s-andrews/FastQC) and MultiQC^97^. Reads were aligned to human reference genome hg38 using BWA-MEM^98^ and post-processed using Samtools^99^. Track visualizations were generated using IGV^100^. See provided code for command details (Code Availability).

### Superb-seq profiling of chromatin remodeler genes in human K562 cells

Three K562 cell samples were prepared and frozen: (1) K562 cells treated by electroporation only (mock-treated, unedited), (2) a pool of five equally proportioned *ARID1A/SMARCA4* donor-edited cell lines, and (3) a pool of three equally proportioned *CHD3/CHD4* donor-edited cell lines (Supp. Table 2). Aliquots of each cell sample were thawed, and IST was performed on fixed cells as described above, with ~100,000 fixed cells per 40 μL reaction and duplicate reactions for each sample pool. After IST incubation for 18 hours, duplicate reactions were pooled, spun down at 200 × *g* for 10 minutes at 4°C, gently aspirated, resuspended in 40 μL fixation kit storage buffer, and passed through a 40 μm Flowmi cell strainer (SP Bel-Art #H13680–0040) to ensure a single-cell suspension. Singlets were counted and immediately processed by SPLiT-seq combinatorial barcoding using an Evercode WT Mini v2 kit (Parse Biosciences #ECW02110, #UDI1001), according to kit protocol. A total of 110k input cells were used. Sample proportions were 10% unedited cells, 45% *ARID1A/SMARCA4*-edited cells, and 45% *CHD3/CHD4*-edited cells. Barcoded singlets were counted (~20k total) and used as input for a 500 cell sequencing library and a 10k cell library. Library cDNA was analyzed using a TapeStation 4150 (Agilent) and D1000 ScreenTape (Agilent #5067–5582), and quantified on a Qubit 3 fluorometer using a Qubit dsDNA HS kit (Invitrogen #Q32851). Each library preparation yielded ~1 μg amplified cDNA library and ~400 ng of sequencing library of the expected size distribution for Split-seq libraries (Supp. Fig. 9C,D).

Paired-end sequencing was performed at the Salk NGS Core. The 500 cell library was run on a NextSeq 2000 sequencer with P1 300 cycle kit (Illumina), with 198/8/8/86 cycles (Read 1/i7 index/i5 index/Read 2), and 5% PhiX. The 10k cell library was run on a NovaSeq 6000 with SP 200 cycle kit (Illumina), 98/8/8/124 cycles, and 5% PhiX.

### Superb-seq read processing by Sheriff

A graphical overview of the Superb-seq read processing pipeline is detailed in Supp. Fig. 11. Briefly, the scRNA-seq FASTQ files were processed using Split-pipe v1.1.1 to generate an annotated BAM file (Supp. Fig. 11A). T7 barcoded reads were identified by a k-mer match (k=6) to the Superb-seq barcode in the 5’ soft-clipped sequence of mapped reads (Supp. Fig. 11B,C). These reads were considered “particular” edit events, due to occurrence in a particular cell (identified by read cell-barcode “CB” tag), the genomic mapping location of the base pair immediately upstream of the soft-clipped sequence (indicating the base-pair resolution repair site of the CRISPR-Cas9 edit event), and the soft-clip sequence (indicating part of the donor sequence upstream of the T7 promoter). Due to a k-mer match between the Superb-seq barcode and the template switch oligo (TSO) sequence, we constructed a set of observed variations in the TSO sequence from the soft-clipped sequences of the reads, which were considered “blacklist” sequences (Supp. Table 8). We then matched the Superb-seq barcode to each of the blacklisted TSO sequences to generate a list of problematic Superb-seq barcode k-mers. If a read’s soft-clipped sequence contained a match to a blacklist k-mer, we then performed k-mer matching between the soft-clip sequence and each of the blacklisted sequences. If the read had at least one additional k-mer match to the blacklisted sequences, and no additional non-blacklist k-mer matches to the Superb-seq barcode, then it was not classified as a barcoded T7 read. After processing the mapped reads, we identified a list of particular edit events, from which we then called “canonical” edit sites.

### Sheriff identification of Cas9 edit sites

After calling T7 barcoded reads to identify particular edit events, we then collapsed particular edit events to canonical edit sites, each of which represents the most common version of an edit across cells. By associating particular edit sites with a canonical edit site, we were able to further filter edit events for false-positive T7 barcoded read calls, based on metrics for each canonical edit site obtained from aggregating information across cells.

To determine canonical edit events, we first constructed a graph per chromosome using Faiss (https://faiss.ai), where each node is a particular edit site, and edges between nodes represent the base-pair distance between the particular edit sites (Supp. Fig. 11D). Particular edit sites were then processed in order of the number of cells that have the particular edit, denoted as an ordered set P (Supp. Fig. 11D). The first particular edit in $P,\;P_{0}$, is considered the first canonical edit site. All particular edits within a defined base-pair distance (140 bp) from the current canonical edit site ($P_{0}$ for the first iteration) are considered particular edits of the canonical edit site, denoted $P_{c,0}$. The ordered set of particular edit sites is then updated, removing the current canonical edit site and the associated particular edit sites, such that $P=P-(P_{c,0}\cup \{P_{0}\})$. This process is repeated at each iteration, until all particular edit sites are associated with a canonical edit site, resulting in the stopping condition P=Ø (Supp. Fig. 11D).

High confidence canonical edit sites were then determined by the following criteria; 1) the canonical edit site was supported by occurrence in at least 3 cells, 2) there were particular edit events that occurred in both the forward and reverse direction, indicating Superb donor insertion in either orientation, 3) there were no more than 15 bp between the two closest pairs of forward and reverse particular edit sites (indicating low variation of particular edits around the canonical edit site position), and 4) the canonical edit site did not overlap blacklisted genome regions (Supp. Table 8). For criteria 4, the blacklisted genome regions consisted of: 1) 17 genome regions in the unedited sample with excessive read mapping similar to ENCODE blacklist regions^101^, 2) genome locations with sequence similarity to the Superb-seq barcode in both the forward and reverse direction in close proximity identified from the alignment, which when combined with sequencing errors creates soft-clip sequences that resemble Superb-seq barcodes, and 3) simple repeat regions of poly-N sites of length greater than 50 bp, which accumulated reads with soft-clipped sequences resulting in false-positive canonical edit site calls. Excessive read mapping regions were identified by downsampling reads from the unedited sample to 10% of the total reads, and peak calling with Macs2^102^ using macs2 callpeak --broad --nomodel --extsize 98 --keep-dup all --broad-cutoff 0.0000000001 -g hs on the resulting BAM file. Simple repeat regions were downloaded and subsetted from: https://s3.amazonaws.com/igv.org.genomes/hg38/rmsk/hg38_rmsk_Simple_repeat.bed.gz

Automated filtering of canonical edit sites as described above identified 63 candidate canonical edit sites. We manually examined the alignments of the called T7 barcoded reads at these edit sites in the Integrative Genome Viewer (IGV)^100^ (Supp. Material 1). 20 of the 63 sites did not appear to be true edit sites, but rather were sites with similarity to the Superb-seq barcode and where mismatches in the read (due to sequencing errors or mutations in the K562 genome) resulted in 5’ soft-clipping of the alignment and incorrect edit calls (Supp. Material 1). Aside from examining the alignment, these false positives also differed by other metrics when compared to the final 43 confident edit sites, including specificity of the edit event for cells that were exposed to the same treatment (Supp. Fig. 23A–B), the frequency of the canonical edit across cells (Supp. Fig. 23C), the best-matching alignment rate to the set of guide sequences (determined below, Supp. Fig. 23D), the distance between the best-matching candidate Cas9 edit site based on guide sequence/PAM sequence similarity and the called canonical edit site location (Supp. Fig. 23E), and the minimum distance between the forward and reverse T7 barcoded reads at the candidate edit site (Supp. Fig. 23F). Therefore only the 43 confident edit sites were kept for further downstream analyses.

### Performance assessment of T7 read processing

The sensitivity, specificity, and false discovery rate (FDR) of T7 read calling were estimated as follows. A positive set of pure T7 reads was approximated from regions strongly enriched for T7 reads by filtering total aligned reads for all reads within a 200 bp window (± 100 bp) of the expected on-target Cas9 cleavage site of each guide RNA (Supp. Table 1). A negative set of non-T7 reads was defined as non-edited sample reads, downsampled 100x. Sensitivity (true positive / positive set) was estimated from the proportion of positive set reads that were called T7 reads (true positive). Specificity, 1 – (false positives / negative set), was estimated from the proportion of negative set reads that were called T7 reads (false positive). The FDR (false positives / total positives) was estimated from the proportion of total positives (true positives + false positives) that were false positives.

### Sheriff quantification of single-cell edit allele dosage

At each called canonical edit site, we stratify the particular edits associated with the canonical edit by cell barcode (previously corrected for sequencing error by Split-pipe). To determine if particular edits were generated from different alleles, we performed pairwise comparison of all of the particular edits in each cell (Supp. Fig. 11F). Two particular edits were considered different if they 1) had a different mapping orientation, or 2) had a different reference genome mapping position. If two particular edits had the same orientation and the same reference genome position, we then examined the 5’ clipped region of their alignments to detect sequence variation within, or flanking, the inserted donor DNA.

To compare the clipped sequences, we accounted for several confounding factors that could introduce sequence variation, including read fragmentation during library preparation, the presence of the TSO within the 5’ end of the read and sequencing errors resulting in mis-called bases or incorrect homopolymer runs. We first performed local alignment between the sequences using pairwise2.align.localms with Biopython, with matching score +1, mismatch −1, −0.5 for gap open penalty, and −0.5 for gap extension penalty^103^. We then counted the number of mis-matched base pairs until all bases of the shorter donor sequence were seen. This results in an edit distance of 0 if one donor sequence is a subsequence of the other, and accounts for 5’ read fragmentation. If the edit distance was 2 or more, we accounted for the possibility of homopolymer sequencing errors^104^ by re-calculating the edit distance between the sequences after replacing homopolymer runs of length ≥ 3 with a single instance of the repeated base^104^. If the edit distance between the two particular edits was still more than 2 mismatches, we re-calculated the edit distance by counting the number of mis-matches in the first 10 base pairs of alignment between the sequences starting from the 3’ end of the alignment (to account for sequencing artifacts on the 5’ end of the read).

If the edit distance between the two particular edits (after accounting for homopolymer error and 5’ read error) was 2 or less, we considered the particular edits to be the same, and we added the longer of the two particular edits to a candidate longest edit list, and removed the smaller particular edit from the list (if present). If, however, the two particular edits had more than 3 mismatches, or had different orientations or reference genome map positions, then they were both considered candidate longest edits and added to the list. After completing all pairwise comparisons of clipped reads, the cell edit allele count was determined from the number of sequences in the candidate longest edits list. The edit allelic dosage calling algorithm is implemented in Python and is available within Sheriff (Code Availability).

To call edit allele dosage per gene, gene start and end positions were intersected with canonical edit sites (defined as canonical edit position ± 140 bp). Single-cell gene edit dosages were then determined as the sum of the edit alleles for each cell across all intersecting edit sites of a given gene. The number of called edit alleles was capped to the copy-number for the given gene in K562 cells, determined from deep whole genome sequencing of K562 cells^105^.

### Sheriff quantification of single-cell gene expression

To quantify gene expression while correcting for reads generated from the T7 polymerase at Cas9 edit sites, we first determined sets of UMIs stratified by unique cell barcode and gene using “CB”, “GN”, and “GX” read tags from the annotated BAM file generated by Split-pipe (Supp. Fig. 11E). We then filtered reads that were ± 1000 bp from a canonical edit site across all cells, to remove reads that could have been generated from the T7 polymerase (Supp. Fig. 11E). The remaining reads were then used to count the number of unique UMIs per gene per cell, accounting for sequencing error by counting the number of UMIs that are distant by more than 1 bp (Supp. Fig. 11E). To account for cases where one or more UMIs had a hamming distance > 1 from one another but were within 1 base pair of a common UMI, we constructed UMI sets where within each set there existed a single edit distance path between the UMIs, similarly to previous methods^106^. The number of these sets was then counted as the number of unique molecules for a given gene and cell, effectively accounting for sequencing error and removing confounding non-barcoded reads generated from the T7 polymerase IST reaction (Supp. Fig. 11E). Normalisation was then performed per cell, dividing the UMIs per gene by the total UMIs within the cell, multiplying by the median library size observed across cells, and then log-scaling after adding a +1 pseudocount (as implemented in Scanpy)^52^. Scanpy with default parameters was also used to determine a UMAP of the cell gene expression using the cosine metric to construct the k-nearest-neighbour graph. The sc.tl.score_genes_cell_cycle function was used to determine the cell cycle annotations for each cell. Significantly different populations of cells were determined using Cytocipher^53^ with a p-value threshold of 0.01 and initial clusters determined using default Leiden clustering^52,107^.

### Comparison of 500 cell and 10k cell Superb-seq libraries

The 500 cell Superb-seq library was processed with Split-pipe using the same parameters as the 10k cell library above. Sheriff was then used to process the 500 cell Split-pipe BAM file using the same parameters as the 10k cell library, except using a minimum of 1 cell edited for canonical edit site calling due to the smaller total cells in the library. Since some of the Cas9 edit sites detected in the 10k cell library did not have T7 barcoded regions indicating donor insertion in each orientation in the 500 cell library, we generated a white-list for confident Cas9 edit sites (n=43) from the 10k cell library. We then called Cas9 edit sites at these locations in the 500 cell library if a T7 barcoded read was detected within 140 bp from an edit site detected in the 10k cell library. This enabled determination of the frequency of edited cells for an additional 18 edit sites (Supp. Fig. 15C). As with the 10k Superb-seq library, we observed some false-positive edit-site calls due to read sequencing errors causing soft-clipped reads with similarity to the Superb-seq barcode due to reference genome sequence similarity. These were also determined by examining the alignment of barcoded T7 reads as for the 10k cell library. Confident edit sites constituted 30/40 called edit sites (Supp. Table 3). To compare whether the expected proportion of edited cells was maintained between the 500 cell and the 10k cell library, despite the higher sequencing depth of the former, we plotted the number of edited cells per edit site detected in the 10k cell library, filling in edit sites that were not detected in the 500 cell library with zeroes (Supp. Fig. 15G). We also plotted the expected number of edited cells in the 500 cell library against the observed number of edited cells. The expected number of edited cells ($E\left[{\mathrm{editedcells}}_{500\mathrm{cell}}\right]$) was determined by Equation 1,

(1) $$E\left[{\mathrm{editedcells}}_{500\mathrm{cell}}\right]=\left(\frac{{\mathrm{editedcells}}_{10k}}{{\;\mathrm{totalcells}}_{10k}}\right)\times 500$$

where ${\mathrm{editedcells}}_{10k}$ indicates the number of edited cells for the respective edit site in the 10k cell library, and ${\mathrm{totalcells}}_{10k}$ is the total number of cells in the 10k cell library. The same approach was also used to compare the number of edited alleles detected per edit site between Superb-seq libraries (Supp. Fig. 15H). After calculating these expected frequencies, we then determined Pearson’s correlation and p-value between the expected and observed measurements (for both the estimated number of edited cells and total edited alleles). The p-value for these correlations was calculated using a two-sided exact test for Pearson’s correlation coefficient assuming the beta distribution.

### Inference of causal guides from off-target edit sequences

To infer the guide associated with an identified canonical edit site, we used a combination of sequence similarity to each of the 7 guides used in our experiment, and the co-occurrence of each edit-site with each of the on-target edit sites associated with the guides. To determine the sequence similarity, we first identified all PAM sites (identified as sites with the canonical “NGG” or alternative “NAG” sequence^7^) on both the forward and reverse strand within ± 100 bp from each canonical edit site. We then extracted the 20 bp of reference genome sequence upstream of each PAM site as candidate target sequences. Each guide sequence was then aligned to each of the candidate target sequences using Biopython pairwise2.align.localms, with scoring parameters: +1 for match, −1 for mismatch, −0.8 for gap, and −0.5 for gap extension^103^. The similarity between the guide sequence and candidate target sequences was determined as the number of base-pairs which were aligned. We also scored guides by taking the cosine similarity between the barcoded T7 UMI counts across cells for the guide on-target edit site and each canonical edit site. These cosine similarities between on-target edit sites and all called edit sites were then scaled by the sum of the cosine similarities across all on-targets. If the similarity and cell co-occurrence metrics prioritised the same causal guide for a given canonical edit site, then this candidate guide was called as the causal guide for the canonical edit site. If the canonical edit site had no co-occurrence with an on-target edit site, then the top-matching guide by sequence similarity was called as the causal guide. If the sequence-based guide inference and coincidence based guide inference disagreed on the casual guide, we then utilised a joint score to determine the highest-scoring guide, that considered both the similarity and co-occurrence scores (Equation 2),

(2) $$g_{\mathrm{score}}={\left(\frac{h}{h_{l}}\right)}^{10}-1+\frac{c}{c_{l}}$$

where $g_{\mathrm{score}}$ is the joint guide score, h is the similarity between the candidate guide sequence and the canonical edit site, $h_{l}$ is a constant ($h_{l}=\;15$), c is the coincidence score, and $c_{l}$ is also constant ($c_{l}=0.7$). The causal guide was taken as the guide with the highest $g_{\mathrm{score}}$.

### Differential gene expression

To call differentially expressed genes with respect to the number of edit allele calls, we developed an approach inspired by Perturb-seq linear models to call single-cell edit effects^3^, but also allowing for non-linear effects of confounding variables similar to Mixscape^54^. For a given edited gene, we first determine a set of cells consisting of the unedited control cells and all cells with at least one edit allele call in the condition associated with the given edited gene (e.g. NuRD sample cells if *CHD4* is the edited gene). For each edited cell in ${cells}_{edited}$, an example cell from the unedited control population is selected with the most similar cell state and number of genes detected. Only alleles with more than 25 example cells were considered. The most similar control cell was determined as the first cell with less than 1,000 detected genes different to the query edited cell within the top 10 nearest neighbor cells. The top 10 nearest neighbor cells were determined by manhattan distance comparing the z-score standardized UMAP coordinates (cell-state) and z-score normalized number of genes detected within the cell. This effectively constructs “allelic pairings” of cell groups (g), whereby within each group cells are matched by cell state and genes detected, but differ by gene edit allele dosage.

To call differentially expressed genes with respect to a given edited gene, a linear mixed model was used to model the expression, $y_{ij}$, of query gene i in cell j (Equation 3),

(3) $$y_{ij}\;\sim \;\beta \times a_{ej}+Z_{k}\times g_{k}+\epsilon$$

where β is the fixed effect of the number of edit alleles $a_{ej}$, of gene e in cell j, and $Z_{k}$ is the random effect of cell group $g_{k}$. Gene expression was normalized using Sctransform Pearson residuals^108^ prior to the linear mixed modeling. This procedure effectively removed correlation between edit allele detection and the number of genes detected per cell (Supp. Fig. 24A–C). For *SMARCA4* in particular, however, higher gene expression was observed for cells with 2 edit alleles compared with 1 (Supp. Fig. 24D). Boot-strapped mean estimates of the genes detected per cell after the edit allele strategy showed systematically higher genes detected for cells in the 2 edit allele *SMARCA4* group (Supp. Fig. 24E). While this trend also held for *CHD3* (controls ~7.3k genes detected vs. 7.7k genes detected 2 edit allele calls) and *CHD4* (controls ~7.1k genes detected vs. 7.8k genes detected 2 edit allele calls), the range of difference was much higher for *SMARCA4* (*SMARCA4* controls ~7.9k genes detected vs. 8.9k genes detected 2 edit allele cells). This could be due to few captured control cells (n=683) relative to the NuRD sample (n=5086) and BAF sample (n=3730) resulting in few cases of control cells with high gene detection counts. To account for the systematic gene detection difference between the 2 edit alleles, for edits with > 1 edited allele detected, these were modeled as per Equation 4,

(4) $$y_{ij}\;\sim \;\beta \times a_{ej}+\gamma \times a_{k}+Z_{k}\times g_{k}+\epsilon$$

where $\alpha_{k}$ is the edit alleles of the grouping k (i.e. is a shared allele group label applied to both the edit and control cells) and γ is the fixed effect of $\alpha_{k}$ on gene expression. Hence, control and edited cells are additionally adjusted for the observed bias in gene detection rate with increased edit allele rate (Supp. Fig. 24E, Figure 5B).

A two-sided Wald test was used to calculate p-values, and the Benjamini-Hochberg method was used to adjust p-values for multiple hypothesis testing. Genes were called as differentially expressed if the adjusted p-value < 0.01 and > 0.2. This approach enabled calling DEGs across the observed range of gene expression (Supp. Fig. 21F–J).

We tested edited genes ($g_{e}$) if the edit was present in > 30 cells and the gene’s mean expression was > 0.2. This resulted in selection of our on-target genes (*CHD3*, *CHD4*, *ARID1A*, and *SMARCA4*) and several off-target genes (*USP9X*, *CDC27*, *FBXO38-DT*, and *CNIH3*) as adequately expressed and edited. We then tested whether each of these expressed edited genes were differentially expressed relative to the number of edit alleles for that gene. For the subset of genes that had differential expression relative to the number of edit alleles for that gene (*CHD3*, *CHD4*, *ARID1A*, *SMARCA4*, and *USP9X*), we then tested for differential expression between their number of edit alleles and the expression of highly variable genes. Highly variable genes were determined using Scanpy, with parameters min_mean=0.2 min_disp=0.6 max_mean=5. Additionally, we also included genes which were determined to be NuRD or BAF regulated genes in K562 cells (details below), with min_mean=0.03 min_disp=0 for NuRD targets, and min_mean=0.2 min_disp=0.3 for BAF targets.

### Gene set enrichment analysis

We constructed gene sets of NuRD and BAF targets for subsequent gene set over-enrichment analysis within our DEGs called with respect to our NuRD knock-down cells (*CHD3*/*CHD4* edited cells) and BAF knock-down cells (*ARID1A*/*SMARCA4* edited cells) (Supp. Table 5). NuRD targets were determined by downloading ENCODE hg38 IDR-thresholded CHD4 ChIP-seq peaks from K562 cells (file accession ENCFF985QBS)^61^. These peaks were then intersected with hg38 gene transcription start sites (TSSs) to determine NuRD target genes (Supp. Table 5). For BAF target genes, we downloaded ENCODE hg38 pseudo-replicated ATAC-seq peaks (file accession ENCFF333TAT) and IDR-thresholded SMARCA4 ChIP-seq peaks from K562 cells (file accession ENCFF267OGF)^61^. The K562 ATAC-seq peaks and the SMARCA4 ChIP-seq peaks were then intersected to obtain K562 open SMARCA4 binding sites. The open SMARCA4 bindings sites were then intersected with gene TSSs to obtain BAF promoter target genes. Since BAF can also act at gene enhancers^65,109^, we additionally downloaded hg19 K562 enhancer-gene associations from EnhancerAtlas 2.0^110^. The enhancer hg19 genomic coordinates were then converted to hg38 using LiftOver^111^, and intersected with the open SMARCA4 peaks. Genes associated with the enhancer genome annotations that overlapped the open SMARCA4 ChIP-seq peaks were then considered BAF enhancer target genes (Supp. Table 5).

USP9X functional gene sets were determined from intersecting NRF1 (one of the top DEGs specific to *USP9X* editing) ChIP-seq peaks from K562 cells (ENCODE file accession ENCFF259YUE) with gene transcription start sites and open chromatin regions (file accession ENCFF333TAT). The “hallmark G2M checkpoint” from the molecular signatures database was used for the G2M checkpoint gene set^112^ (Supp. Table 5).

After using a two-sided Fisher’s exact test to determine association between the DEGs and each of the functional gene sets, we then used the Benjamini-Hochberg method for false-discovery-rate correction of p-values, and took associations with p < 0.05 as significant associations (Supp. Tables 6, 7). Odds-ratios and associated two-sided 95% confidence intervals were calculated from the contingency tables of overlaps between DEGs and functional gene sets (Supp. Table 6) using the conditional maximum likelihood estimation method.

## Supplementary Material

Supplement 1

Supplement 2

## Data Availability

All Superb-seq and bulk RNA-seq datasets are available on the GEO repository, accession number GSE284207. K562 ChIP-seq and ATAC-seq datasets were downloaded from the ENCODE data portal (https://www.encodeproject.org) using the following identifiers: ENCFF267OGF for SMARCA4 ChIP-seq, ENCFF985QBS for CHD4 ChIP-seq, ENCFF259YUE for NRF1 ChIP-seq, and ENCFF333TAT for ATAC-seq.

## Footnotes

Code availability Sheriff, the software package that quantifies single-cell edit allele dosage and gene expression from aligned Superb-seq reads, is available on GitHub (https://github.com/BradBalderson/Sheriff). Code for additional bulk RNA-seq and Superb-seq analyses is also available (https://github.com/BradBalderson/superb_analysis).
