# Supplementary material for: Joint single-cell profiling of Cas9 edits and transcriptomes reveals widespread off-target events and effects on gene expression
Source: bioRxiv. 2025 Aug 28:2025.02.07.636966. Preprint. [Version 2] doi: 10.1101/2025.02.07.636966 (PMC12407703; doi:10.1101/2025.02.07.636966)
Supplement: Supplement 1 [file media-1.pdf]

Joint single-cell profiling of Cas9 edits and transcriptomes reveals widespread off-target events and effects on gene expression

Michael H. Lorenzini*, Brad Balderson*, Karthyayani Sajeev, Aaron J. Ho, Graham McVicker | ***** These authors contributed equally

Supplementary information

Supplementary Note 1. The Superb-seq donor DNA disrupts target gene expression
Supplementary Note 2. T7 promoter labeling results in two rates of small indel formation
Supplementary Note 3. Development of *in vitro* and *in situ* transcription of Cas9 edit sites

Supplementary Figure 1. Development of Cas9 edit labeling with phage T7 promoter
Supplementary Figure 2. Superb-seq donor insertion disrupts target gene expression
Supplementary Figure 3. Bimodal edit fate associates with guide RNA sequence
Supplementary Figure 4. Generation of *in vitro* T7 transcripts at targeted genome edits
Supplementary Figure 5. Generation of *in situ* T7 transcripts in nuclei and fixed cells
Supplementary Figure 6. Optimization of *in situ* transcription in paraformaldehyde-fixed cells
Supplementary Figure 7. *In situ* transcription at Cas9 edits to chromatin remodeler genes
Supplementary Figure 8. Sequencing of *in situ* T7 transcripts identifies Cas9 genome edits
Supplementary Figure 9. Generation of a joint Superb-seq library of T7 and endogenous RNA reads
Supplementary Figure 10. Sequence structure of Superb-seq paired-end library fragments
Supplementary Figure 11. Sheriff, a command line tool to quantify Superb-seq edits and transcriptomes
Supplementary Figure 12. Performance of T7 read identification by Sheriff
Supplementary Figure 13. UMI and gene count distributions across Superb-seq sample pools
Supplementary Figure 14. Transcriptional heterogeneity of Superb-seq single-cell gene expression
Supplementary Figure 15. Reproducible edit capture in 500 cell and 10k cell libraries
Supplementary Figure 16. T7 read features indicate multiple edit alleles within individual cells
Supplementary Figure 17. Co-occurrence of on- and off-target Cas9 edit alleles within individual cells
Supplementary Figure 18. Sequence similarity identifies off-target causal guide RNAs
Supplementary Figure 19. Superb-seq captures guide-specific editing profiles from pooled samples
Supplementary Figure 20. Sequence similarity between off-target sequences
Supplementary Figure 21. Differential gene expression associates with Cas9 edit allele dosage
Supplementary Figure 22. Off-target *USP9X* edit site intersects an SP1 binding motif
Supplementary Figure 23. Multiple independent metrics confirm confident Cas9 edit sites
Supplementary Figure 24. Correction of gene expression estimates by allelic pairing

Additional files

Supplementary Table 1. (.xlsx) Oligonucleotide sequences for donor DNA, guide RNA, and PCR primers
Supplementary Table 2. (.xlsx) Cas9 editing conditions for primary and immortalized cells
Supplementary Table 3. (.xlsx) Sheriff called edit sites for 10k and 500 cell Superb-seq libraries
Supplementary Table 4. (.xlsx) Differential gene expression summary statistics
Supplementary Table 5. (.xlsx) NuRD, BAF, G2/M, and NRF1 functional gene sets
Supplementary Table 6. (.xlsx) Contingency tables intersecting DEGs with functional gene sets
Supplementary Table 7. (.xlsx) Gene set enrichment analysis summary statistics
Supplementary Table 8. (.xlsx) Blacklisted sequences and genome regions for edit site calling
Supplementary Table 9. (.xlsx) Tracking indels by decomposition (TIDE) data and analysis parameters
Supplementary Table 10. (.xlsx) UV spectroscopy data for T7 in vitro transcription
Supplementary Table 11. (.xlsx) BLAST results for T7 promoter sequence alignment to hg38
Supplementary Table 12. (.xlsx) Reverse transcription quantitative PCR (RT-qPCR) data
Supplementary Table 13. (.xlsx) Quality metrics from Split-pipe analysis of Superb-seq reads
Supplementary Table 14. (.xlsx) Flow cytometry data
Supplementary Material 1. (.pdf) 10k library T7 barcoded read alignments centered on called edit sites

Supplementary Note 1. The Superb-seq donor DNA disrupts target gene expression

To maximize gene disruption upon insertion of donor DNA into coding sequences (**Supp. Fig. 2A**), we set the length of all donor designs to *3n+1* to yield +1 shifts upon precise insertion and +2 shifts upon the common 1 base pair (bp) templated insertion by Cas9[1] (**Supp. Table 1**). Additionally, the Superb-seq donor DNA (design 02) encodes stop codons in three reading frames (**Supp. Fig. 2B**). Consistent with its design, > 90% of observed donor insertions generated +1/+2 frame-shifts (**Supp. Fig. 2C**). To confirm that donor insertion triggers nonsense-mediated decay (NMD) of target genes, we measured gene and protein expression by reverse-transcription qPCR (RT-qPCR) and flow cytometry. Donor-edited cells exhibited target transcript knockdown consistent with NMD (**Supp. Fig. 2D**). Furthermore, in a sample of cells with 40% donor knock-in alleles and < 10% indel alleles (**Supp. Fig. 2E**), we observed knockdown of B2M target protein in 50% of cells (**Supp. Fig. 2F**). Together, these results establish that homology-free knock-in of our donor DNA achieves high-efficiency labeling of Cas9 edits and knockdown of targeted genes.

Supplementary Note 2. T7 promoter labeling results in two rates of small indel formation

We observed that delivery of RNP and donor DNA resulted in two discrete outcomes: a "high indel" outcome with a high rate of small indels but a low rate of donor insertions, and a "low indel" outcome with a low rate of small indels and the potential for a very high rate of donor insertions (**Fig. 1E, Supp. Fig. 1E**). On average, the low indel group had a 3 fold lower rate of short indels (24.0% vs. 78.8%) and a 5 fold higher rate of donor insertions (34.9% vs. 6.8%) (**Supp. Fig. 1E**). To explain this bifurcated editing fate, we looked for associations of edit outcome with guide RNA, cell type, and genome site. In the subset of 49 samples treated with guide RNAs that were used in two or more samples (excluding Jurkat samples), we observed that a given guide always generated the same type of outcome (**Supp. Fig. 3B,E**). Editing outcome was not associated with cell type or genome site (**Supp. Fig. 3C,D,F,G**). We compared guide protospacer sequences within each outcome group and observed that most low-indel guides contained a guanine at protospacer position -2 (**Supp. Fig. 3H**), a feature previously associated with increased Cas9 cleavage efficiency[2,3]. High-indel guides exhibited limited sequence similarity (**Supp. Fig. 3I**). These results suggest that certain guides give optimal Cas9 editing efficiency for minimizing small indel formation and maximizing homology-free donor insertion.

Supplementary Note 3. Development of *in vitro* and *in situ* transcription of Cas9 edit sites

As a first step toward IST of inserted phage promoters, we started with *in vitro* transcription (IVT) on purified genomic DNA (**Fig. 2A**). RNA was only generated in the presence of T7 polymerase and not in its absence, indicating functional T7 transcription (**Supp. Fig. 4A**). However, RNA was generated in the absence of donor DNA insertion (**Supp. Fig. 4A**). Since T7 polymerase activity is promoter-specific[4], we suspected that RNA was generated from endogenous human sequences that are homologous to the T7 promoter. To examine this, we aligned the canonical 18 bp T7 promoter sequence (5'-TAATACGACTCACTATAG-3')[4] to the human reference genome (hg38) using BLAST[5] and identified 14 sequences with at least 90% sequence similarity (16/18 bp), and five sequences containing an terminal guanine that initiates T7 transcription (**Supp. Table 11**)[6]. To determine whether these endogenous sites engage the T7 RNA polymerase, we selected the three most similar sequences (17/18 bp similarity and terminal guanine) and quantified T7 RNA extension at these sites in three cell types by reverse-transcription quantitative PCR (RT-qPCR) (**Fig. 2A**). Two of the three sequences gave 50 fold induction of T7 RNA compared to five control sites without sequence similarity (**Supp. Fig. 4B,C**). This indicates that endogenous T7 promoters are a potential source of background T7 transcription.

Next, we performed T7 IVT on genomic DNA from four cell types (including primary T cells) with promoter-labeled edits at two genome sites (*B2M* and *CTLA4*). We quantified T7 RNA by RT-qPCR and observed consistent 500 fold induction of T7 RNA at promoter-labeled Cas9 edit sites (**Fig. 2B,C**), which was 10 fold higher than the strongest background promoter identified by BLAST (**Supp. Fig. 4C**). Next we characterized the length and direction of T7 RNA extension. Most transcripts extended < 1 kilobase (kb) from the knock-in site, with a small fraction of transcripts extending > 10 kb away (**Supp. Fig. 4C,D**), indicating a concentration of short T7 transcripts near to Cas9 cleavage sites. At background sites, we observed T7 RNA only downstream (3') of the promoter sequence. At Cas9 edits however, T7 RNA extended in both directions with similar abundance, indicating that homology-free knock-in randomly inserts the T7 promoter sequence in both possible orientations (**Supp. Fig. 4B–D**). This bi-directional signature distinguished genuine Cas9 edit sites from background T7 transcription occurring at endogenous genome sequences that resembled the T7 promoter sequence. Together, these IVT results demonstrate that promoter-labeled Cas9 edits encode a functional T7 promoter that can be used to report Cas9 edit events with distinct RNA features.

Next, to determine the performance of T7 RNA generation on intact chromatin within nuclei, we performed T7 IST on nuclei isolated in ATAC-seq buffer[7] (**Fig. 2D**). We performed nuclei isolation on three cell lines with promoter-labeled Cas9 edits at *B2M* or *CTLA4*, followed by immediate IST, total RNA extraction, and RT-qPCR. Our results were comparable to those from IVT on genomic DNA, with up to 500 fold induction of T7 RNA at donor

insertions (**Fig. 2E,F, Supp. Fig. 5A–D**), indicating effective IST on chromosomal DNA. IST transcripts were not detectable at the 10 kb range at either loci (**Supp. Fig. 5A–C**), suggesting that nuclei IST generates shorter, more localized T7 RNA compared to IVT (**Supp. Fig. 4C,D**). Next we compared T7 RNA abundance to that of endogenous mRNA from *RPL24*, an extremely expressed ribosome subunit. We observed that levels of T7 RNA at *CTLA4* approached those of *RPL24* mRNA (**Supp. Fig. 5A,B**). Levels of T7 RNA at the *B2M* locus were more difficult to compare due to elevated *B2M* mRNA background (**Supp. Fig. 5,C,D**), indicating that downstream sequencing analysis would require distinguishing reads from T7 and endogenous transcripts. These results establish that IST can generate high levels of edit-marking T7 RNAs.

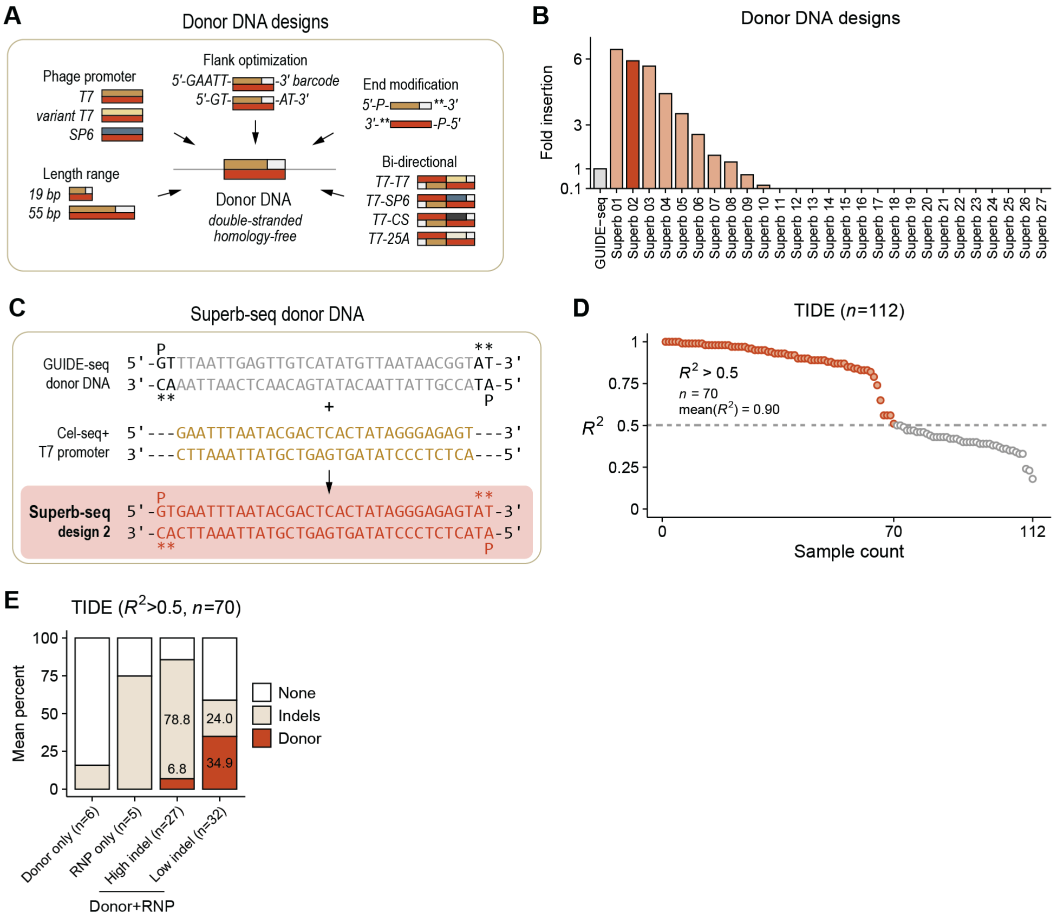

Supplementary Figure 1. Development of Cas9 edit labeling with phage T7 promoter. (**A**) Design categories of 27 candidate Superb-seq donor DNA constructs. (**B**) Insertion rate of the 27 candidate Superb-seq donor designs relative to the GUIDE-seq donor, measured by TIDE. Value for design 2 is the mean of four samples. (**C**) Sequences of the GUIDE-seq donor[8], Cel-seq+ T7 promoter[9], and the derived Superb-seq donor. Terminal 5' phosphate (P) and 3' phosphorothioate (*) modifications are indicated. (**D**) TIDE R-squared (R^2) values from 112 Sanger sequencing samples. Samples with R^2 > 0.5 (n = 70) were used for downstream analyses, and are indicated in red. (**E**) Frequency of editing outcomes (mean percent) for indicated sample groups. Possible editing outcomes are unedited (none), donor-less edit (Indels), and donor insertion (Donor, +30 bp insertion or greater).

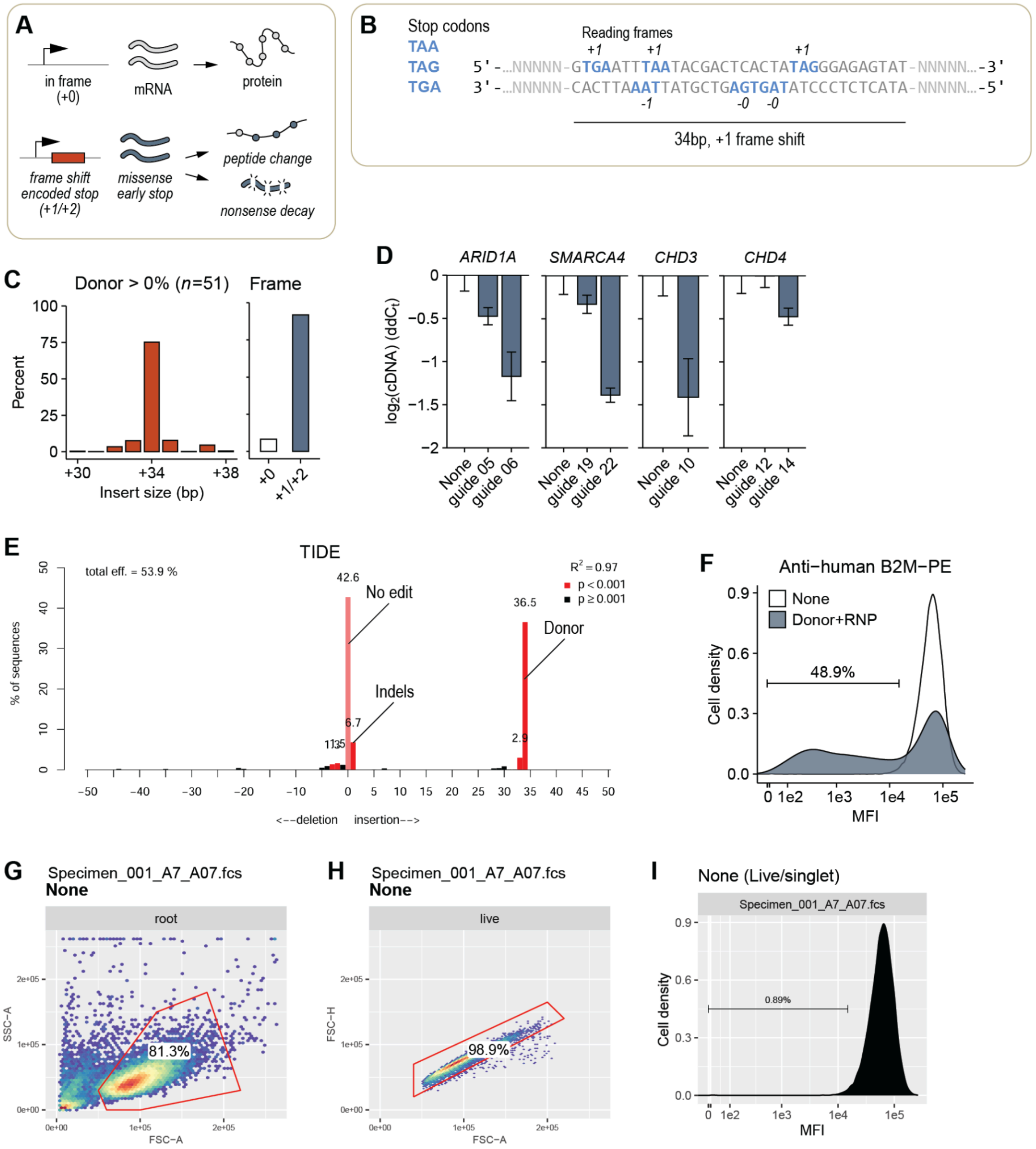

Supplementary Figure 2. Superb-seq donor insertion disrupts target gene expression. (**A**) Mechanisms of gene disruption by donor insertion events. (**B**) Stop codons and frameshift inserted by Superb-seq donor DNA. Stop codon reading frames indicated. (**C**) Frequencies of donor insertion events by size in 51 samples with detectable donor insertion, and cumulative frequency of in-frame (+0) or frameshift (+1/+2) events. (**D**) Expression of chromatin remodeler genes in K562 cells after donor+RNP editing with the indicated guide, or without editing (None, electroporation with buffer only), measured by reverse transcription qPCR (RT-qPCR) and the comparative threshold cycle (C_t) method[10]. Results are represented as a difference of differences in C_t values (ddC_t) normalized to reference genes *RPL24/RPS10*, and the unedited cell sample. Bars represent the mean of technical replicates (n = 3), and whiskers represent 2 × SEM. (**E**) Indel distribution of *B2M*-edited cells, measured by TIDE. (**F**) Median B2M fluorescence intensity (MFI) of unedited (None) and *B2M* edit-labeled (Donor+RNP, guide 4) GM12878 cells, measured by flow cytometry. Cells were gated on live singlets. (**G–I**) Representative flow cytometric gating for (**G**) live, (**H**) singlet, and (**I**) B2M-knockdown events represented in **F**.

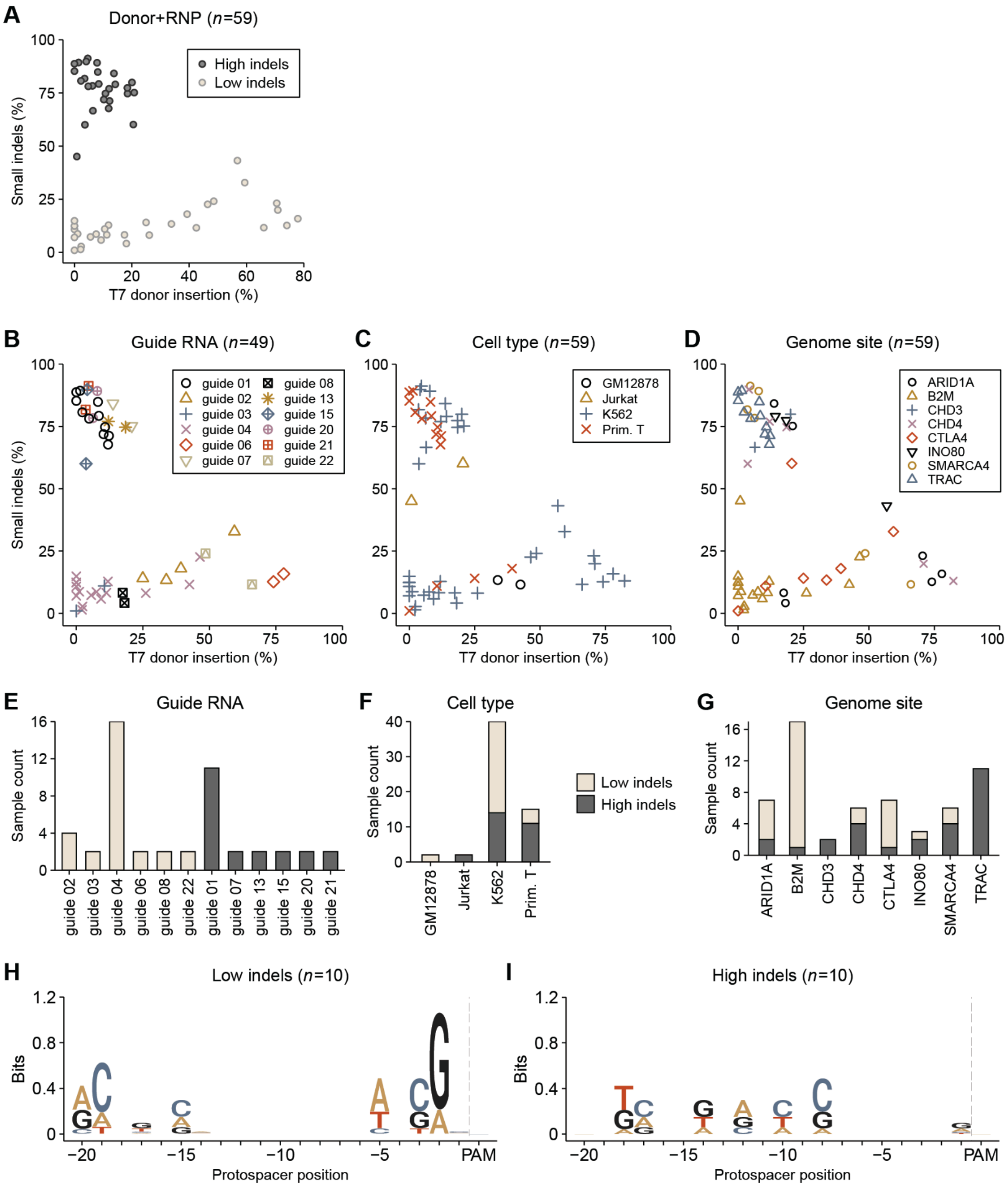

Supplementary Figure 3. Bimodal edit fate associates with guide RNA. (**A–D**) Frequency of donor insertion and variable small indel events in 59 knock-in samples (Donor+RNP) labeled by (**A**) outcome group (High or Low indels), (**B**) guide RNA, (**C**) cell type, or (**D**) target genome site. (**E–G**) Number of "high indel" or "low indel" samples, grouped as in **B–D**. (**H,I**) Positional Shannon entropy (bits) of 20-bp protospacer sequences for (**H**) the 10 guide RNAs used in the 32 "low indel" samples, or (**I**) the 10 guides used in the 27 "high indel" samples, calculated using ggseqlogo[11].

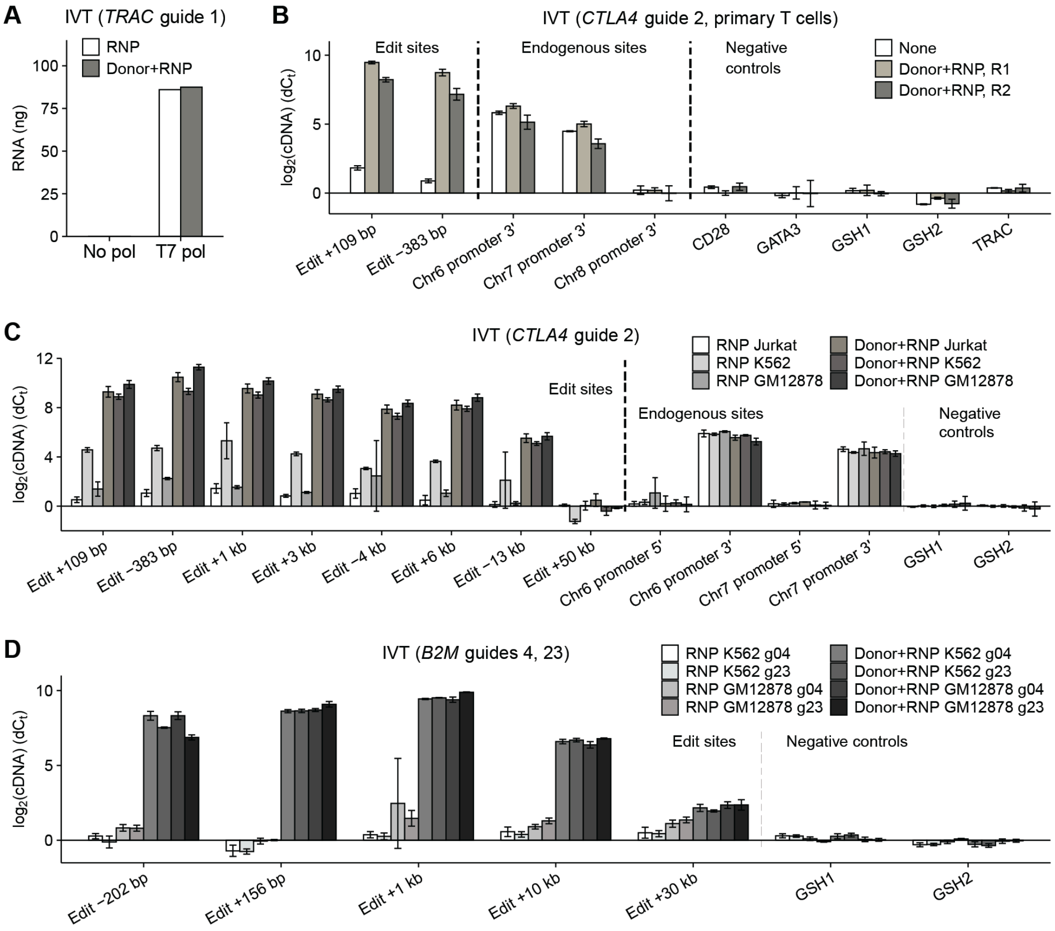

Supplementary Figure 4. Generation of *in vitro* T7 transcripts at targeted genome edits. (**A**) RNA yield from IVT on genomic DNA from primary human T cells, measured by UV spectroscopy (A260/A280). Cells were treated with RNP alone (RNP) or with donor and RNP (Donor+RNP). IVT was performed with T7 RNA polymerase (T7 pol) or without polymerase (No pol). (**B–D**) Quantity of IVT transcripts at T7 promoter-labeled genome edits, measured by RT-qPCR and comparative C_t. Target genome locus, guide RNA, and cell type are indicated. Edit-targeted PCR assays are named by their position relative to the edit site (e.g. Edit +109 bp). Endogenous promoter-targeted assays are named by their position relative to promoter orientation (e.g. Chr6 promoter 3'). Reference assays targeted non-coding sequences at three genome loci (*CD28*, *GATA3*, *TRAC*) and two intergenic "genomic safe harbor" sites (GSH1, GHS2)[12]. Results are represented as a difference of C_t values (dC_t) normalized to GSH assays. Bars represent the mean of technical replicates (n = 3), and whiskers represent 2 × SEM.

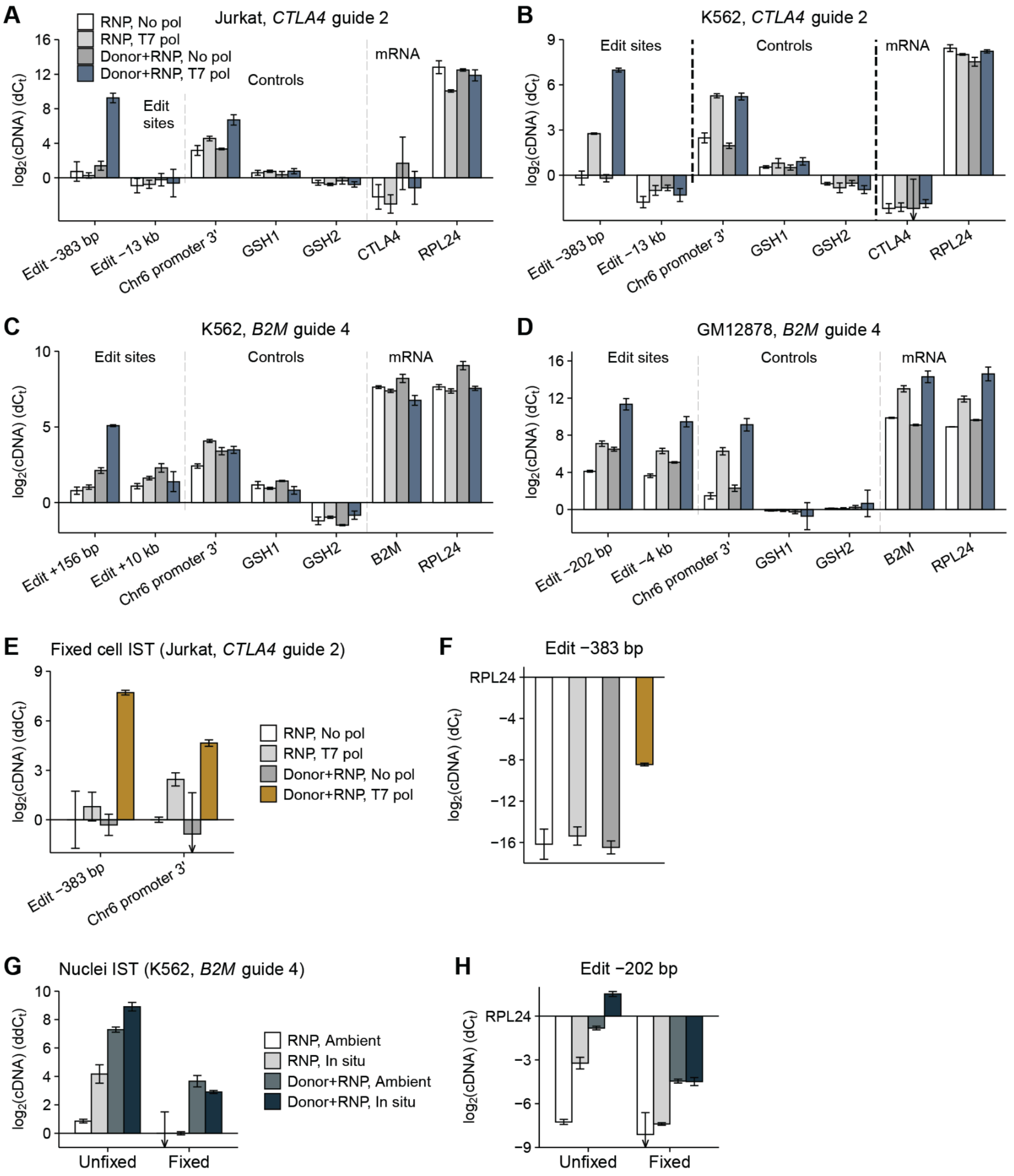

Supplementary Figure 5. Generation of *in situ* T7 transcripts in nuclei and fixed cells. (**A–D**) Quantity of T7 RNA and mRNA in IST reactions on unfixed nuclei, measured by RT-qPCR and comparative C_t. Cell type, target locus, guide RNA, electroporation condition (RNP, Donor+RNP), and polymerase condition (No pol, T7 pol) are indicated. Results are represented as a difference of C_t values (dC_t) normalized to GSH assays. (**E,F**) Quantity of T7 RNA in IST reactions on paraformaldehyde-fixed cells. Results are represented as (**E**) a difference of differences in C_t values (ddC_t) normalized to reference genes *RPL24/RPS10*, and the "RNP, No pol" condition, or (**F**) dC_t relative to *RPL24* gene expression. (**G,H**) Quantity of T7 RNA in IST reactions on unfixed or fixed nuclei from the same isolated nuclei samples. Electroporation condition (RNP or Donor+RNP) and IST fraction (ambient or *in situ*) are indicated. Bars represent the mean of technical replicates (n = 3), and whiskers represent 2 × SEM.

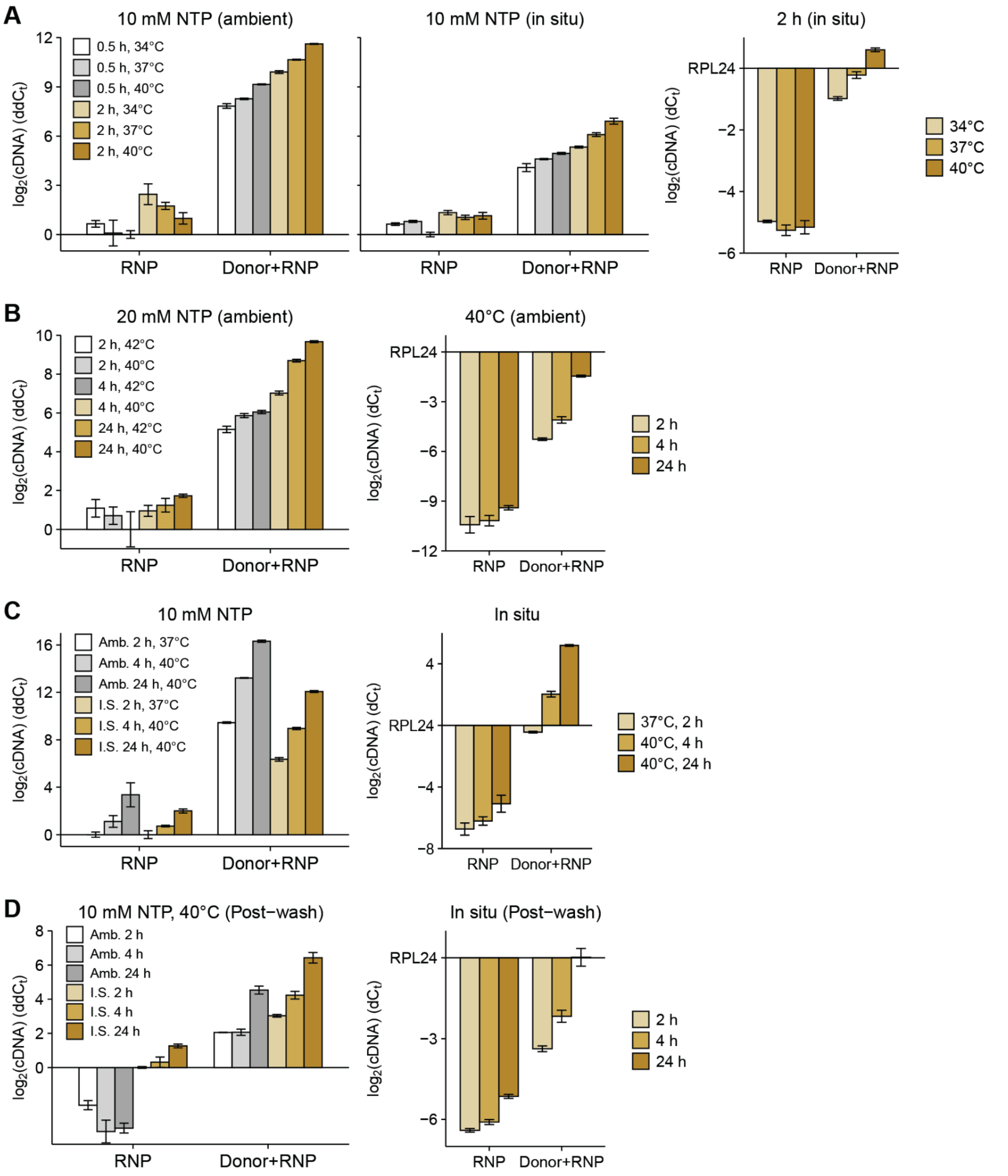

Supplementary Figure 6. Optimization of *in situ* transcription in paraformaldehyde-fixed cells. (**A–D**) Quantity of IST transcripts generated in fixed K562 cells under variable IST reaction conditions (NTP concentration, incubation time, incubation temperature), measured by RT-qPCR and comparative C_t. K562 cells were treated with Cas9 RNP alone (RNP) or with donor DNA (Donor+RNP) using *B2M* guide 4. RT-qPCR was performed on total RNA extracted from the ambient supernatant (Amb.) or *in situ* cell pellet (I.S.) fraction of IST reactions. For **D**, IST cell pellets were washed once by resuspending in SPLiT-seq cell buffer, and total RNA fractions were isolated from this resuspension. Results are represented in two ways. First, as ddC_t normalized to reference genes *RPL24/RPS10*, and a donor-less (RNP) sample. Second, as dC_t normalized to *RPL24* gene expression. All bars represent the mean of technical replicates (*n* = 3), and whiskers represent 2 × SEM.

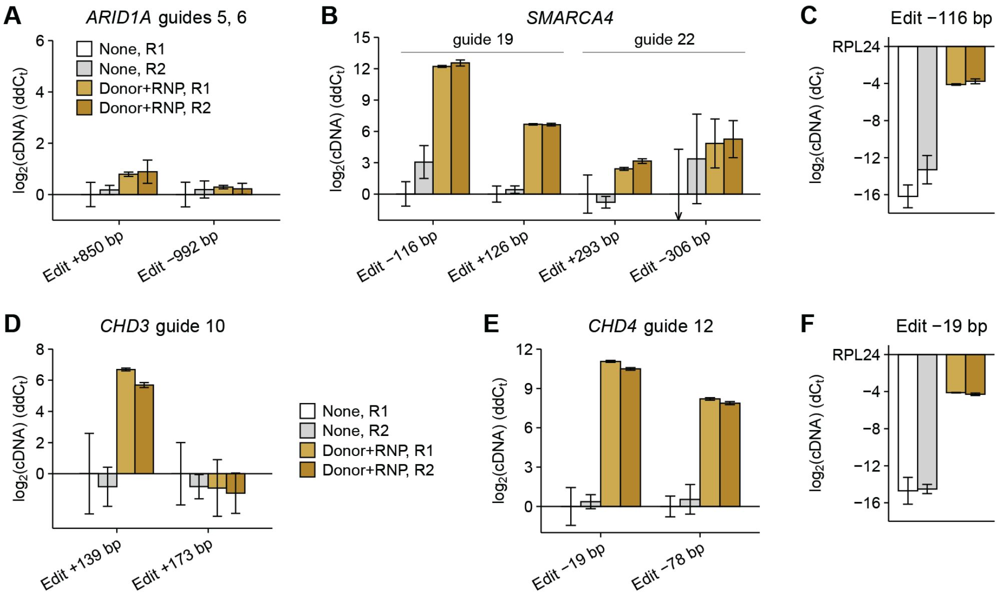

Supplementary Figure 7. *In situ* transcription at Cas9 edits to chromatin remodeler genes. (**A,B**) Quantity of IST transcripts generated in pools of fixed K562 cell lines containing T7 promoter-labeled Cas9 edits in chromatin remodeler genes, measured by RT-qPCR. PCR assays targeted edit sites at (**A**) *ARID1A* or (**B,C**) *SMARCA4* of the BAF complex, and (**D**) *CHD3* or (**E,F**) *CHD4* of the NuRD complex. Three cell pools were used: Mock-electroporated cells (None), *ARID1A*/*SMARCA4*-edited cells (**A–C** Donor+RNP), and CHD3/CHD4-edited cells (**D–F**, donor+RNP). IST replicates (R1, R2) are indicated. Results are represented as (**A,B,D,E**) ddC_t normalized to reference genes *RPL24/RPS10* and a mock-treated sample, or (**C,F**), as dC_t normalized to *RPL24* gene expression. Bars represent the mean of technical replicates (n = 3), and whiskers represent 2 × SEM.

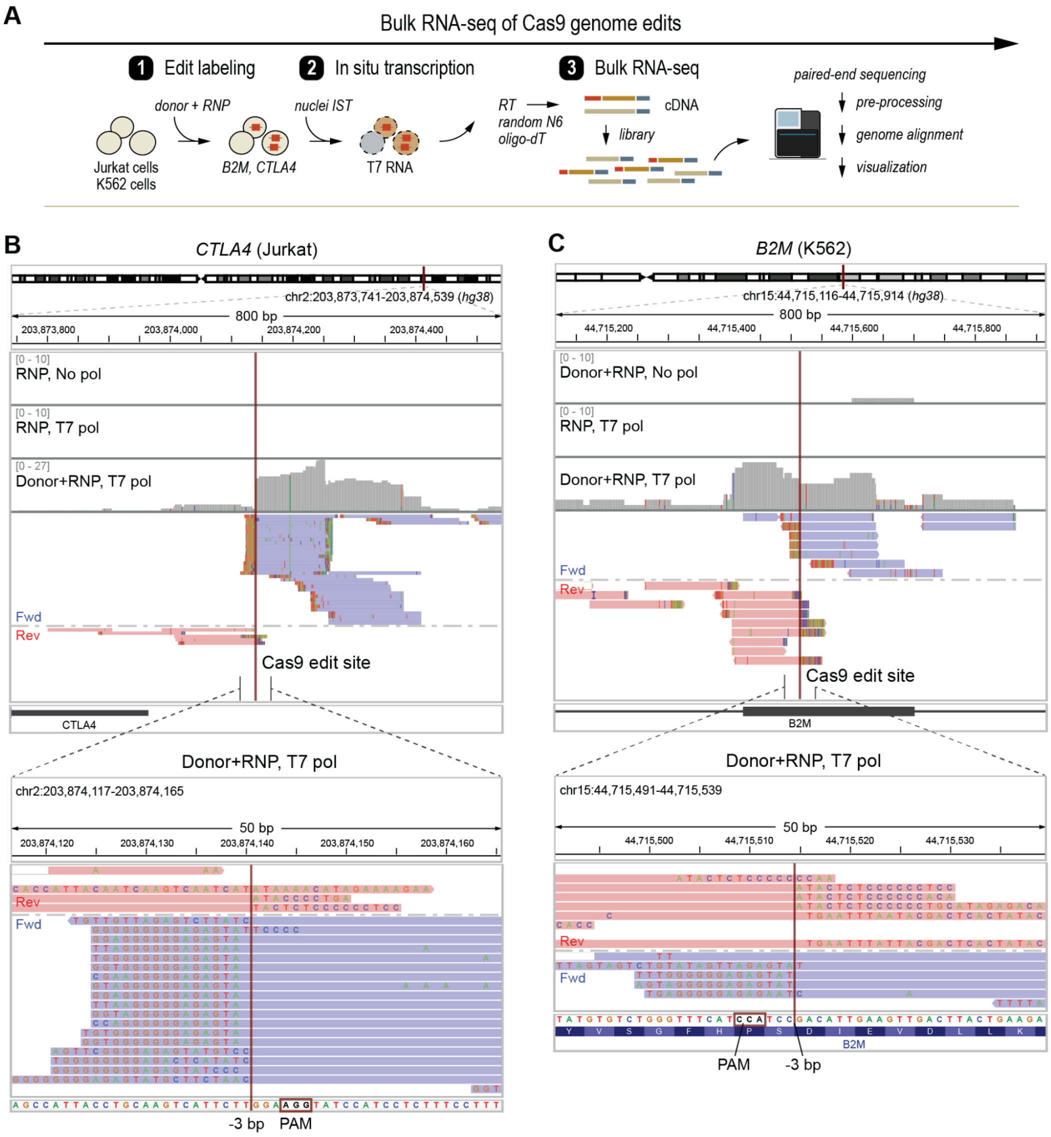

Supplementary Figure 8. Sequencing of *in situ* T7 transcripts identifies Cas9 genome edits. (**A**) Diagram of the bulk RNA-seq experiment. Jurkat and K562 cells were treated with T7 promoter donor DNA and Cas9 RNP targeting *CTLA4* or *B2M*. Nuclei isolation and T7 IST were then performed, followed by total RNA extraction, RNA-seq library preparation, and paired-end sequencing. (**B**,**C**) Sequence alignments at *CTLA4* (**B**) and *B2M* (**C**) for treatments with RNP in the presence or absence of donor DNA (donor+RNP or RNP) and IST reaction in the presence or absence of T7 polymerase (T7 pol or No pol). Read pileups (gray) for all samples, and individual forward/reverse-strand reads (blue/red) for donor+RNP with IST. Targeted PAM, expected Cas9 cleavage position (red line), and unmapped positions (visible bases) are shown.

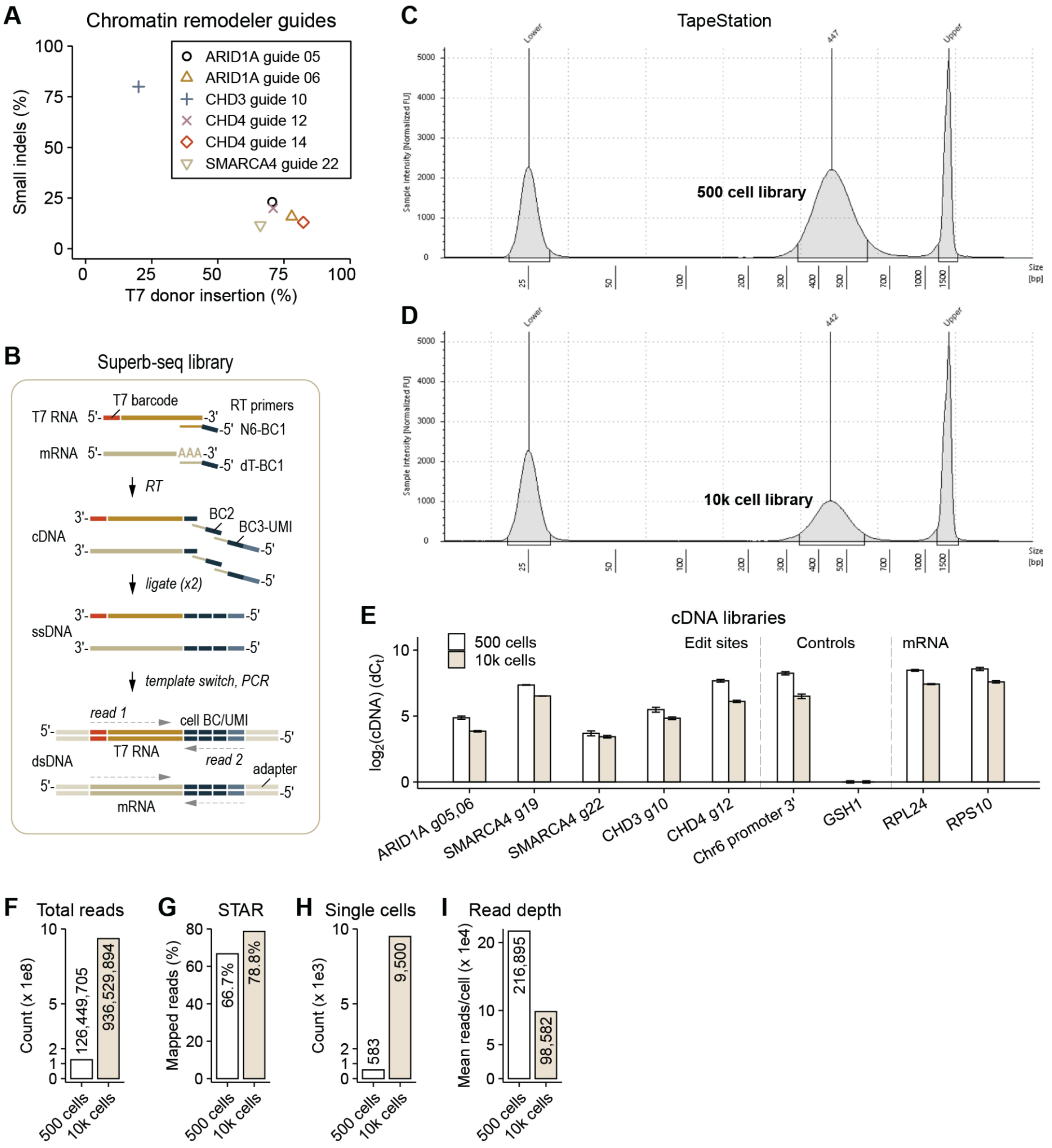

Supplementary Figure 9. Generation of a joint Superb-seq library of T7 and endogenous RNA reads. (**A**) Frequency of donor insertion and donor-less indel events in six K562 cell lines included in the single-cell analysis. (**B**) Combinatorial indexing of T7 and endogenous cDNA fragments with cell barcodes (BC) and unique molecular identifiers (UMI), and final structure of the paired-end Superb-seq library. Detailed sequence structure is shown in **Supp. Fig. 10**. (**C,D**) Distribution of library fragment size in (**C**) the 500 cell library (447 bp) and (**D**) the 10k cell library (442 bp), measured by TapeStation. "Lower" and "Upper" ladders are indicated. (**E**) Quantity of T7 RNA at chromatin remodeler edit sites, T7 RNA at control sites, and mRNA of ribosomal genes in the 500 cell and 10k cell libraries, measured by RT-qPCR and comparative C_t. Control sites are the endogenous T7 promoter-like sequence on chromosome 6 (positive control) and GSH1 (negative control). Bars represent the mean of technical replicates (*n* = 3), and whiskers represent 2 × SEM. (**F**) Total raw read counts for 500 cell and 10k cell libraries. (**G–I**) Summary of Split-pipe read pre-processing and alignment of each library. (**G**) Fraction of reads mapped to human reference genome hg38. (**H**) Number high-quality single cells that were identified. (**I**) Mean read counts per cell (read depth). Additional Split-pipe quality metrics are listed in **Supplementary Table 13**.

A **T7 RNA**

```
     READ 1|------------------->
            T7 barcode  genome
5'-[TruSeq]-GGGAGAGTAT-XXXX…XXXX-NNNNNN-XXXXXXXX-[L1]-XXXXXXXX-[L2]-XXXXXXXX-NNNNNNNNNN-[TruSeq]-3'
3'-[TruSeq]-CCCTCTCATA-XXXX…XXXX-NNNNNN-XXXXXXXX-[L1]-XXXXXXXX-[L2]-XXXXXXXX-NNNNNNNNNN-[TruSeq]-5'
                                 N6-RT  Cell BC1      Cell BC2      Cell BC3    UMI
                                        <-----------------------------------------------|READ 2
  | 70 bp  | 10 bp    | ~200 bp | 6 bp | 8 bp   | 22 | 8 bp   | 30 | 8 bp   | 10 bp    | 66 bp  |
```

B **Cell mRNA**

```
     READ 1|--------------------------->
              mRNA          polyA
5'-[TruSeq]-XXXX…XXXX-NVAAAAAAAAAAAAAAA-XXXXXXXX-[L1]-XXXXXXXX-[L2]-XXXXXXXX-NNNNNNNNNN-[TruSeq]-3'
3'-[TruSeq]-XXXX…XXXX-NVTTTTTTTTTTTTTTT-XXXXXXXX-[L1]-XXXXXXXX-[L2]-XXXXXXXX-NNNNNNNNNN-[TruSeq]-5'
                            dT-RT       Cell BC1      Cell BC2      Cell BC3    UMI
                                        <-----------------------------------------------|READ 2
  | 70 bp  | ~200 bp | 17 bp           | 8 bp   | 22 | 8 bp   | 30 | 8 bp   | 10 bp    | 66 bp  |
```

```
        Key                                             Linkers
        BC1-3  = Cell/sample barcodes                   L1  5'-CCACAGTCTCAAGCACGTGGAT-3'
        UMI    = Unique molecular identifier                5'-GGTGTCAGAGTTCGTGCACCTA-3'
        N6-RT  = Random hexamer RT primer
        dT-RT  = Oligo-dT RT primer                     L2  5'-AGTCGTACGCCGATGCGAAACATCGGCCAC-3'
        TruSeq = Dual index adapters                        3'-TCAGCATGCGGCTACGCTTTGTAGCCGGTG-5'
```

C **Adapters**

```
                                                                 READ 1|----->
   P5 adapter                               TruSeq adapter          R1 primer
5'-AATGATACGGCGACCACCGAGATCTACAC-NNNNNNNN-ACACTCTTTCCCTACACGACGCTCTTCCGATCT-[cDNA]…
3'-TTACTATGCCGCTGGTGGCTCTAGATGTG-NNNNNNNN-TGTGAGAAAGGGATGTGCTGCGAGAAGGCTAGA-[cDNA]…
                                     i5    i5 primer
                                  <-------|INDEX 2
  | 29 bp                            | 8 bp   | 33 bp                          |~200 bp|
```

```
                                                             INDEX 1|------->
                                       TruSeq adapter          i7 primer    i7
                             …[BC-UMI]-AGATCGGAAGAGCACACGTCTGAACTCCAGTCAC-NNNNNNNN-ATCTCGTATGCCGTCTTCTGCTTG-3'
                             …[BC-UMI]-TCTAGCCTTCTCGTGTGCAGACTTGAGGTCAGTG-NNNNNNNN-TAGAGCATACGGCAGAAGACGAAC-5'
                                       R2 primer                                                    P7 adapter
                              <-------|READ 2
                             | 86 bp  | 34 bp                          | 8 bp   | 24 bp                    |
```

Supplementary Figure 10. Sequence structure of Superb-seq paired-end library fragments. (**A**) T7 RNA fragments. Read 1 contains the 5' T7 barcode and genome sequence at the Cas9 edit site. Read 2 contains the UMI and SPLiT-seq three-part combinatorial cell barcode (Evercode v2, Parse Biosciences). Cell barcode 1 (BC1) is the sample barcode. (**B**) Cell mRNA fragments. Read 1 contains mRNA sequence. These are representative fragments, as both T7 RNA and mRNA can be captured by both types of RT primers. (**C**) Structure of the dual-index sequencing adapters (TruSeq, Illumina). Sequences were assembled as best as possible from kit documentation and the Single Cell Genomics Library Structure resource (https://teichlab.github.io/scg_lib_structs/methods_html/SPLiT-seq.html)[13].

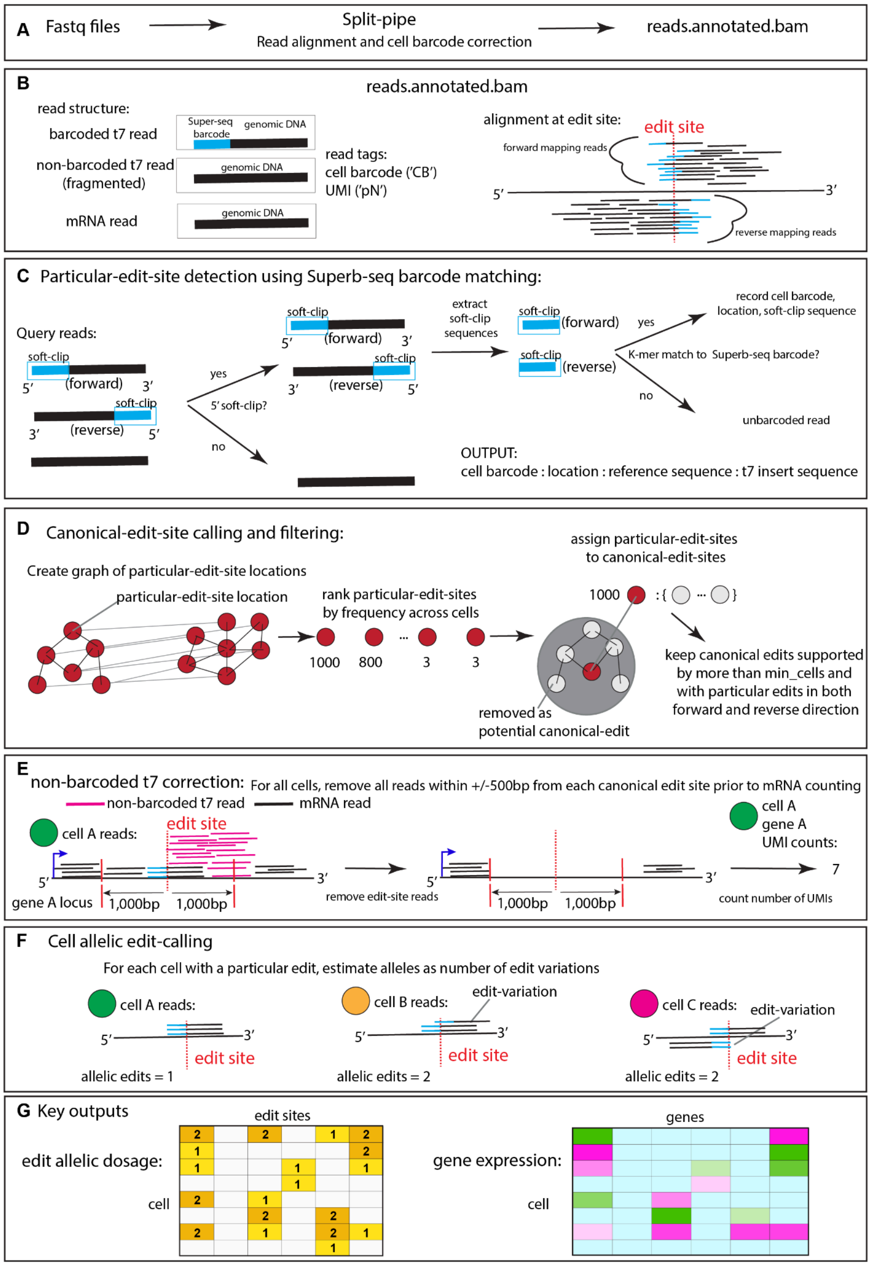

Supplementary Figure 11. Sheriff, a command line tool to quantify Superb-seq edits and transcriptomes. (**A**) Fastq files generated from Superb-seq are processed using Split-pipe to generate an annotated bam file. (**B**) Three kinds of reads are present within the annotated bam file; 1) T7 polymerase generated reads with a Superb-seq barcode, evident in the 5' soft-clipped region of the mapped read, 2) reads generated by T7 polymerase that have been fragmented, and hence without Superb-seq barcode, and 3) reads generated from normal RNA pol II transcription within expressed genic regions. Edit sites have a distinct T7 barcoded read pattern. (**C**) Particular-edit-sites are determined by using cell barcodes on classified T7 barcoded reads, identified by a *k*-mer match on the 5’ soft-clip sequence of reads. (**D**) Particular-edits represent slight edit variation around an edit site. Canonical-edit-sites are determined by assigning particular-edits to more commonly occurring versions of the edit, by first constructing a graph of particular-edit locations for each chromosome, and then rank-ordering particular-edits by the frequency that they occur across cells, then progressively assigning particular-edits to canonical-edit-sites. (**E**) All remaining reads within ± 1000 bp from canonical-edit-sites are removed prior to cell/gene UMI counting, since otherwise non-barcoded T7 reads will confound gene expression estimation. (**F**) For each canonical-edit-site, the number of edited alleles for that site is called per cell. (**G**) Key outputs from Superb-seq read processing. See **Methods** for details.

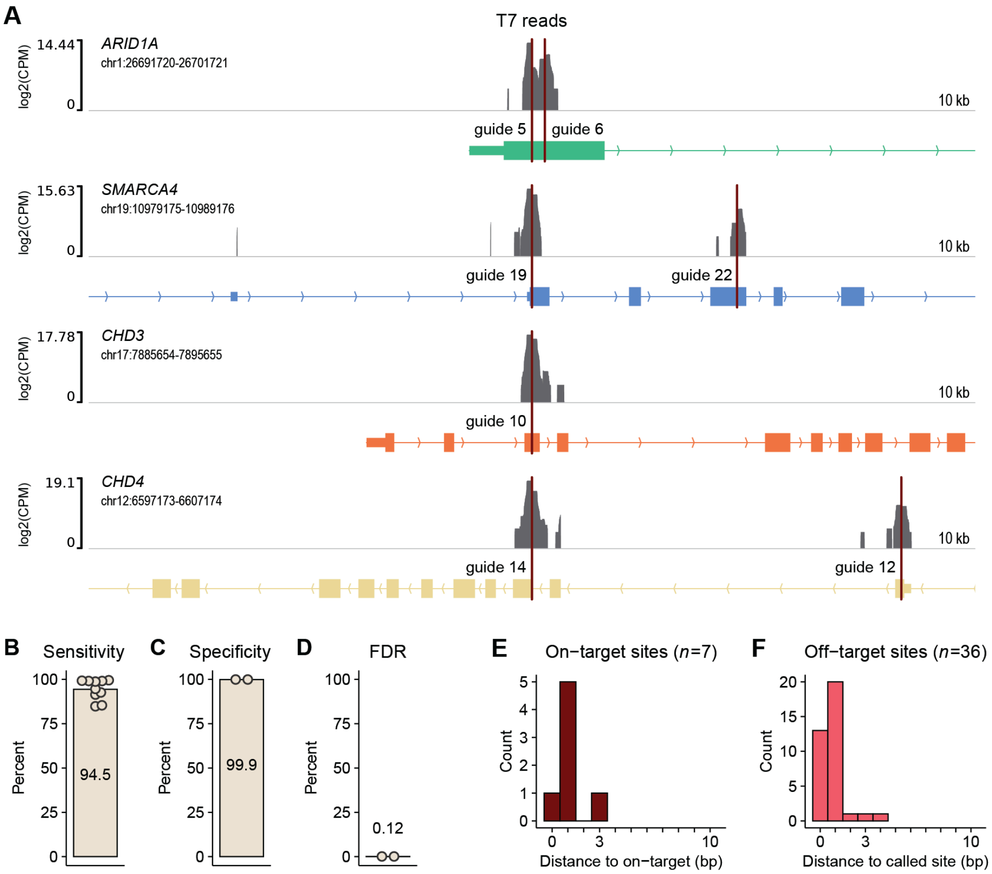

Supplementary Figure 12. Performance of T7 read identification by Sheriff. (**A**) Coverage of T7 reads called by Sheriff at the seven on-target edit sites (count per million mapped reads, CPM). Expected on-target sites are indicated for each guide RNA (red lines). (**B**) Sensitivity of T7 read calling in 10 technical cell barcoding replicates of BAF- or NuRD-edited sample pools. Sensitivity was defined as the rate of correct T7 read calls among total reads within 100 bp of the seven expected on-target edit sites. (**C**) Specificity and (**D**) False discovery rate (FDR) of T7 read calling in two technical replicates of the unedited sample pool. Specificity was defined as the rate of correct non-T7 read calls in the unedited sample. FDR was defined as the rate of non-T7 reads among called T7 reads. (**E**) Absolute distance between expected on-target edit sites and called edit sites by Sheriff. (**F**) Absolute distance between called edit sites and guide-targeted PAM sequences for the 36 off-target sites, determined by Sheriff.

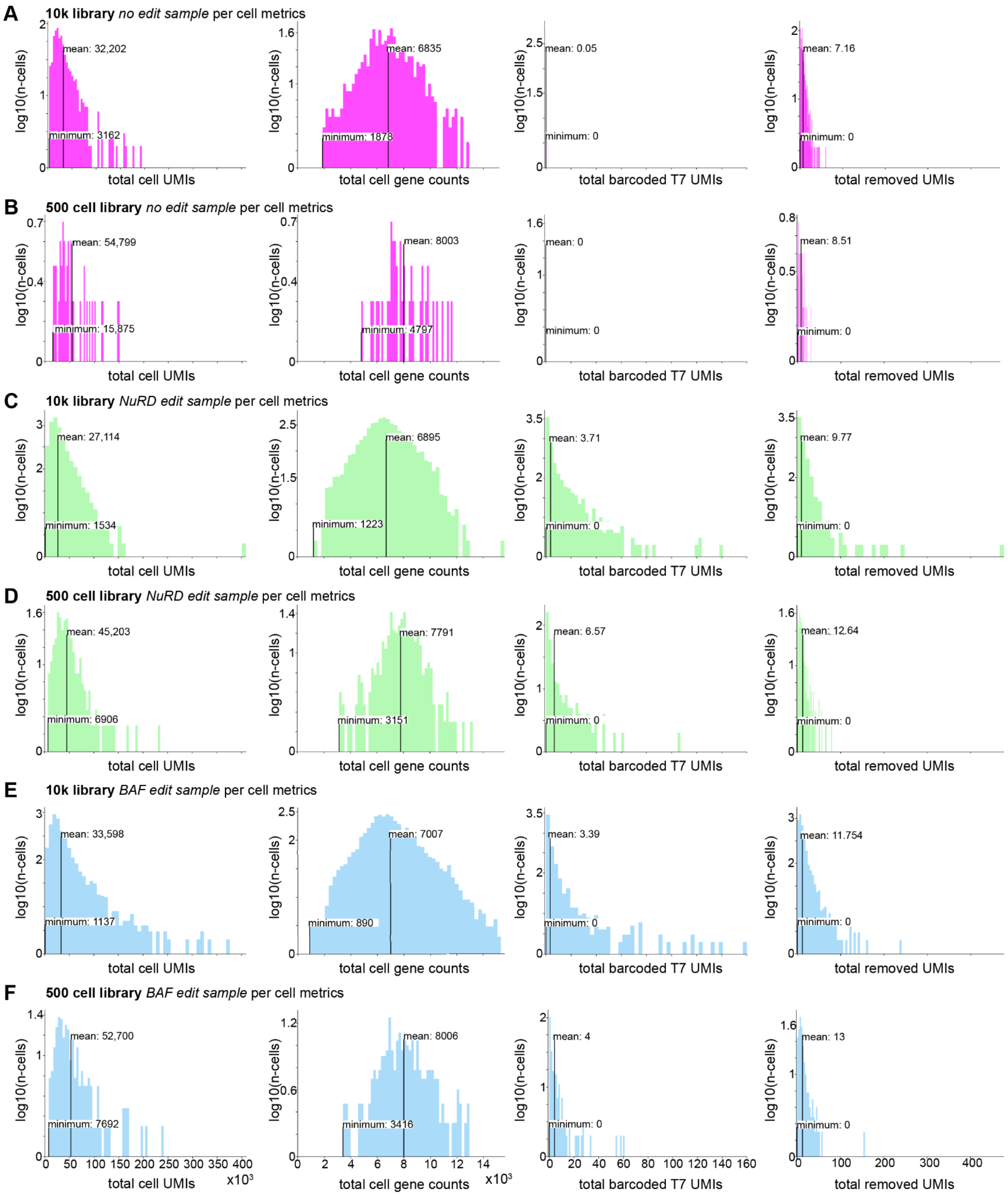

Supplementary Figure 13. UMI and gene count distributions across Superb-seq sample pools. (**A**) 10k cell library no-edit sample (*n* = 684 cells) histograms of single-cell quality control metrics including total UMIs, total genes detected, total barcoded T7 UMIs detected, and total removed T7 UMIs occurring ± 1000 bp from detected edit sites. (**B**) 500 cell library quality control metrics for no edit cells (*n* = 41). (**C**) 10k cell library NuRD edit sample quality control metrics (*n* = 5086 cells treated with RNPs targeting *CHD3*/*CHD4* loci). (**D**) 500 cell library quality control metrics for NuRD edit sample (*n* = 323 cells). (**E**) 10k cell library quality metrics for BAF edit sample (*n* = 3720 cells treated with RNPs targeting *ARID1A*/*SMARCA4* loci). (**F**) 500 cell library quality metrics for BAF edit sample (*n* = 219 cells).

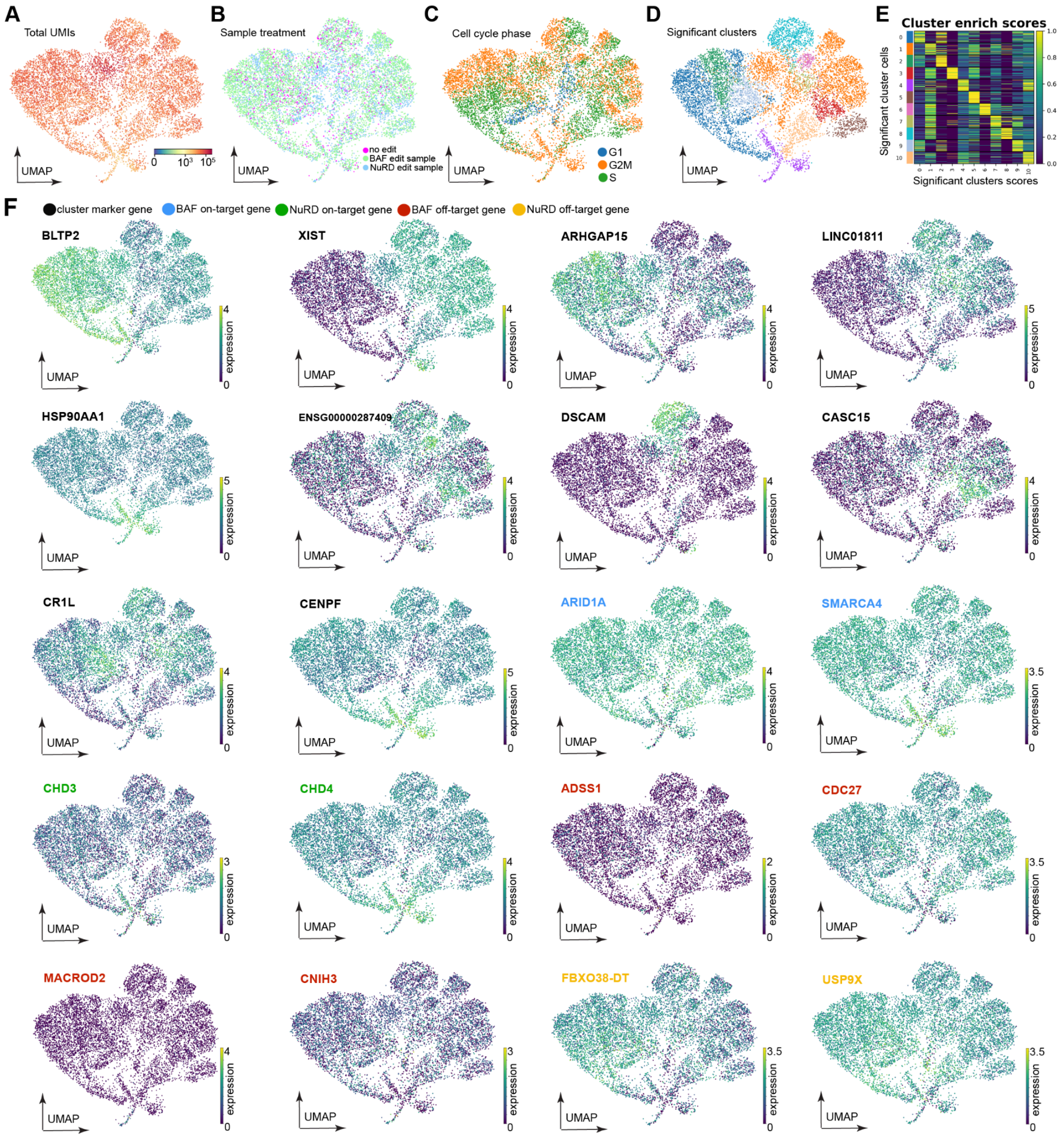

Supplementary Figure 14. Transcriptional heterogeneity of Superb-seq single-cell gene expression. (**A**) Uniform manifold approximation (UMAP) of Superb-seq single-cell gene expression with total cell UMIs annotated. (**B**) UMAP with cell edit sample treatments annotated (no edit sample, BAF edit sample, NuRD edit sample). (**C**) UMAP with predicted cell cycle phase annotations. (**D**) UMAP with significantly different single-cell clusters annotated. (**E**) Heatmap with cells on the rows grouped by significant clusters, and significant clusters on the columns. Higher values indicate higher expression of the significant clusters marker genes in the single cells, showing distinct gene expression between the single-cell clusters. (**F**) UMAPs with gene expression annotated. Genes displayed are either marker genes of distinctly different clusters of cells, BAF editing on-target genes (*ARID1A*/*SMARCA4*), NuRD editing sample on-target genes (*CHD3*/*CHD4*), or off-target edited genes (*ADSS1*, *CDC27*, *MACROD2*, *CNIH3*, *FBXO38-DT*, *USP9X*).

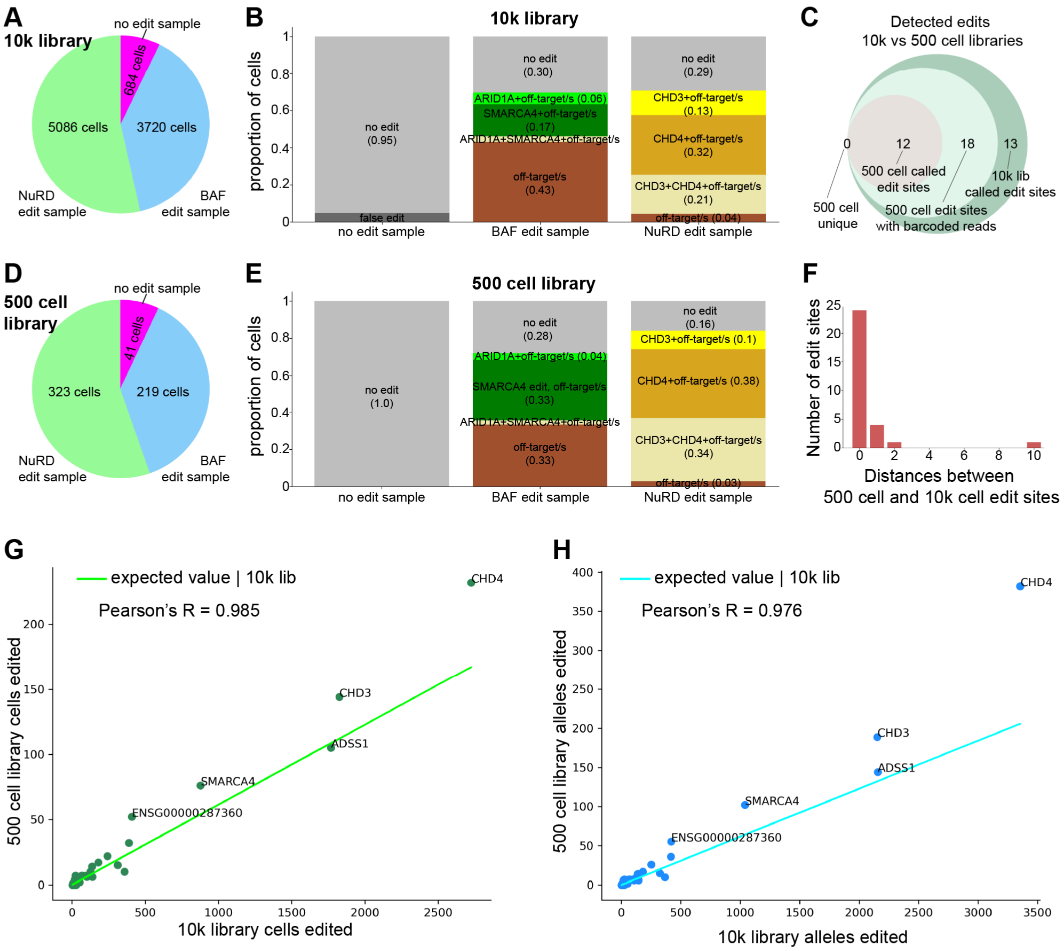

Supplementary Figure 15. Reproducible edit capture in 500 cell and 10k cell libraries. (**A**) Proportion of cells within each edit sample for the 10k cell library. (**B**) Proportion of cells within each edit sample, stratified by no detected cas9 editing (no edit), cells with one or more off-target edits but no on-target edits (off-target/s), cells that were double-edited for both on-target genes in the edit sample (doublet-edit), or cells that had one of the on-target genes edited (*SMARCA4* or *ARID1A* for BAF edited sample, *CHD3* or *CHD4* for NuRD edited sample). (**C**) Venn diagram indicating the intersection of Cas9 edits detected in the 500 cell library and the 10k cell library. (**D,E**) Equivalent to **A,B** but for the 500 cell library. (**F**) Bar-chart indicating the distance between equivalent edit sites detected in both Superb-seq libraries. (**G**) Scatter plot indicating the number of cells edited for each edit site in the 10k cell library on the x-axis and the 500 cell library on the y-axis. The plotted line indicates the expected number of cells edited in the 500 cell library given the observed proportion of cells edited in the 10k cell library. (**H**) The equivalent to G, except with respect to the total number of detected alleles edited at each edit site within each respective library.

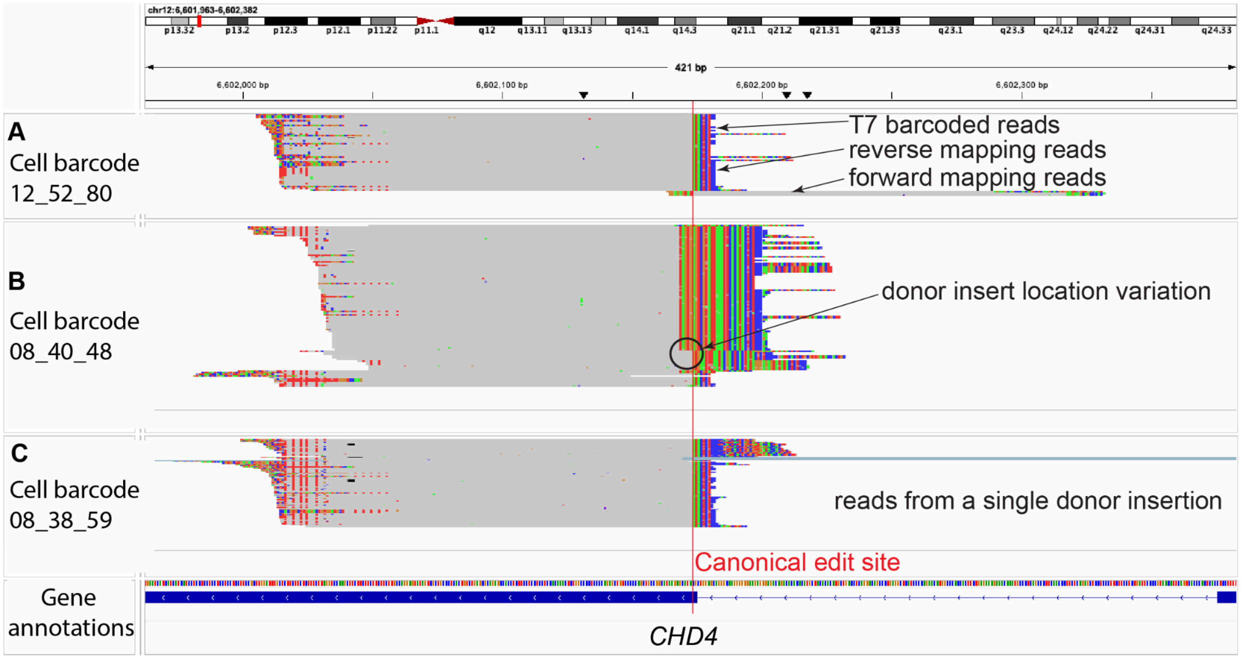

Supplementary Figure 16. T7 read features indicate multiple edit alleles within individual cells. Integrative Genomics Viewer (IGV) snapshot of T7 barcoded reads from the Superb-seq 500 cell library, with soft-clipped sequences and base mismatches to the reference genome visualised. The *CHD4* on-target edit site is at the locus centre, with the called canonical edit site indicated with a vertical line. (**A**) T7 barcoded reads called in the cell 12_52_80, which had both forward and reverse mapping T7 reads, mutually exclusive events that indicate more than one allele has the Superb-seq donor insertion. (**B**) T7 barcoded reads for cell 08_40_48. Differences in barcoded T7 read mapping position indicating variation in the donor sequence insertion site indicating more than one allele is edited in this cell. (**C**) T7 barcoded reads in cell 08_38_59, with no clear variation in donor sequence insert, indicating only 1 edited allele.

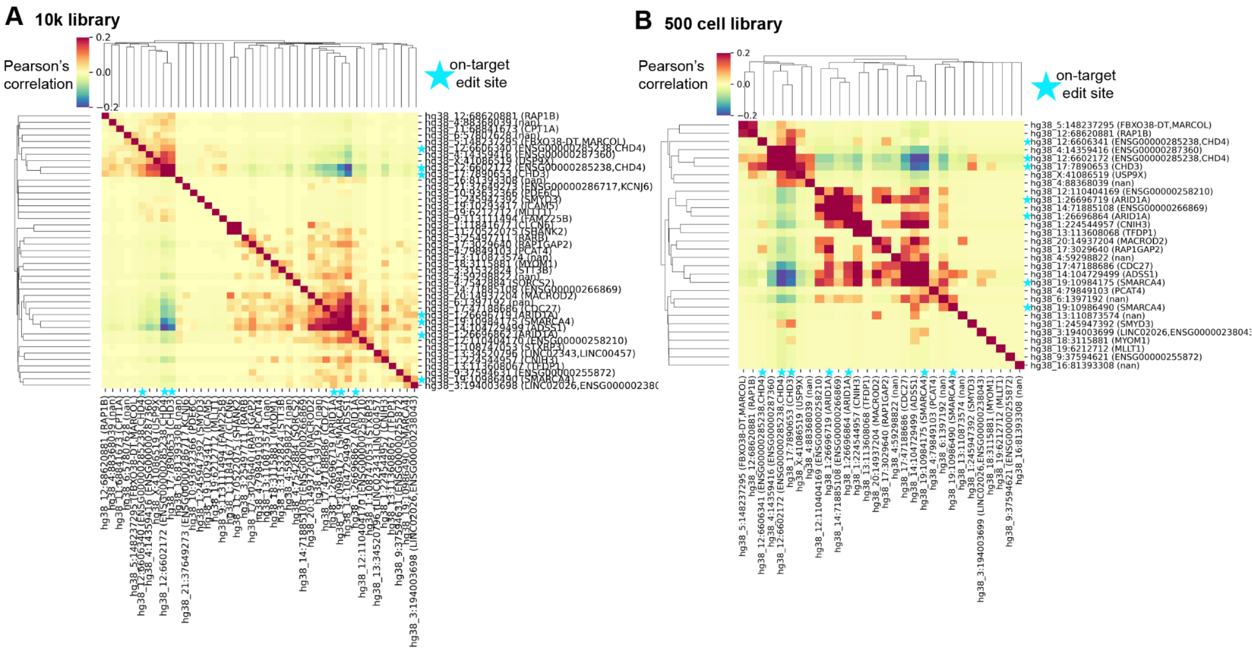

Supplementary Figure 17. Co-occurrence of on- and off-target Cas9 edit alleles within individual cells. (**A**) Heatmap with cluster dendrograms of pairwise Pearson correlations of edit allele dosages across cells in the 10k Superb-seq library. Each row and column is a called edit site, and values indicate the correlation of edit allele dosages across cells for the compared edit sites. On-target edit sites corresponding to the 7 guides are highlighted with stars. (**B**) Equivalent to A except for the 500 cell library.

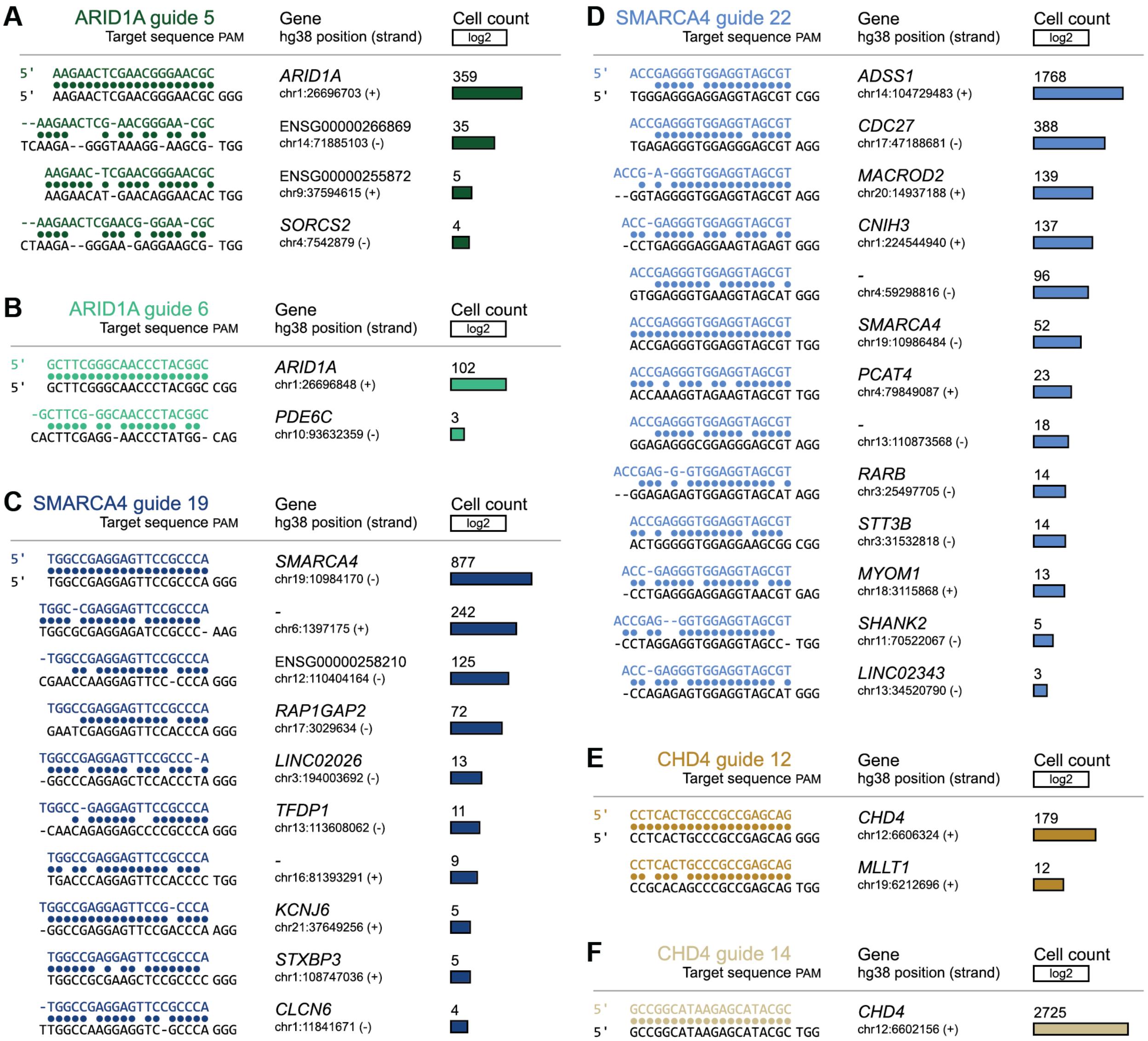

Supplementary Figure 18. Sequence similarity identifies off-target causal guide RNAs. (**A–F**) Aligned guide and target sequences of the additional six of seven guide RNAs. Matches (●) and gaps (-) are indicated, along with the intersecting gene if present, cell count, start position, and strand.

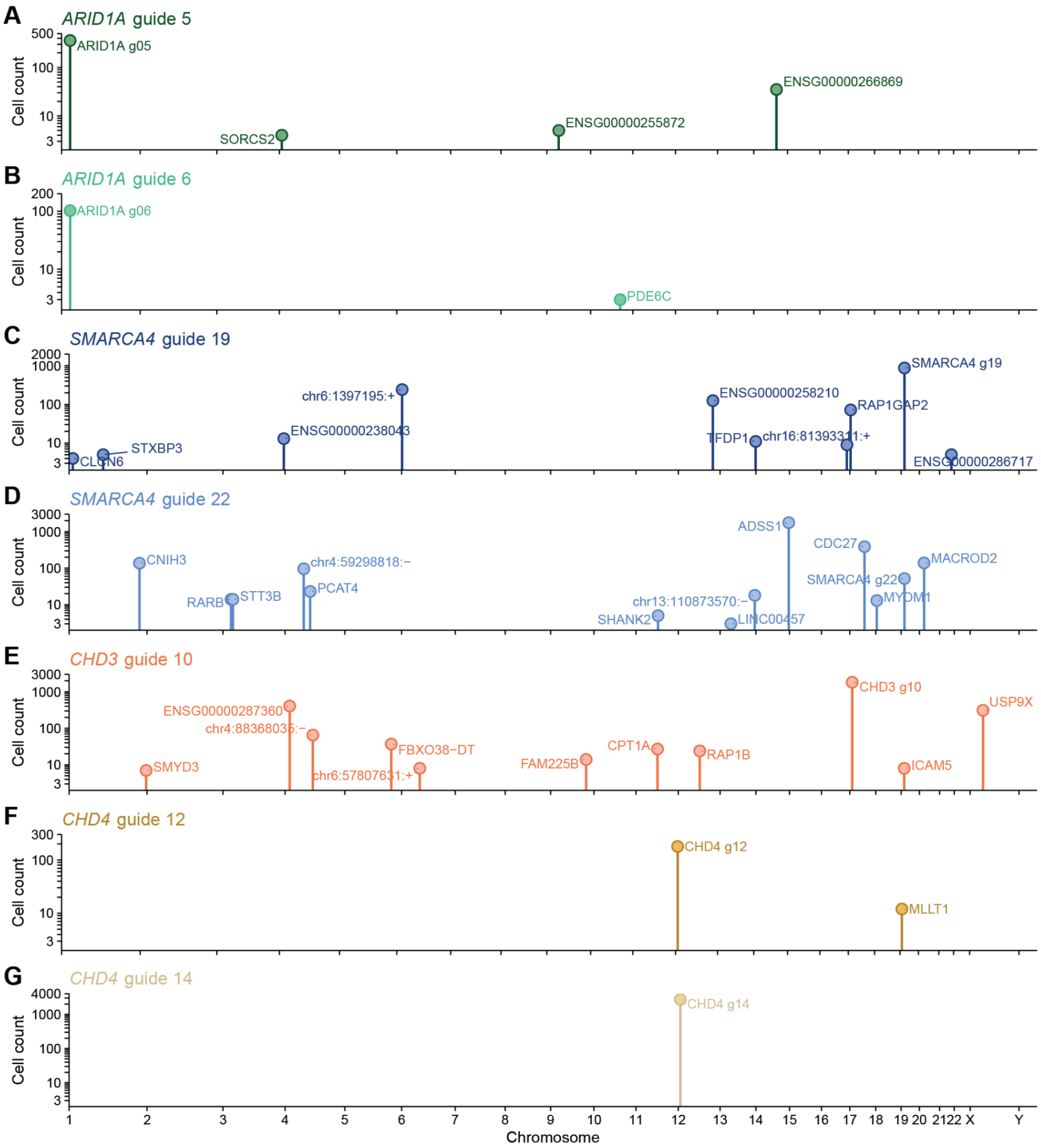

Supplementary Figure 19. Superb-seq captures guide-specific editing profiles from pooled samples. (A–G) On-target and off-target Cas9 edit sites identified in the 10k cell library, separated by causal guide RNA. Genome positions (hg38) and cell counts are indicated.

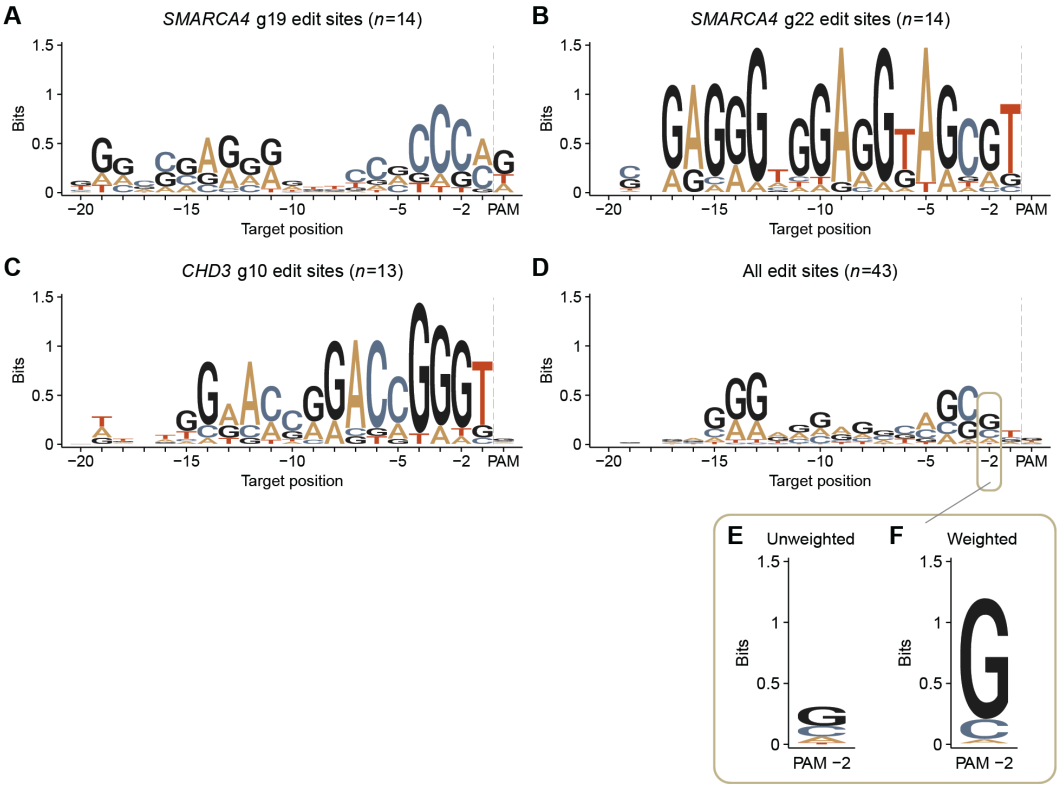

Supplementary Figure 20. Sequence similarity between off-target sequences. (**A–F**) Positional Shannon entropy (bits) of on- and off-target sequences associated with (**A–C**) single guide RNAs with ≥ 10 total edit sites, or (**D**) all seven guides, calculated using ggseqlogo[11]. Total number of target sequences (*n*) are indicated. (**B**) Shannon entropy of the PAM -2 position across 43 on-target and off-target sequences, either unweighted or weighted by the observed cell count of each target sequence.

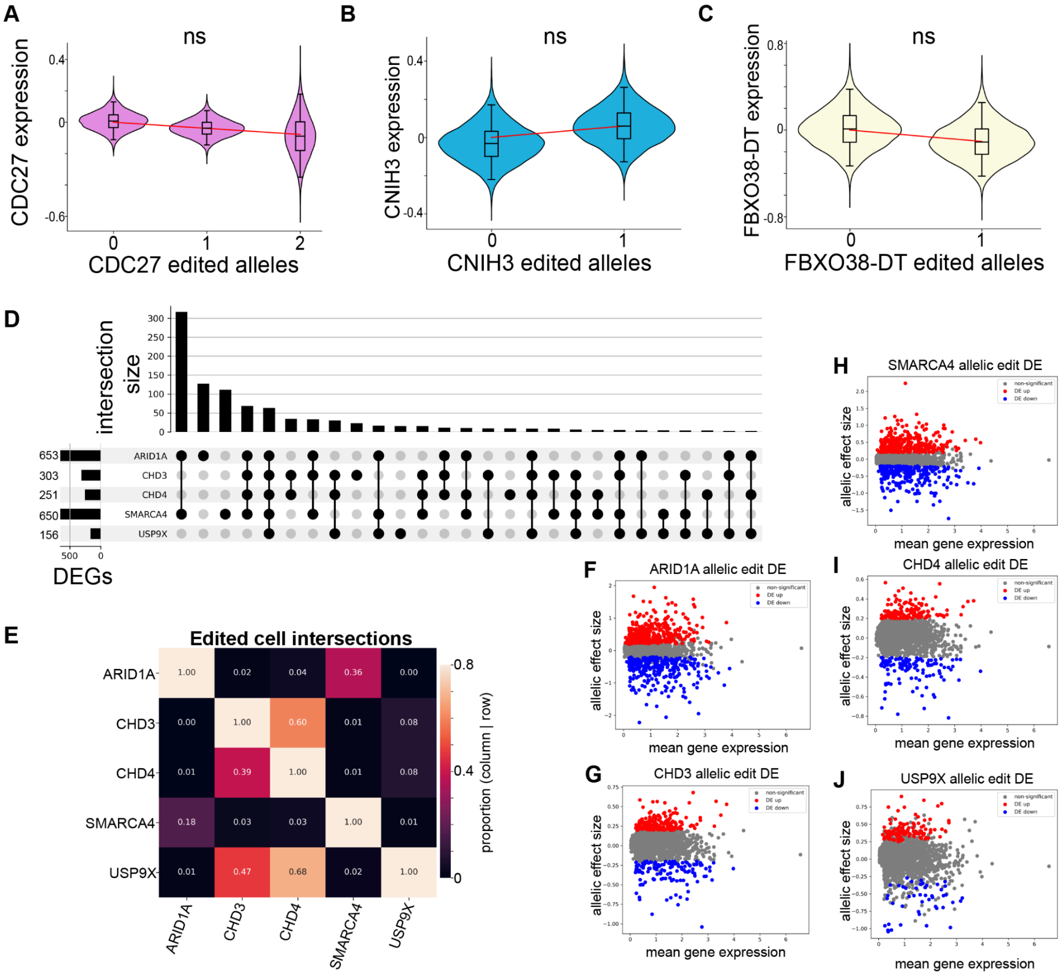

Supplementary Figure 21. Differential gene expression associates with Cas9 edit allele dosage. (**A–C**) Violin plots of corrected gene expression estimates from n = 10,000 bootstrap iterations, grouped by edit allele dosage for off-target edits within the gene body of the tested gene. Overlaid box-plots show the interquartile range with the whiskers indicating the full data range (minimum and maximum). Lines indicate the effect on expression of edit allele dosage estimated by the linear mixed model. P-values are from performing a two-sided Wald test using the linear mixed model. (**D**) Upset plot indicating the intersection sizes of DEGs associated with the on- and off-target edit. (**E**) Heatmap indicating the proportion of cells within combinations of selected Cas9 edits. Proportions are conditioned on the edited gene listed on each row. For example, row one of the heatmap indicates 36% of cells with *ARID1A* edits also have *SMARCA4* edits. (**F–J**) Scatter plots with allelic effect size on the y-axis and mean gene expression on the x-axis, indicating differentially expressed genes are detected across a range of gene expression levels without bias based on mean expression level.

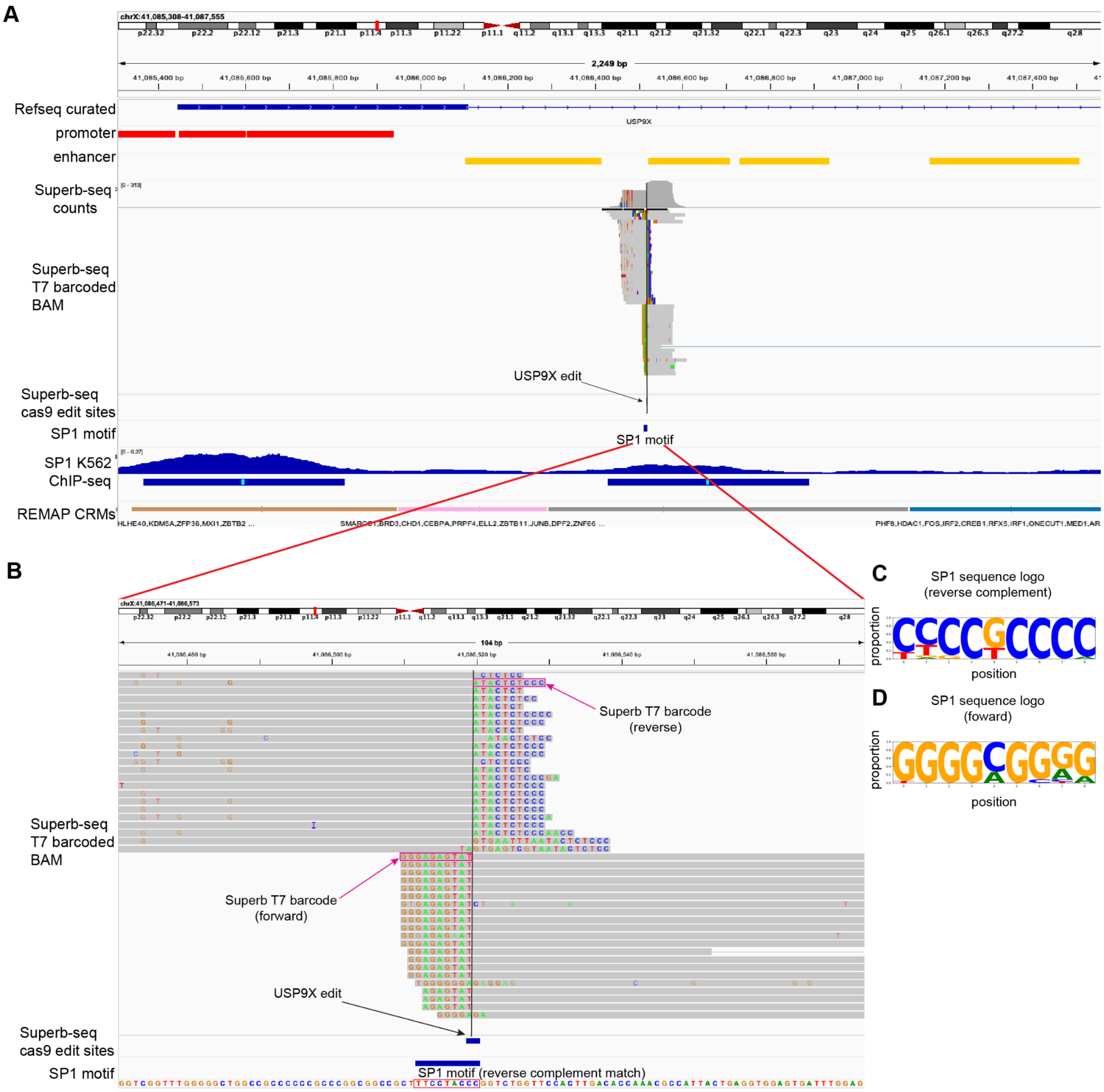

Supplementary Figure 22. Off-target USP9X edit site intersects an SP1 binding motif. (**A**) Integrative Genome Viewer (IGV) track view showing (from top to bottom) Refseq gene annotations, ENCODE promoter annotations, ENCODE candidate enhancer annotations, Superb-seq T7 barcoded read count pile-ups, Superb-seq T7 reads BAM (soft-clipped mismatched base pairs due to Superb barcode are highlighted either side of the *USP9X* Cas9 edit site), Superb-seq canonical edit site locations, annotation of SP1 motif that intersects the *USP9X* edit site, SP1 K562 ChIP-seq with fold-change over control and IDR conserved peaks annotated, and REMAP cis regulatory module (CRM) annotations. (**B**) Zoomed in view of A, except only showing the Superb-seq T7 barcoded read BAM with the Superb barcoded highlighted, the annotated canonical edit site for *USP9X*, and the identified SP1 motif with the reference sequence highlighted (corresponding to the reverse complement of the actual SP1 motif). (**C**) Sequence logo of reverse complement of SP1 motif. (**D**) Sequence logo for the forward strand version of the SP1 motif.

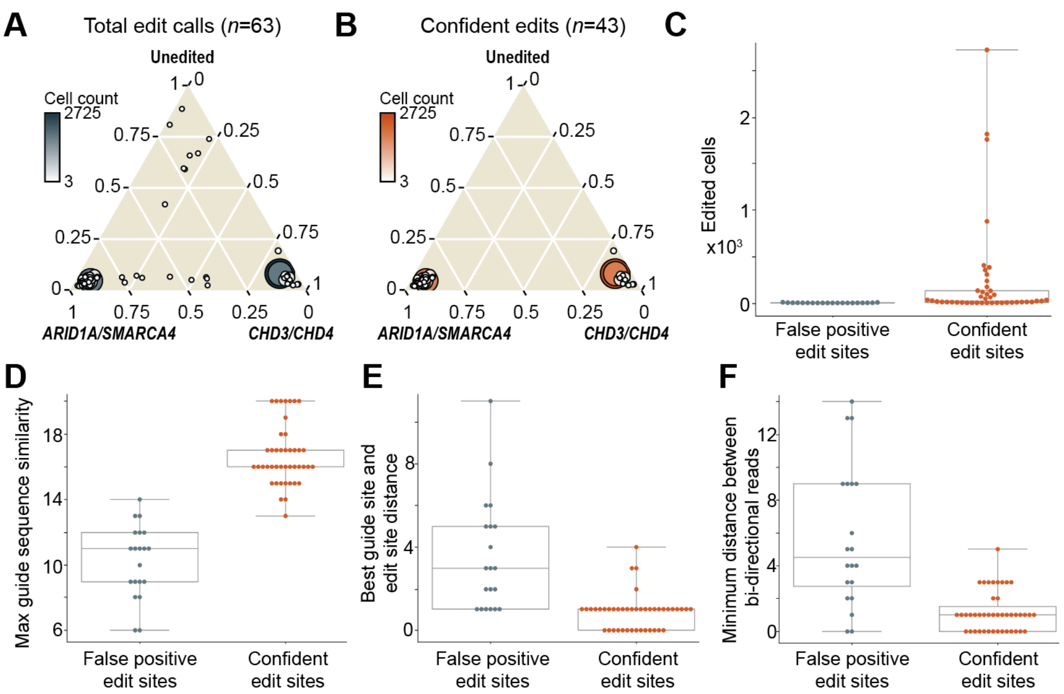

Supplementary Figure 23. Multiple independent metrics confirm confident Cas9 edit sites. (**A**) Ternary plot, with each dot indicating a called canonical edit site (total *n* = 63). Dots are coloured and scaled by the number of cells called with T7 barcoded reads for the edit site. Edit sites are plotted according to the proportion of cells called with the edit in the different treatment samples (unedited, *ARID1A*/*SMARCA4* guide treated cells, *CHD3*/*CHD4* guide treated cells). True edit sites are expected to be specific to a particular treatment sample. (**B**) Equivalent to A, except subsetted to confident edit sites, called based on manual examination of the T7 barcoded read alignment at each edit site. (**C**) Swarm- and box- plots overlaid, with each point indicating a called edit site, with edit sites stratified into confident and false positive edit sites on the x-axis. Overlaid box-plots show the interquartile range with the whiskers indicating the full data range (minimum and maximum). The y-axis indicates the number of cells within which the respective edit was detected. (**D**) Equivalent to C, except with the y-axis indicating the maximum sequence similarity across the 7 guide sequences and the reference genome at the called edit sites. (**E**) Equivalent to C, except the y-axis indicates the distance between the best candidate Cas9 cleavage site based on sequence similarity to one of the 7 guide sequences, and the called canonical edit site position. (**F**) Equivalent to C, except the y-axis indicates the minimum base pair distance between the closest pair of forward and reverse mapping reads, with true edit sites expected to have bi-directional reads immediately either side of the Cas9 cleavage site for true edit events.

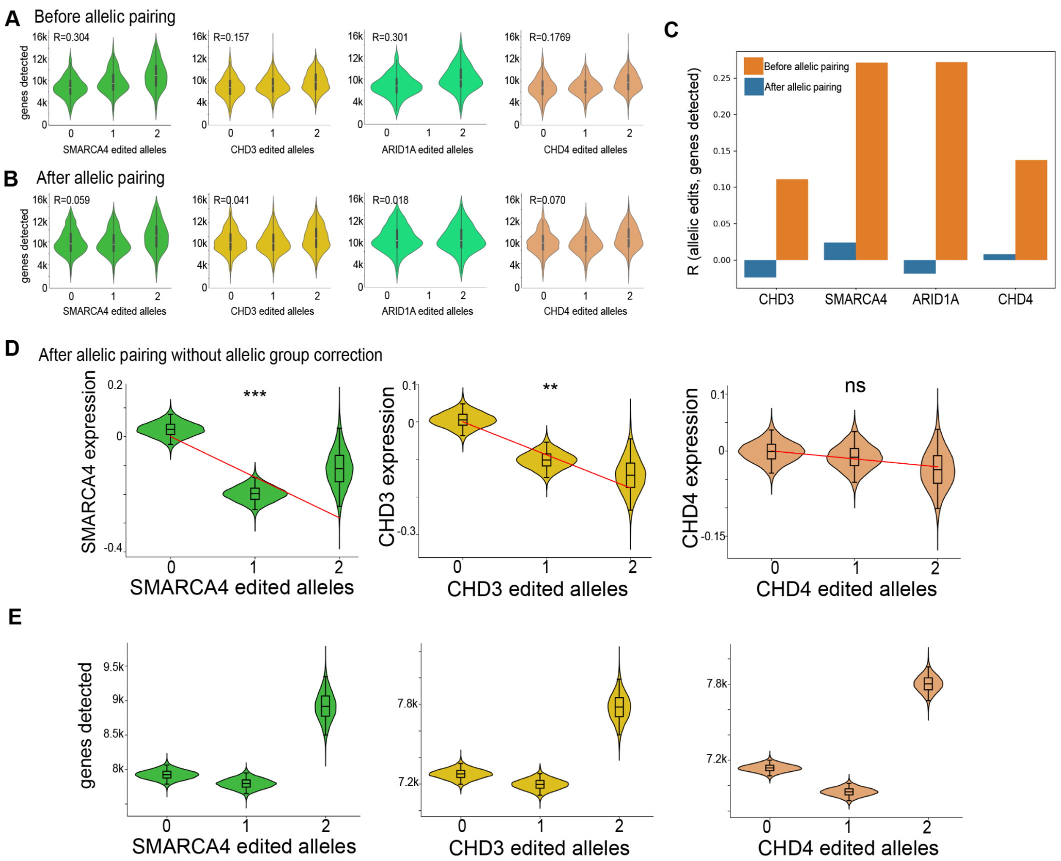

Supplementary Figure 24. Correction of gene expression estimates by allelic pairing. (**A**) Correlation of the number of detected genes per cell with the number of detected edit alleles per cell at the indicated on-target gene (*SMARCA4*, *CHD3*, *ARID1A*, *CHD4*). Pearson correlation (R) is indicated. Overlaid box-plots show the interquartile range (IQR) with the whiskers indicating the full data range (minimum and maximum), excluding outliers that fall outside 1.5x the IQR above or below the 25th and 75th percentiles. (**B**) Equivalent to A after performing allelic pairing. (**C**) Pearson correlations between the gene detection rate and the edit alleles detected. Correlations are stratified by the edited gene and before or after performing the allelic pairing. (**D**) Violin plots with boot-strapped mean estimates (10,000 boot-straps) of expression for genes fitted with a linear mixed model that does not consider allelic groups (groups of unedited and 1 allele cells, unedited and 2 allele cells) when performing the linear mixed modeling. **Fig. 5B** has the version of this panel with the allelic group correction. (**E**) Violin plot with boot-strapped mean estimates (10,000 boot-straps) of the genes detected per edit allele call after allelic pairing strategy. Overlaid box-plots show the interquartile range with the whiskers indicating the full data range (minimum and maximum).

Supplementary references

1. Shou, J., Li, J., Liu, Y. & Wu, Q. Precise and predictable CRISPR chromosomal rearrangements reveal principles of Cas9-mediated nucleotide insertion. *Mol. Cell* **71**, 498–509.e4 (2018).
2. Wang, T., Wei, J. J., Sabatini, D. M. & Lander, E. S. Genetic screens in human cells using the CRISPR-Cas9 system. *Science* **343**, 80–84 (2014).
3. Xu, H. *et al.* Sequence determinants of improved CRISPR sgRNA design. *Genome Res.* **25**, 1147–1157 (2015).
4. Dunn, J. J. & Studier, F. W. Complete nucleotide sequence of bacteriophage T7 DNA and the locations of T7 genetic elements. *J. Mol. Biol.* **166**, 477–535 (1983).
5. Ye, J., McGinnis, S. & Madden, T. L. BLAST: improvements for better sequence analysis. *Nucleic Acids Res.* **34**, W6–9 (2006).
6. Rosa, M. D. Four T7 RNA polymerase promoters contain an identical 23 bp sequence. *Cell* **16**, 815–825 (1979).
7. Corces, M. R. *et al.* An improved ATAC-seq protocol reduces background and enables interrogation of frozen tissues. *Nat. Methods* **14**, 959–962 (2017).
8. Malinin, N. L. *et al.* Defining genome-wide CRISPR–Cas genome-editing nuclease activity with GUIDE-seq. *Nat. Protoc.* **16**, 5592–5615 (2021).
9. Conrad, T., Plumbom, I., Alcobendas, M., Vidal, R. & Sauer, S. Maximizing transcription of nucleic acids with efficient T7 promoters. *Commun Biol* **3**, 439 (2020).
10. Schmittgen, T. D. & Livak, K. J. Analyzing real-time PCR data by the comparative C(T) method. *Nat. Protoc.* **3**, 1101–1108 (2008).
11. Wagih, O. ggseqlogo: a versatile R package for drawing sequence logos. *Bioinformatics* **33**, 3645–3647 (2017).
12. Aznauryan, E. *et al.* Discovery and validation of human genomic safe harbor sites for gene and cell therapies. *Cell Rep Methods* **2**, 100154 (2022).
13. Booeshaghi, A. S., Chen, X. & Pachter, L. A machine-readable specification for genomics assays. *Bioinformatics* **40**, (2024).
