# Supplementary figures and images for: Joint single-cell profiling of Cas9 edits and transcriptomes reveals widespread off-target events and effects on gene expression
Source: bioRxiv. 2025 Aug 28:2025.02.07.636966. Preprint. [Version 2] doi: 10.1101/2025.02.07.636966 (PMC12407703; doi:10.1101/2025.02.07.636966)

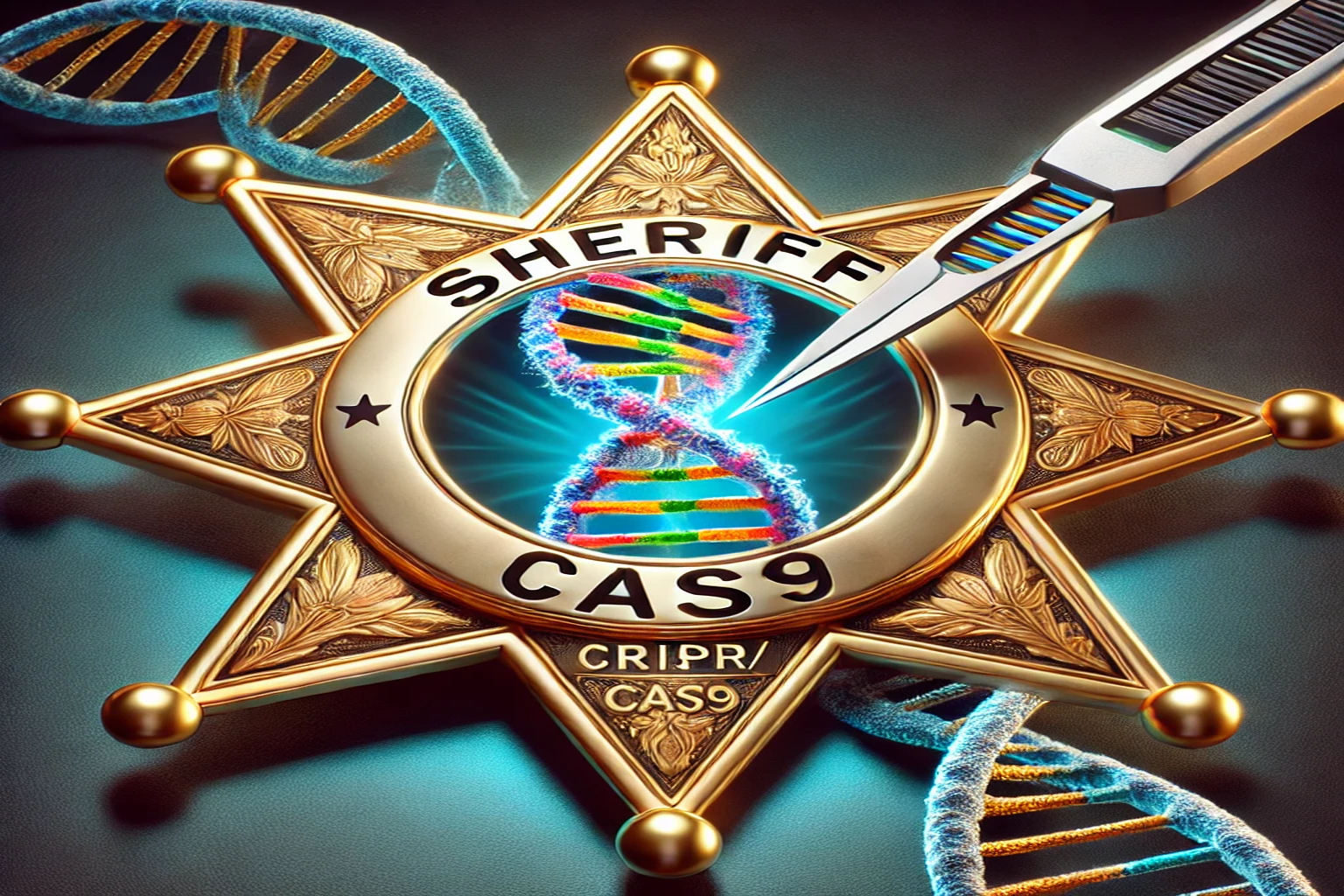

Supplement: Supplement 2 [file media-2.zip › Additional_files/Code/Sheriff-main/img/sheriff.png]

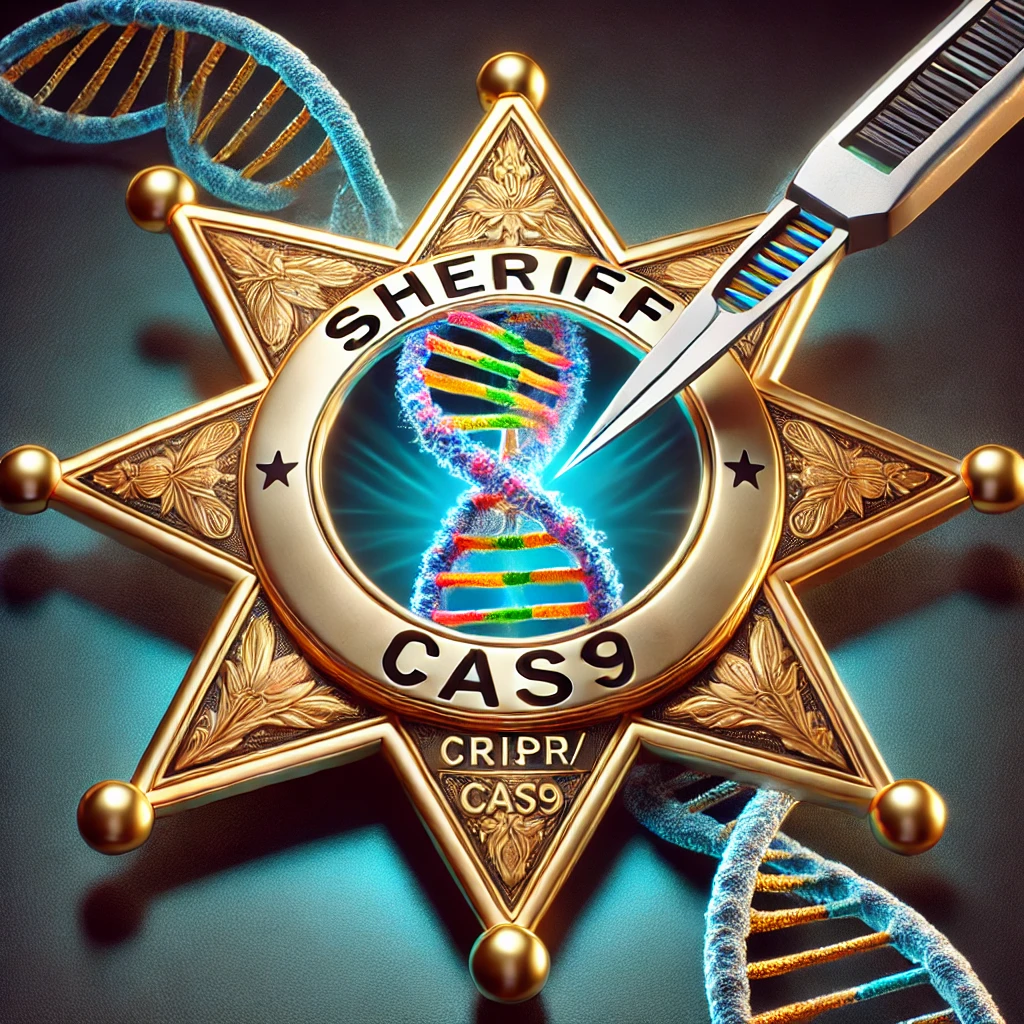

Supplement: Supplement 2 [file media-2.zip › Additional_files/Code/Sheriff-main/img/sheriff_orig.png]
